# Strong Short‐Range Cooperativity in Hydrogen‐Bond Chains

**DOI:** 10.1002/anie.201703757

**Published:** 2017-06-01

**Authors:** Nicholas Dominelli‐Whiteley, James J. Brown, Kamila B. Muchowska, Ioulia K. Mati, Catherine Adam, Thomas A. Hubbard, Alex Elmi, Alisdair J. Brown, Ian A. W. Bell, Scott L. Cockroft

**Affiliations:** ^1^ EaStCHEM School of Chemistry University of Edinburgh, Joseph Black Building David Brewster Road Edinburgh EH9 3FJ UK; ^2^ Afton Chemical Limited London Road Bracknell Berkshire RG12 2UW UK

**Keywords:** cooperativity, hydrogen bonds, noncovalent interactions, supramolecular chemistry

## Abstract

Chains of hydrogen bonds such as those found in water and proteins are often presumed to be more stable than the sum of the individual H bonds. However, the energetics of cooperativity are complicated by solvent effects and the dynamics of intermolecular interactions, meaning that information on cooperativity typically is derived from theory or indirect structural data. Herein, we present direct measurements of energetic cooperativity in an experimental system in which the geometry and the number of H bonds in a chain were systematically controlled. Strikingly, we found that adding a second H‐bond donor to form a chain can almost double the strength of the terminal H bond, while further extensions have little effect. The experimental observations add weight to computations which have suggested that strong, but short‐range cooperative effects may occur in H‐bond chains.

Chains of hydrogen bonds are prevalent structural motifs in supramolecular and biological systems. H bonds are widely proposed to exhibit positive cooperativity,^1^ which may be manifested by a combination of conformational^1^, ^2^ and electronic effects that may make a chain more stable than the sum of its parts.^3^ Such cooperative effects have been shown to influence reactivity,^4^ to contribute to the structure, interactions, and properties of biomolecules and materials,^5^ and to facilitate the communication of chemical information.^6^ H‐bonded water clusters and chains have been isolated in the solid state^7^ and studied experimentally in both liquid and gas phases.^8^ Although many nanoscale and bulk properties may be influenced by the cooperativity of H‐bond networks, it is not possible to directly quantify interaction energies from structural or vibrational characteristics. In addition, discussion of the relative contributions of electrostatics, polarization, and covalency in H‐bond cooperativity5b, 9 is further exacerbated by the challenge of considering the influence of the surrounding solvent.

Herein, we have employed synthetic molecular balances^10^ to directly measure the effect of H‐bond‐chain length on the strength of H‐bonding interactions in solution. At the outset of our investigation we identified the series of phenol, catechol, and pyrogallol (Figure 1 B) as a pertinent model system for examining cooperativity in H‐bond chains. Indeed, H‐bond chains have previously been proposed to contribute to the supramolecular properties of catechol and pyrogallol derivatives.3b, 11 We reasoned that the pre‐organization and proximity of the intramolecular H‐bond donors and acceptors in this series of compounds would minimize conformational entropic effects to allow examination of cooperative electronic influences. Initially we measured the experimental complexation Gibbs energies of phenol, catechol, and pyrogallol with the strong H‐bond acceptor tri‐*n*‐butylphosphine oxide using ^31^P NMR spectroscopy. The binding energies became more favorable as the number of OH groups was increased (Figure 1 A). Such a trend could be rationalized by cooperative effects arising from the formation of a linear intramolecular H‐bond network between the OH groups (Figure 1 B).11b, 11c However, the experimental energetic trend shown in Figure 1 A was not reproduced in DFT energy calculations for the linear binding mode (Figure 1 A; see also solid bars in Figure 3 A). Furthermore, experimental evidence obtained in solution and the solid state indicates that catechol derivatives may bind acceptors in alternative binding modes such as those shown in Figure 1 C.11d, 12 Thus, we side‐stepped this conformational ambiguity by designing a constrained intramolecular system that enabled H‐bond energies to be measured specifically at the end of a chain (Figure 2 A).

**Figure 1:**
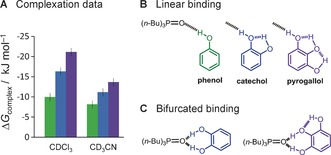
Experimental Gibbs energies for the complexation of tri‐*n*‐butylphosphine oxide with phenol, catechol, and pyrogallol in CDCl_3_ and CD_3_CN. Errors are estimated to be <1 kJ mol^−1^ based on titrations performed in duplicate. Data and additional binding experiments with other phenol derivatives are provided in Table S1.

The strength of intramolecular interactions can be assessed using conformational reporters that act as molecular balances.^10^, ^13^ The molecular balances employed in the present study are based on previous designs that enabled the measurement of solvent and substituent effects on intramolecular interactions (Figure 2 A).^14^ The position of the conformational equilibrium in these new balances enables measurement of the energy of the H bond at the end of a linear chain of one, two, or three H bonds. These molecular balances were synthesized and found to exist in two conformational states on the NMR timescale at room temperature (see the Supporting Information for NMR spectra and minimized structures). Conformers were assigned using 2D NMR spectroscopy and the equilibrium constant *K* was determined by integration of the ^19^F NMR peaks corresponding to each conformer. The difference in the Gibbs energy between the conformers was determined using Δ*G*=−*RT* ln*K*. Balance **1** was found to have a strong preference in CDCl_3_ for the conformation in which the C=O⋅⋅⋅H−O interaction is present (**1 H**; see Figure 2 B). Strikingly, adding a second H bond to form a chain (i.e. going from **1 H** to **2 H)** approximately doubled the measured Δ*G* from −4.2 to −8.1 kJ mol^−1^. However, adding a further H bond to the chain (**2 H** to **3 H)** slightly decreased the preference for the H‐bonded conformer. This unexpected trend was seen to persist in CDCl_3_ solutions containing up to 10 % (v/v) CD_3_CN (Figure 2 B). At higher concentrations of CD_3_CN the conformational Gibbs energies tended to zero due to disruption of the intramolecular H bonds (Table S3).

**Figure 2:**
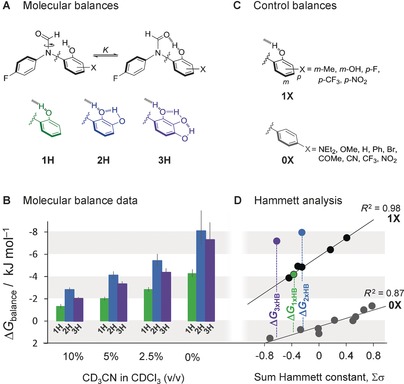
A) Molecular balances and B) conformational Gibbs energies (Δ*G*) measured in solution at 300 K. The H in **1 H** etc. stands for H‐bonded. C) Molecular balances used in the Hammett analysis (D) of substituent effects in H‐bond chains in CDCl_3_. Hammett constants were defined relative to the amide, with *ortho*‐OH groups being approximated by *σ*_p_ (Table S6). Error bars omitted for clarity (Figure S16 shows error bars). Δ*G*_1×HB_, Δ*G*_2×HB_, Δ*G*_3×HB_ approximate the energies associated with chains containing one, two, and three H bonds, respectively.

The data are indicative of a large positive cooperative effect on forming a chain of two H bonds compared to a single H bond, while there is little additional change on further increasing the length of the chain. However, the conformational equilibrium shown in Figure 2 A may be influenced by secondary substituent effects^14^ in addition to the C=O⋅⋅⋅H−O interaction of interest.^15^ These secondary substituent effects were controlled for using the **0 X** and **1 X** series of compounds (Figure 2 C) by plotting the sum of the Hammett constants of the X substituents against the experimental Gibbs energies (Figure 2 D). The **0 X** and **1 X** series formed separate correlations, with the offset approximating the Gibbs energy contribution of a single C=O⋅⋅⋅H−O interaction. The steeper gradient of the **1 X** versus **0 X** data indicates the sensitivity of the C=O⋅⋅⋅H−O interaction to the electronic effects of the X substituents (the more electron‐withdrawing the substituent, the stronger the H bond). The Gibbs energies for compounds **2 H** and **3 H** (blue and purple circles) are vertically displaced from the **0 X** correlation in Figure 2 D by similar amounts (Δ*G*_2×HB_ and Δ*G*_3×HB_), confirming the minimal energetic effect of extending a H‐bond chain beyond two H bonds, even when background substituent effects are taken into account.

We originally envisaged extending the investigation to include 1,2,3,4‐tetrahydroxybenzene derivatives capable of forming a four‐membered H‐bond chain. However, we found that 1,2,3,4‐tetrahydroxybenzene was insufficiently stable and soluble to facilitate NMR titrations, or the onward synthesis of molecular balances. Instead, we established that B3LYP/6–311G* calculated conformational energies (Δ*E*) correlated strongly with experimental Δ*G* values for all of the balances shown in Figure 2 (Figure S18, *R* ^2^=0.99). Thus, we confirmed that computations provided the opportunity to probe situations that could not be examined experimentally to offer insights into the physicochemical origins of the observed short‐range cooperativity. Calculations performed on both the phosphine oxide complexes (Figure 3 A) and balances (Figure 3 B) gave a binary energetic pattern in which there was either one, or more than one, H bond in the linear chain. The calculations also allowed H bonds to be deliberately flipped to break the continuity of the H‐bond chain (hashed bars in Figure 3). The dependence of the energies on the number of H bonds in the chain, rather than on the number of OH groups confirmed that the observed cooperative effects originate from the formation of an intramolecular H‐bond network, and ruled out significant contributions from through‐bond substituent effects. Furthermore, entropic and conformational differences across the compound series could not account for the binary trend observed in both experiments and computations (Tables S4 and S5, Figures S13–S15, S19–S20). Additional calculations in which an external phenol donor could bind in an ideal geometry to the back of the H‐bond chains gave energies (Figure 3 C) similar to those of the intramolecular cases (Figure 3 B). This result confirms that intramolecular geometric constraints do not account for the lack of additional energetic cooperativity on adding a third or fourth H bond to the chain.

**Figure 3:**
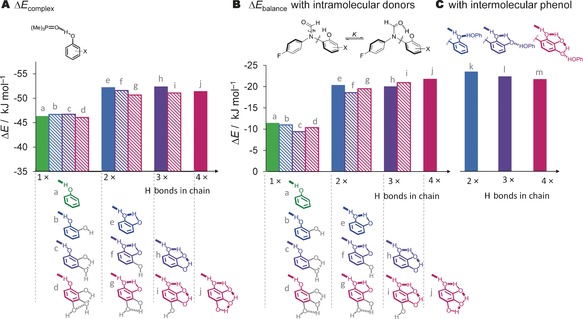
A) Calculated complexation energies of phenol derivatives with a phosphine oxide acceptor and B) conformational energies in molecular balances as the length of the intramolecular OH chain was varied. Solid bars: linear H‐bonded modes (states a, e, h, j); hashed bars: calculated local minima in which the H‐bond chains were deliberately disrupted by flipping the OH groups indicated in gray. C) Calculated conformational energies in molecular balances featuring H‐bond chains terminated by a conformationally free terminal phenol donor. Calculations were performed using B3LYP/6‐311G* and all compound coordinates are provided in the Supporting Information.

A key finding from our experiments (Figure 2 D) and computations (Figure 3) is that adding a second H bond can, depending on context, almost double the strength of the terminal H‐bond interaction. Such doubling of the energy cannot arise exclusively from additive electrostatic field effects since the second H‐bond donor in a chain is positioned further away from the acceptor than the first. Although the limited extent of H‐bond cooperativity on further extension of the chain may seem surprising, it is important to note that we have specifically measured the change in the energy of H bonds at the *end* of the chains. Indeed, our findings add weight to previous computations of water,9b, 16 alcohol,4c and amide chains,^17^ which found that polarizability, molecular dipole moment, charge, and energy all converge much more rapidly at the ends of H‐bond chains than in their middle. This apparent difference can be rationalized as follows: if similar length‐dependent cooperative effects influence both the H‐bond‐donor and ‐acceptor sites, then a site at the middle of a chain will experience two sets of cooperative effects originating from either side of the chain. Therefore, the energetic effect experienced at the center of a chain may be doubled compared to the ends.9b, 17c Similar reasoning may also account in part for the large shifts in the p*K*_a_ value of groups positioned at the center of H‐bond chains.4b However, it is important to note that our findings in neutral H‐bond chains may not extend to situations where charges may exert longer‐range field and inductive effects,4b, 18 or other situations where electron delocalization may play a more important role.9c, 17b, 19

In summary, we have investigated H‐bonding cooperativity in an experimental system in which the geometry and the number of H bonds in a chain were strictly controlled. The strength of the terminal H‐bonding interaction almost doubled on going from one to two H bonds, but further increasing the length of the chain had a negligible energetic effect. Experimental controls and computations confirmed that the observed binary energetic behavior depended entirely on whether a chain of (two or more) H bonds was present, and ruled out significant through‐bond substituent effects. Electrostatics alone do not account for the observed doubling of the interaction energy on forming an H‐bond chain, thereby indicating substantial contributions from inductive polarization. Furthermore, the limited range of the cooperative effect was consistent with previous computations suggesting that polarization changes most rapidly at the ends of H‐bond chains.4c, 9b, 16, 17 Our findings have implications for the fundamental understanding, modeling, and exploitation of H‐bond chains particularly in regard to their role in catalysis,4d and in determining molecular structures and recognition properties.5a, 17c, 20 One might speculate that biology has already explored energetic cooperativity in phenolic H‐bond chains, considering that catechol, not pyrogallol, moieties (Figure 1 B) have been selected by evolution for their adhesive properties.11e, 11f

## Conflict of interest

The authors declare no conflict of interest.

## Supporting information

As a service to our authors and readers, this journal provides supporting information supplied by the authors. Such materials are peer reviewed and may be re‐organized for online delivery, but are not copy‐edited or typeset. Technical support issues arising from supporting information (other than missing files) should be addressed to the authors.

Supplementary
