# Supplementary material for: Strong Short‐Range Cooperativity in Hydrogen‐Bond Chains
Source: Angew Chem Int Ed Engl. 2017 Jun 1;56(26):7658–62. doi: 10.1002/anie.201703757 (PMC5488241; doi:10.1002/anie.201703757)
Supplement: Supplementary file 1 — Supplementary [file ANIE-56-7658-s001.pdf]

Angewandte Chemie
Eine Zeitschrift der Gesellschaft Deutscher Chemiker

Supporting Information

Strong Short-Range Cooperativity in Hydrogen-Bond Chains

*Nicholas Dominelli-Whiteley, James J. Brown, Kamila B. Muchowska, Ioulia K. Mati, Catherine Adam, Thomas A. Hubbard, Alex Elmi, Alisdair J. Brown, Ian A. W. Bell, and Scott L. Cockroft**

anie_201703757_sm_miscellaneous_information.pdf

Table of Contents

1. Host-Guest Binding Studies **S2**

2. Measurement of Conformational Free Energies of Molecular Balances **S7**

3. Van’t Hoff Analyses of Compounds **1H**, **2H**, **3H** **S10**

4. Hammett Analysis **S13**

5. Computational Methods and Data **S14**

6. Synthesis and Compound Characterization **S23**

7. Molecular Balance Conformer Assignment **S71**

8. Supporting References **S90**

1. Host-Guest Binding Studies

NMR spectra were recorded using either a Bruker Ultrashield 400 MHz, heteronuclear, or a Bruker Ascend 500 MHz with Prodigy cryoprobe, heteronuclear.

Prior to performing titrations, pyrogallol and catechol were recrystallized from chloroform, phenol from 20% chloroform in pentane and 4-methoxyphenol from 30% chloroform in hexane and along with all other compounds, dried under vacuum for a minimum of 16 h before use.

Solvents were initially stood over activated 4 Å molecular sieves for a minimum of 24 h and then further dried via passage through a column of activated basic alumina, before being stored over activated 4 Å molecular sieves under nitrogen. Activated basic alumina was purchased as Brockmann grade 1 (Acros Organics) and dried by heating to >275 °C under vacuum for 3 h. NMR tubes, precision glassware and glass syringes used in binding studies were dried under vacuum before use. Titration and dilution experiments were conducted in septum-sealed Willmad cap NMR tubes. Internal standards consisted of the relevant solution sealed in a glass capillary tube inserted into the NMR tube. Internal standards consisted of: 25 mM methylenediphosphonic acid in D_2O for ^{31}P NMR studies, 25 mM trifluoroacetic acid in D_2O or $CDCl_3$ for ^{19}F NMR studies, and C_6D_6 for ^{1}H NMR studies.

350 μL of solvent was added to a vacuum-dried Willmad-cap NMR tube containing a sealed internal standard under an atmosphere of nitrogen. A known amount of equimolar stock-solution of phenol-derivative and tri-*n*-butylphosphine oxide was added and the chemical shift measured via ^{31}P NMR (relative to a 25 mM methylenediphosphonic acid in D_2O internal standard contained in a sealed glass tube within the sample tube). Further equimolar stock-solution of phenol-derivative and tri-*n*-butylphosphine oxide was added and the chemical shift of tri-*n*-butylphosphine oxide measured, repeating the procedure over at least 15 concentration points. The changes in chemical shift on varying the concentration were fitted to a 1:1 binding isoform using the Excel Spreadsheet, 14allMaster.xls kindly supplied by Prof. Christopher A. Hunter FRS (University of Cambridge). The fitted binding curves are shown in Figures S1 to S10. This allowed the accurate determination of the binding constant, K, and thus ΔG via the equation $\Delta G = -RT \ln K$.

Table S1. Experimental binding energies of phenol derivatives H-bond donors to tri-*n*-butylphosphine oxide at 300 K. All values are reported in kJ mol^{-1}.

Donor	Solvent	Δ*G* Result i	Δ*G* Result ii	Mean Δ*G*	Std. Dev
Phenol	$CDCl_3$	-9.9	-10.0	-9.9	0.01
Phenol	CD_3CN	-8.1	-8.1	-8.1	0.00
Catechol	$CDCl_3$	-16.2	-16.5	-16.3	0.19
Catechol	CD_3CN	-11.3	-11.8	-11.5	0.37
Pyrogallol	$CDCl_3$	-21.5	-20.8	-21.1	0.50
Pyrogallol	CD_3CN	-13.7	-13.6	-13.6	0.10
4-Methoxyphenol	$CDCl_3$	-8.8	-9.8	-9.3	0.65
4-Methoxyphenol	CD_3CN	-7.6	-8.0	-7.8	0.26
2-Methoxyphenol	$CDCl_3$	+0.4	-0.5	-0.1	0.58
2-Methoxyphenol	CD_3CN	-3.4	-3.5	-3.5	0.09

Binding Isotherm Plots for Host-Guest Systems.

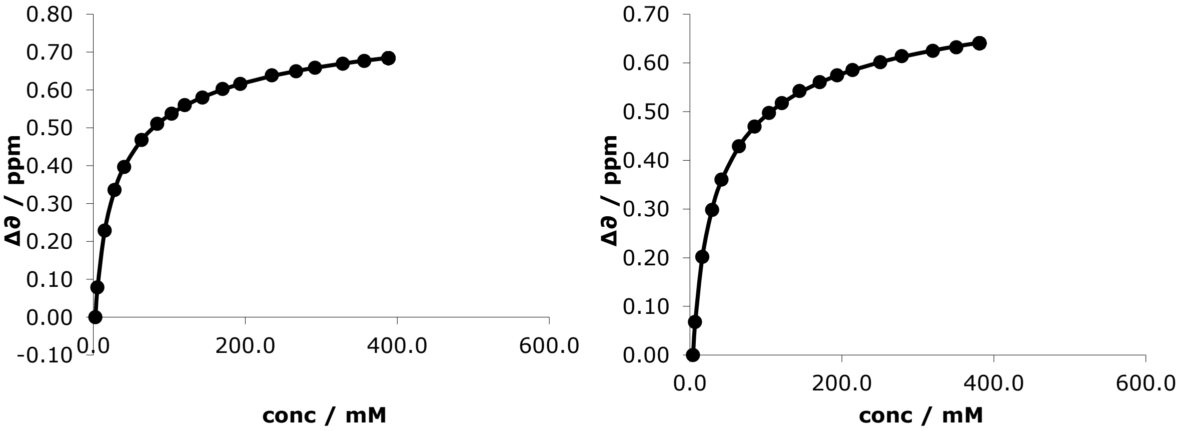

Figure S1. Dilutions of 1:1 Phenol: Tri-*n*-butylphosphine oxide in $CDCl_3$.

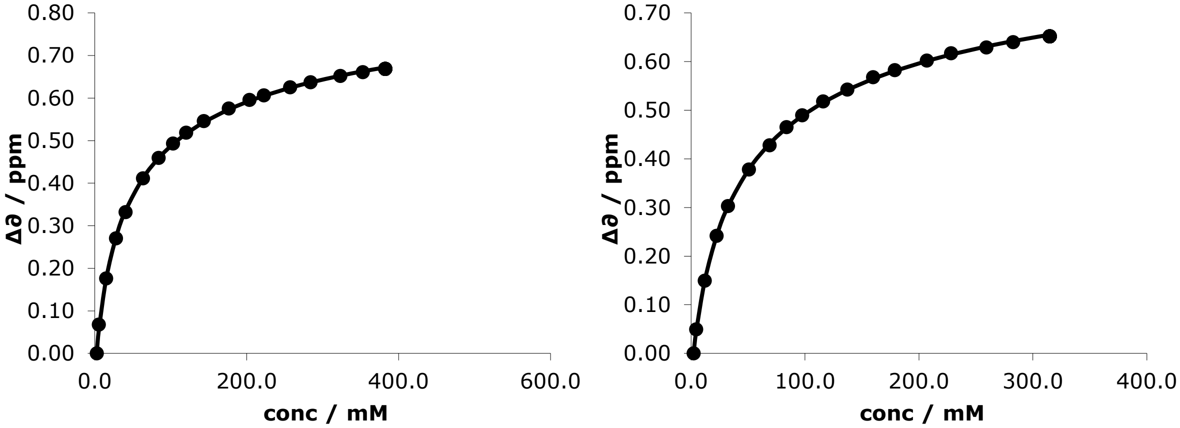

Figure S2. Dilutions of 1:1 Phenol : Tri-*n*-butylphosphine oxide in CD_3CN.

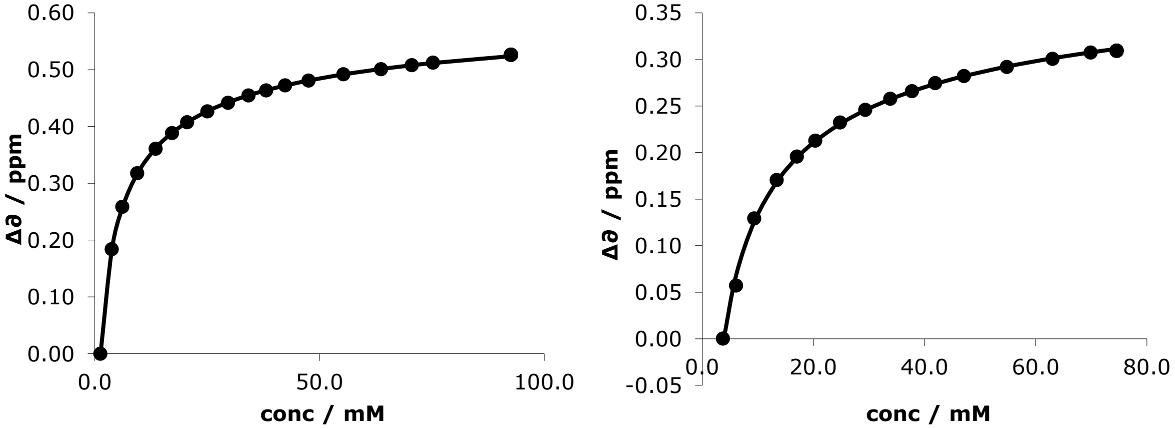

Figure S3. Dilutions of 1:1 Catechol : Tri-*n*-butylphosphine oxide in $CDCl_3$.

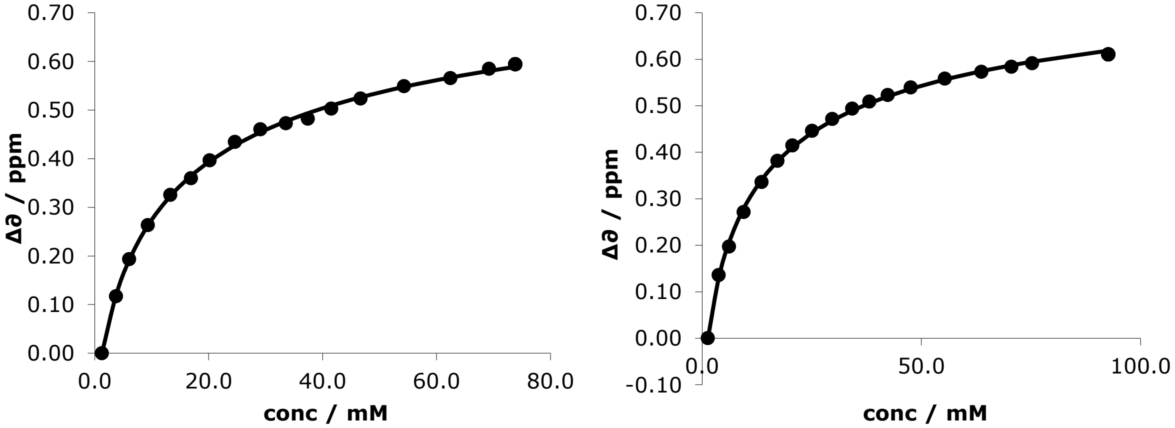

Figure S4. Dilutions of 1:1 Catechol : Tri-*n*-butylphosphine oxide in CD_3CN.

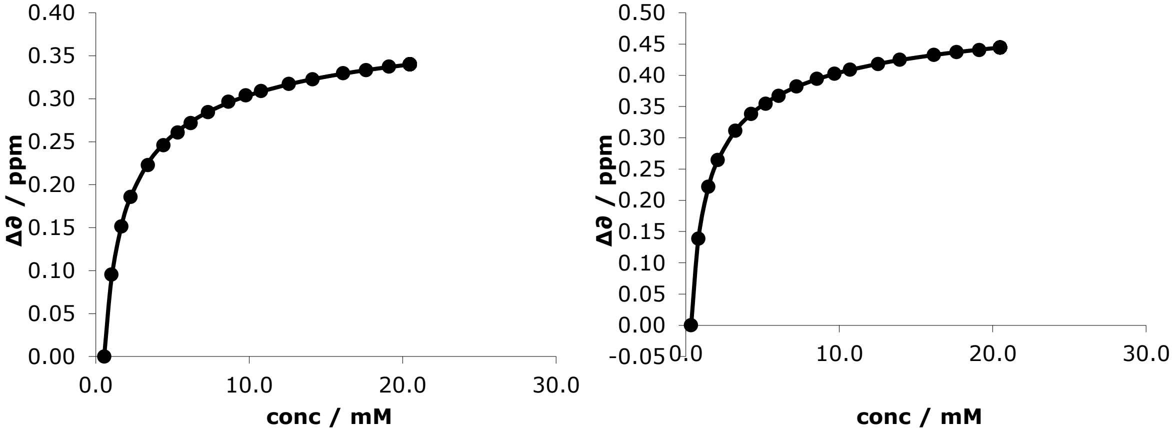

Figure S5. Dilutions of 1:1 Pyrogallol : Tri-*n*-butylphosphine oxide in $CDCl_3$.

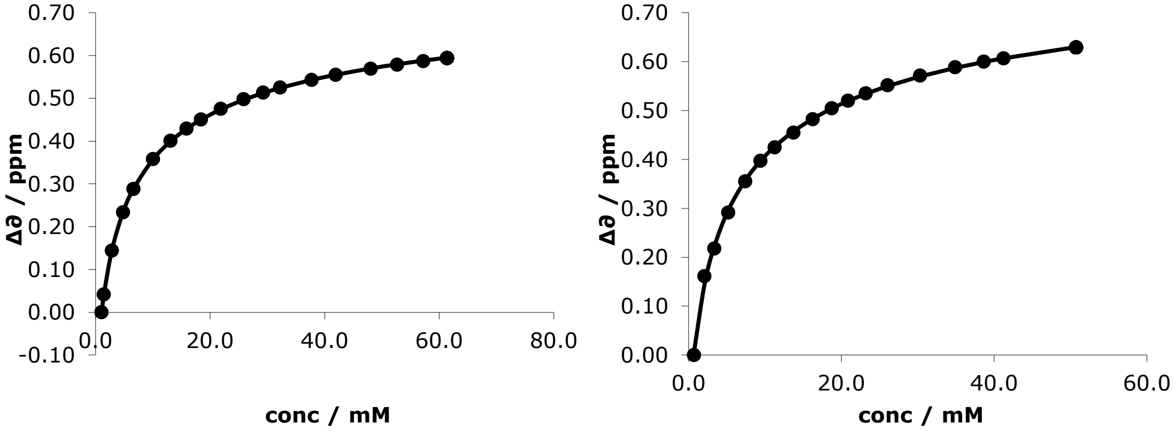

Figure S6. Dilutions of 1:1 Pyrogallol : Tri-*n*-butylphosphine oxide in CD_3CN.

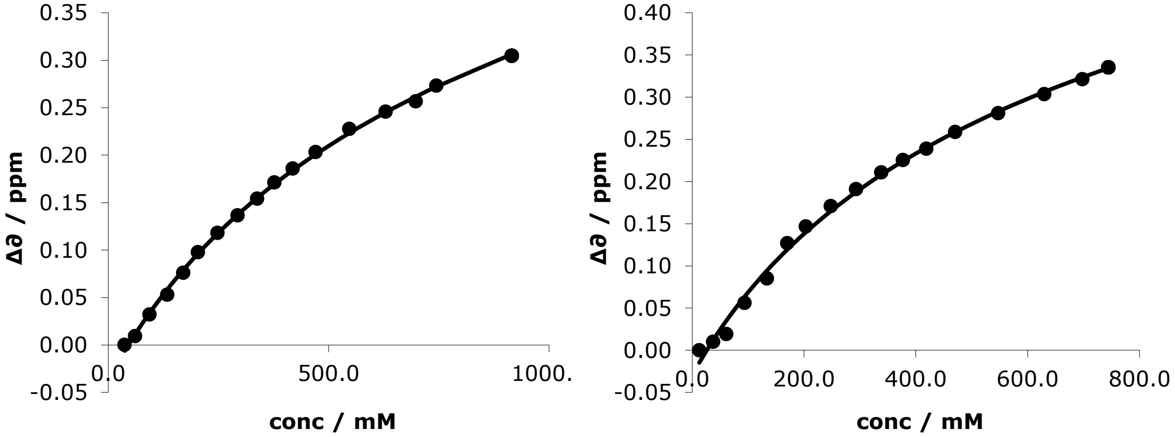

Figure S7. Dilutions of 1:1 2-Methoxyphenol : Tri-*n*-butylphosphine oxide in $CDCl_3$.

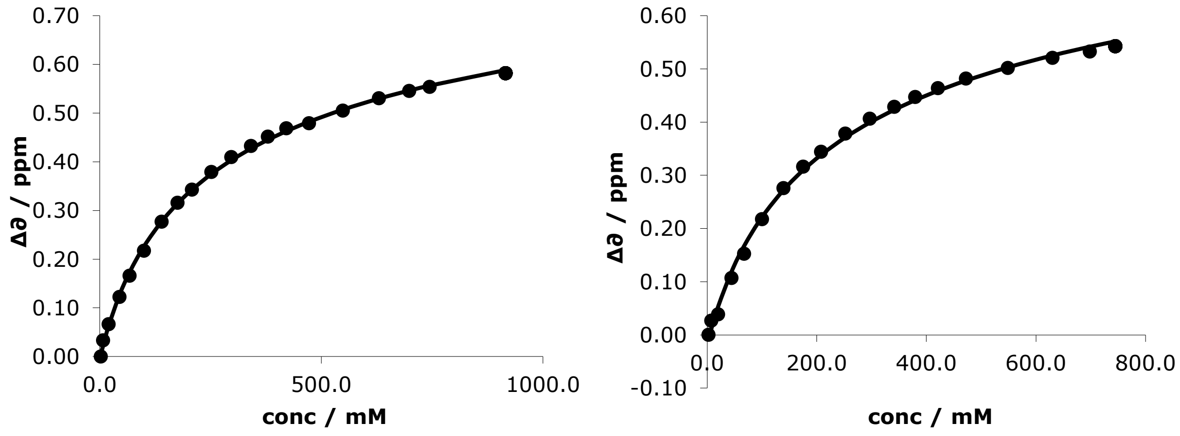

Figure S8. Dilutions of 1:1 2-Methoxyphenol : Tri-*n*-butylphosphine oxide in CD_3CN.

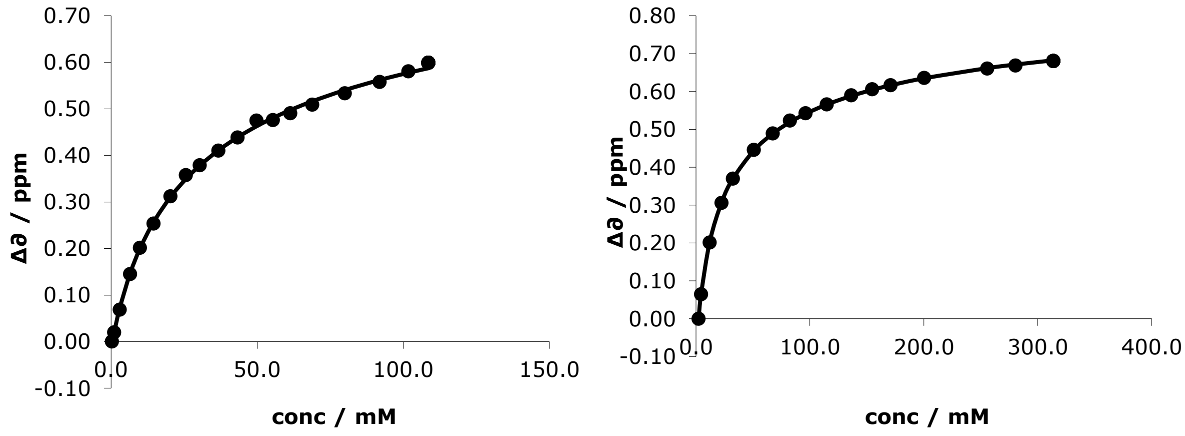

Figure S9. Dilutions of 1:1 4-Methoxyphenol : Tri-*n*-butylphosphine oxide in $CDCl_3$.

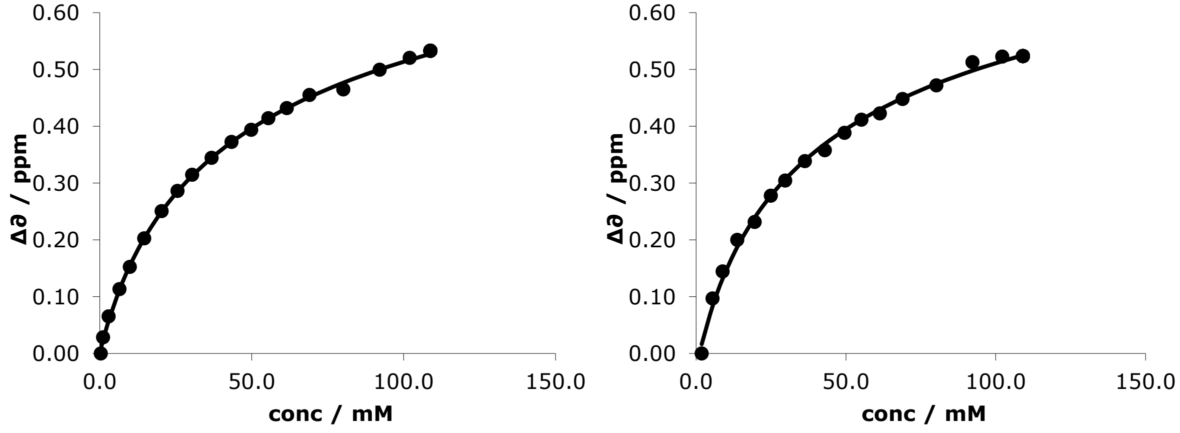

Figure S10. Dilutions of 1:1 4-Methoxyphenol : Tri-*n*-butylphosphine oxide in CD_3CN.

2. Measurement of Conformational Free Energies of Molecular Balances

NMR spectra were recorded using either a Bruker Ultrashield 400 MHz, heteronuclear, or a Bruker Ascend 500 MHz with Prodigy cryoprobe, heteronuclear. Conformational free energies of the molecular balances were determined by ^{19}F NMR spectroscopy. Samples of molecular balances were prepared at the concentrations specified in dried solvent (see host-guest binding studies) and the conformer integral ratio determined by ^{19}F NMR (2048 scans). The integral ratio of the conformers was used to determine the conformational equilibrium constant, *K*. Conformational free energies were then calculated using the equation $\Delta G = -RT\ln K$, where *K* is defined according to the equilibrium shown in Figure 1. Synthesis, characterization and experimental data for the **0X** series of compounds were reported in references.[1, 2] Conformers were assigned as detailed in the Synthesis and Characterization section below. A conservative integration error estimate of ±0.025 in the minor conformer NMR peak integration when the major conformer relative integral was set to 1.000 (*i.e.* the integration ratio was $[\int_{\text{major conformer}} = 1]/[\int_{\text{minor conformer}} \pm 0.025]$) was applied, resulting in asymmetric ΔG_{exp} error margins as listed in Table S2 and plotted on figures. An example of determination of extreme conformer ratios is shown on page S35.

Table S2. Conformational free energies ΔG_{exp} of molecular balances determined at 300 K in $CDCl_3$.

	**Balance concentration**						
	**0.75 mM**	**1 mM**	**1.5 mM**	**Mean**	**2x Std. Dev.**	**Error+**	**Error-**
**Balance**	**Conformational free energies in $CDCl_3$/ kJ mol^{-1}**						
**1-H**	-4.2	-4.2	-4.3	-4.3	0.1	0.3	0.4
**1-*p*-NO₂**	-7.8	-7.6	-7.5	-7.6	0.4	1.1	1.9
**1-*p*-CF₃**	-6.6	-6.6	-6.4	-6.5	0.2	0.7	1.1
**1-*m*-OH**	-5.1	-5.0	n.d.	-5.1	0.1	0.4	0.5
**1-*m*-Me**	-3.9	-4.0	-3.9	-4.0	0.1	0.3	0.3
**1-*p*-F**	-5.0	-5.1	-5.0	-5.0	0.2	0.4	0.5
**2-H**	-8.0	-8.2	-8.2	-8.1	0.1	1.2	2.6
**3-H**	-7.4	-7.3	-7.3	-7.3	0.1	1.0	1.6
**1-Alk**	-6.3	-6.5	-6.4	-6.4	0.2	0.9	0.7
**0-Alk**	-4.7	-4.7	-4.7	-4.7	0.1	0.5	0.4
**2-Ar**	-3.0	-2.9	-2.8	-2.9	0.2	0.2	0.2

n.d. = not determined due to low solubility.

Table S3. Conformational free energies ΔG_{exp} of molecular balances determined at 300 K in $CD_3CN/CDCl_3$ solutions. Errors are ±0.2 kJ mol^{-1} in all cases where ΔG_{exp} is <-3.4 kJ mol^{-1} and < ±0.6 kJ mol^{-1} in all other cases.

	% (v/v) CD_3CN in $CDCl_3$						
	**2.5%**	**5%**	**10%**	**20%**	**30%**	**50%**	**100%**
**Balance**	**Conformational free energies at 1 mM / kJ mol^{-1}**						
**1-H**	-2.8	-2.0	-1.3	-0.8	-0.8	-1.0	-0.4
**1-*p*-NO$_2$**	-4.0	-3.0	-2.2	-1.5	-1.3	-1.2	-1.3
**1-*p*-CF$_3$**	-3.8	-2.9	-2.1	-1.3	-1.2	-1.0	n.d.
**1-*m*-Me**	-2.8	-2.0	-1.3	-0.7	-0.4	-0.3	-0.4
**1-*p*-F**	-2.9	-2.1	-1.3	-0.8	-0.6	-0.6	n.d.
**2-H**	-5.4	-4.1	-2.8	-1.6	-1.1	-0.7	-0.5
**3-H**	-4.4	-3.3	-2.0	-1.1	-0.6	-0.3	0.0
**1-Alk**	-5.8	-5.3	-4.5	-4.1	-3.6	-3.2	-2.8
**2-Ar**	-1.0	-0.4	-0.0	0.2	-0.2	-0.1	0.5

n.d. = not determined due to peak overlap in NMR spectra.

Dilution of 1H in $CDCl_3$

It was anticipated that balances containing both H-bond acceptors and donors might dimerize in apolar solvents. Thus, a dilution experiment was carried out on balance **1H** to determine whether dimerization might influence the conformational free energies. To a vacuum-dried Willmad-cap NMR tube containing a sealed internal standard (0.025 M TFA in $CDCl_3$) was added $CDCl_3$ (350 µL). *N*-(4-fluorophenyl)-*N*-(2,3,4-trihydroxyphenyl)formamide in $CDCl_3$ solution (2 µL, 75 mM) was added and the ^{19}F NMR recorded. Further solution was added and this process repeated to obtain 17 spectra at increasing concentrations. The chemical shifts of the fluorine peaks corresponding to both conformers were fitted to a dimerization model using the Excel Spreadsheet, 14allMaster.xls kindly supplied by Prof. Christopher A. Hunter FRS (University of Cambridge). A dimerization constant of K_{dime} = 0.7 M^{-1} was determined for the major conformer in which the internal H-bond was formed (Figure S11) and K_{dime} = 6.8 M^{-1} for the minor conformer lacking the internal H-bond (Figure S12). The largest dimerization constant corresponded to <2% dimerization at 1.5 mM, confirming that dimerization was likely to have a negligible influence on the free energies measured at concentrations below 1.5 mM. Compounds **2H** and **3H** possessed insufficient solubility to allow the determination of accurate binding dimerization constants. Thus, as an additional experimental control, the conformational free energy of each balance was determined in $CDCl_3$ at concentrations of 0.75, 1 and 1.5 mM and the average of these three values used in subsequent analyses (Tables S2-S3). Tables S2-S3 show that there is very little deviation in the free energy values over these three concentrations for all compounds examined, consistent with dimerization having a minimal influence on the conformational free energies at the low concentrations

employed. Previous work has shown even lower extent of dimerization in the case of the **0X** series of balances.[1, 2] Due to the competitive binding of CD_3CN to H-bonding groups and the resultant weakening of dimerization, conformational free energies determinations in CD_3CN mixtures were measured at a balance concentration of 1 mM.

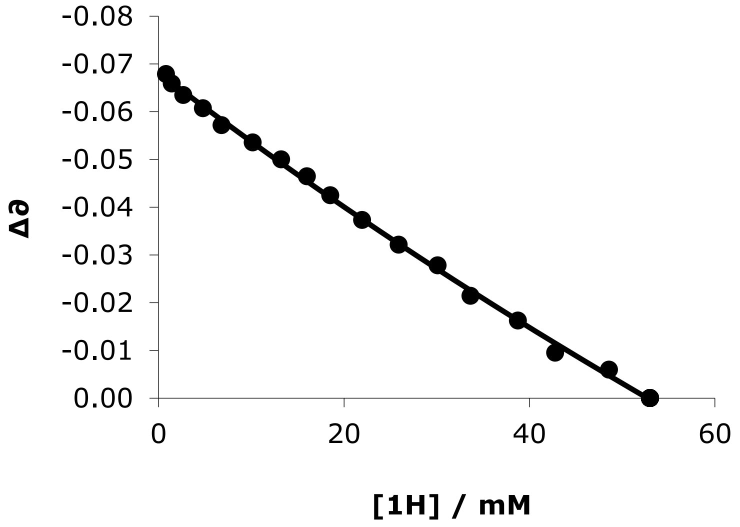

Figure S11. Dilution of balance **1H** in $CDCl_3$ showing the change in fluorine chemical shift for the dominant H-bonded conformer.

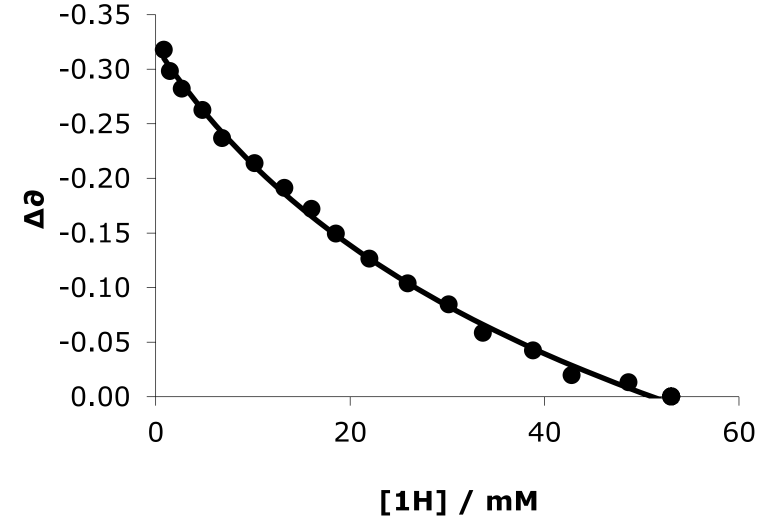

Figure S12. Dilution of balance **1H** in $CDCl_3$ showing the change in fluorine chemical shift for the minor non-H-bonded conformer.

3. Van't Hoff Analyses of compounds 1H, 2H and 3H.

Van't Hoff analysis was carried out on 1 mM samples of compounds **1H**, **2H** and **3H** in dried $CDCl_3$. Samples were prepared and placed in an air-tight Wilmad-cap NMR tube. Spectra were obtained at a minimum of seven temperatures, beginning with the coldest. Samples were equilibrated at each temperature for 30 minutes within the spectrometer. Results are shown in Tables S4-S5 and Figures S13-15 showing the derivation of thermodynamic parameters from the gradient and intercept according to the equation $\Delta G = \Delta H - T\Delta S$.

Table S4. Van't Hoff Analysis (**1H, 2H, 3H**)

T/ K	1/T	ln K_{1H}	ln K_{2H}	ln K_{3H}
278.2	0.00359	1.945	3.576	3.297
282	0.00355	1.877	3.540	3.194
286	0.00350	1.814	3.442	3.079
290	0.00345	1.784	3.381	3.037
294	0.00340	1.737	3.324	2.996
298	0.00336	1.704	3.297	2.976
300	0.00333	1.671	3.219	2.900
305	0.00328	1.655	3.170	3.194

Table S5. Dissection of ΔH and $T\Delta S$ at 298 K.

Balance	$-T\Delta S$ / kJ mol^{-1}	ΔH / kJ mol^{-1}	ΔG / kJ mol^{-1}
**1H**	3.4	-7.6	-4.2
**2H**	2.8	-10.9	-8.1
**3H**	3.1	-10.5	-7.4

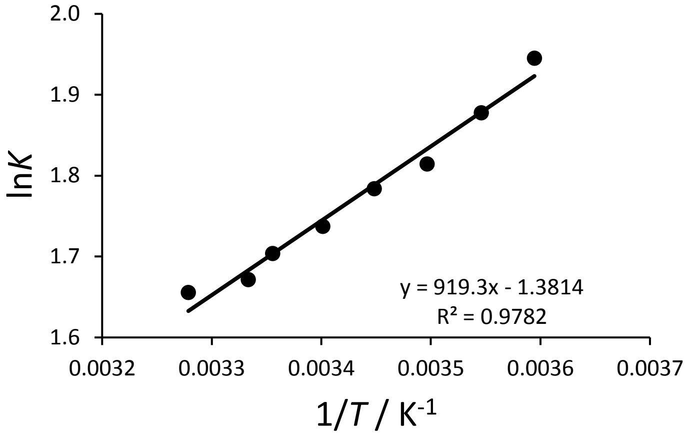

Figure S13. van’t Hoff analysis for compound **1H**.

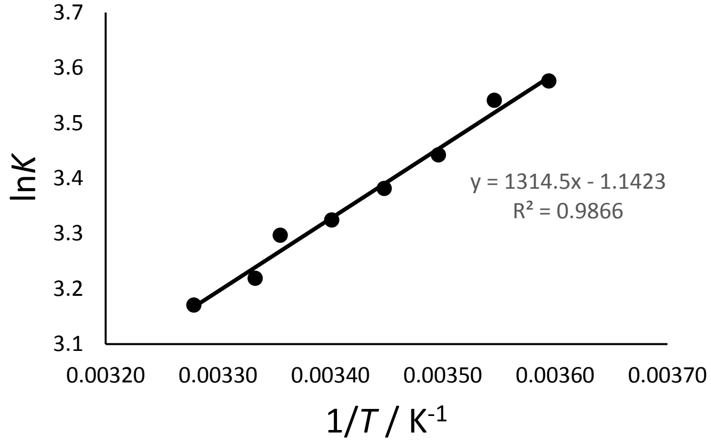

Figure S14. van’t Hoff analysis for compound **2H**.

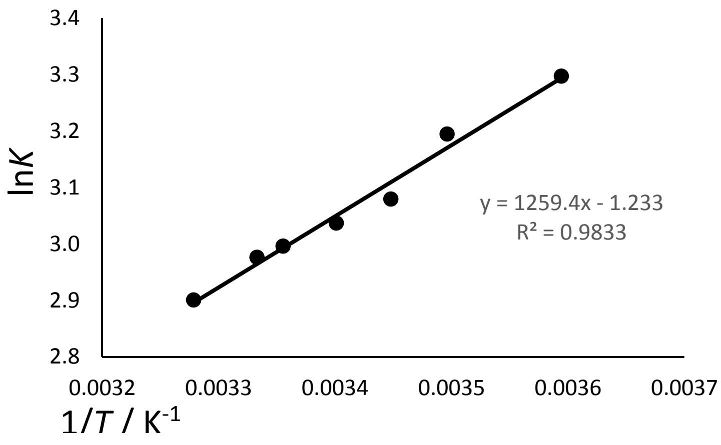

Figure S15. van’t Hoff analysis for compound **3H**.

4. Hammett Analysis

Table S6. Hammett Constants vs Δ*G* measured in $CDCl_3$ at 300 K.

Balance	Δ*G* / kJ mol^{-1}	σ_{ortho} (used σ_p)	σ_{meta}	σ_{para}	Σσ
**1-H**	-4.3	-0.37	0	0	-0.37
**2-H**	-8.1	-0.37	+0.12	0	-0.25
**3-H**	-7.3	-0.37	+0.12	-0.37	-0.62
**1-*m*-Me**	-4.0	-0.37	-0.07	0	-0.44
**1-*m*-OH**	-5.1	-0.37	0.12	0	-0.25
**1-*p*-F**	-5.0	-0.37	0	+0.06	-0.31
**1-*p*-CF_3**	-6.5	-0.37	0	+0.54	+0.17
**1-*p*-NO_2**	-7.6	-0.37	0	+0.78	+0.41
**0-NEt_2**	+1.6	0	0	-0.72	-0.72
**0-OMe**	+0.8	0	0	-0.27	-0.27
**0-H**	+1.1	0	0	0	0
**0-Ph**	+0.7	0	0	-0.01	-0.01
**0-Br**	-0.1	0	0	+0.23	+0.23
**0-COMe**	-0.1	0	0	+0.50	+0.50
**0-CN**	-1.0	0	0	+0.66	+0.66
**0-CF_3**	-0.4	0	0	+0.54	+0.54
**0-NO_2**	-1.4	0	0	+0.78	+0.78
**2-Ar**$^{\neq}$	-2.9	-0.37	0.05	0	-0.32

$^{\neq}$ The Hammett constant, σ_m, of the 2-hydroxyphenol substituent was estimated using σ_m of a 4-methoxyphenyl substituent.

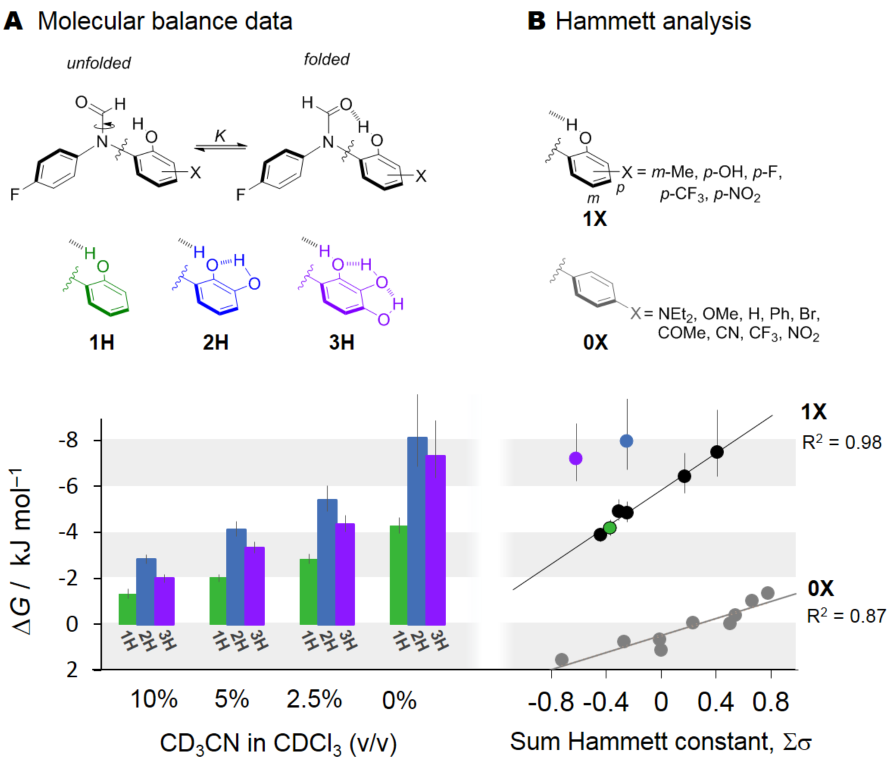

Figure S16. Version of main text Figure 2 with error bars. Error bars (± 0.2 kJ mol^{-1}) for **0X** series (grey dots) lie within the points.

5. Computational Methods and Data

Molecular torsion balance equilibrium geometries and energies were calculated in the gas-phase using Spartan ’14 at the levels of theory specified. Starting geometries for full minimizations of molecular balances were determined using an unconstrained equilibrium conformer search. Local minima of the unfolded conformer (without the internal CO···HO bond) were determined by taking this structure, rotating the formyl C-N bond by 180°, and performing a second equilibrium conformer search subject to this dihedral-angle constraint. The resulting structures in each formyl conformer were then re-subjected to an unconstrained equilibrium geometry calculation to obtain the local minimum geometry at the relevant level of theory. ΔE was determined as the difference in energy between the conformers in which the formyl oxygen (O conf, major) vs. formyl proton (H conf, minor) laid over the X-substituted ring. Calculations were confirmed to represent energy minima by an absence of imaginary vibrational frequencies.

Table S7. Calculated gas-phase conformational energies for molecular balances, ΔE_{bal}

Balance	B3LYP/6-311G*			B3LYP/aug-cc-pVDZ		
	E (O conf) / kJ mol⁻¹	*E* (H conf) / kJ mol⁻¹	Δ*E* / kJ mol⁻¹	*E* (O conf) / kJ mol⁻¹	*E* (H conf) / kJ mol⁻¹	Δ*E* / kJ mol⁻¹
**1-H**	-2117820.12	-2117808.69	-11.4	-2117602.88	-2117590.69	-12.2
**2-H**	-2315370.97	-2315350.63	-20.3	-2315147	-2315127.16	-19.8
**3-H**	-2512913.27	-2512893.24	-20.0	-2512683.94	-2512664.24	-19.7
**4-H**	-2710449.79	-2710428.05	-21.7	-2710214.6	-2710192.61	-22.0
**1-*m*-Me**	-2221067.11	-2221056.24	-10.9	-2220836.07	-2220825.03	-11.0
**1-*m*-OH**	-2315351.59	-2315338.75	-12.8	-2315130.51	-2315117.36	-13.2
**1-*p*-F**	-2378441.72	-2378429.43	-12.3	-2378184.27	-2378171.06	-13.2
**1-*p*-CF_3**	-3002983.05	-3002967.67	-15.4	-3002630.44	-3002614.85	-15.6
**1-*p*-NO_2**	-2654884.44	-2654864.71	-19.7	-2654616.34	-2654594.57	-21.8
**0-NEt_2**	-2478555.63	-2478560.1	+4.5	-2478281.61	-2478284.79	+3.2
**0-OMe**	-2221018.38	-2221020.56	+2.2	-2220772.66	-2220774.6	+1.9
**0-H**	-1920264.76	-1920265.67	+0.9	-1920054.79	-1920055.81	+1.0
**0-Ph**	-2527024.91	-2527026.23	+1.3	-2526754.82	-2526756.78	+2.0
**0-Br**	-8677103.3	-8677102.88	-0.4	-8676925.98	-8676925.89	-0.1
**0-COMe**	-2321141.48	-2321139.68	-1.8	-2320882.32	-2320881.44	-0.9
**0-CN**	-2162510.79	-2162507.67	-3.1	-2162266.74	-2162264.86	-1.9
**0-CF_3**	-2805428.82	-2805427.13	-1.7	-2805082.95	-2805081.94	-1.0
**0-NO_2**	-2457333.22	-2457329.37	-3.9	-2457071.06	-2457069.04	-2.0
**Balance**	**M062X/6-311G***					
	E (O conf) / kJ mol⁻¹	*E* (H conf) / kJ mol⁻¹	Δ*E* / kJ mol⁻¹			
**1-H**	-2117011.47	-2117003.98	-7.5			
**2-H**	-2314504.44	-2314488.32	-16.1			
**3-H**	-2511988.54	-2511973	-15.5			
**4-H**	-2709467.49	-2709449.89	-17.6			
**1-*m*-Me**	-2220206.25	-2220198.17	-8.1			
**1-*m*-OH**	-2314484.72	-2314474.49	-10.2			
**1-*p*-F**	-2377564.38	-2377555.6	-8.8			
**1-*p*-CF_3**	-3001925.3	-3001913.66	-11.6			
**1-*p*-NO_2**	-2653883.99	-2653866.56	-17.4			
**0-NEt_2**	-2477550.77	-2477554.64	+3.9			
**0-OMe**	-2220156.59	-2220158.82	+2.2			
**0-H**	-1919514.52	-1919516.43	+1.9			
**0-Ph**	-2526031.61	-2526032.61	+1.0			
**0-Br**	-8676433.09	-8676432.22	-0.9			
**0-COMe**	-2320236.36	-2320234.88	-1.5			
**0-CN**	-2161688.25	-2161685.3	-3.0			
**0-CF_3**	-2804429.61	-2804427.62	-2.0			
**0-NO_2**	-2456391.33	-2456387.79	-3.5			

Table S8. Calculated (B3LYP/6-311G*) gas-phase thermodynamic data for molecular balances

Balance	Δ*E* / kJ mol^{-1}	Δ*H*/ kJ mol^{-1}	Δ*G* / kJ mol^{-1}
**1-H**	-11.4	-10.9	-9.5
**2-H**	-20.3	-19.7	-17.3
**3-H**	-20.0	-18.5	-18.5
**4-H**	-21.7	-20.6	-17.5
**1-*m*-Me**	-10.9	-10.8	-9.6
**1-*m*-OH**	-12.8	-12.0	-10.4
**1-*p*-F**	-12.3	-11.7	-10.5
**1-*p*-CF_3**	-15.4	-14.2	-12.4
**1-*p*-NO_2**	-19.7	-19.2	-17.7
**0-NEt_2**	+4.5	4.5	4.9
**0-OMe**	+2.2	2.2	2.5
**0-H**	+0.9	1.5	1.7
**0-Ph**	+1.3	1.1	1.0
**0-Br**	-0.4	-0.9	-0.9
**0-COMe**	-1.8	-2.1	-2.1
**0-CN**	-3.1	-3.2	-3.3
**0-CF_3**	-1.7	-2.1	-2.2
**0-NO_2**	-3.9	-3.6	-3.6

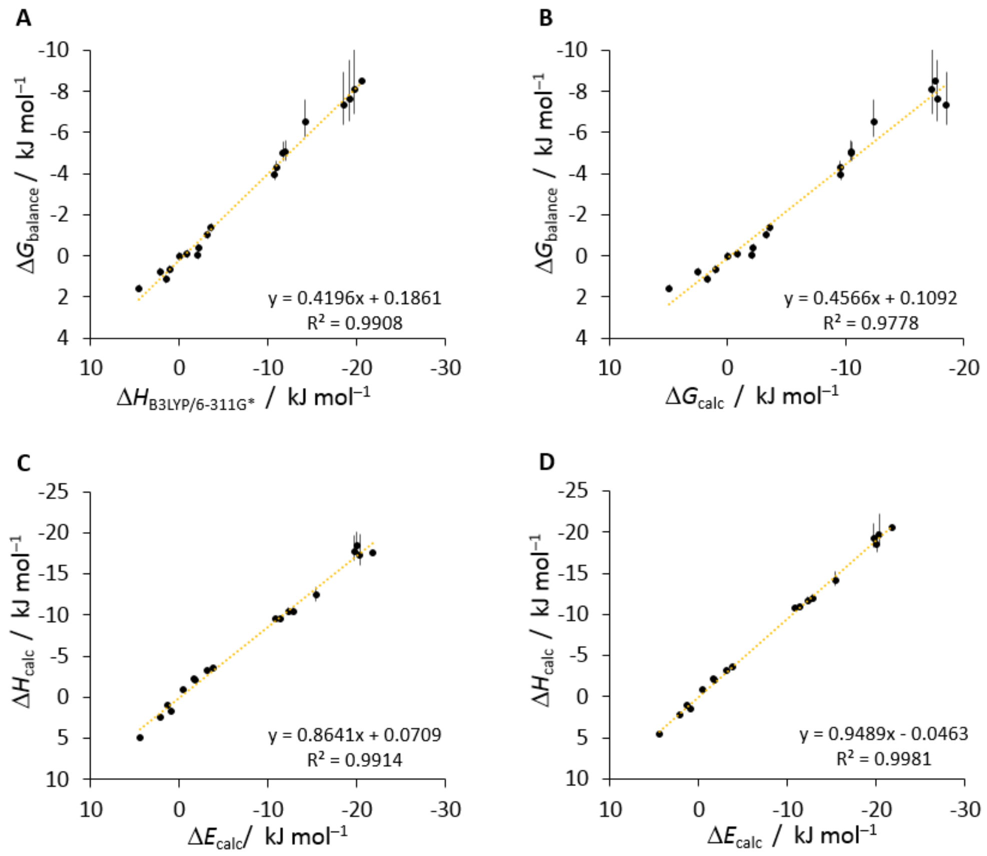

Figure S17. Correlations of calculated (B3LYP/6-311G*) and experimental thermodynamic parameters for the **0X**, **1X**, **2H**, **3H** and **4H** series of balances. (A) Experimental conformational free energy ($CDCl_3$) *vs*. calculated enthalpies from frequency calculation. (B) Experimental conformational free energy ($CDCl_3$) *vs*. Gibbs free energy calculated from frequency calculation. (C) Calculated enthalpy *vs*. calculated (scf) conformational free energy difference. (D) Calculated Gibbs free energy *vs*. calculated (scf) free energy difference.

Table S9. Calculated gas-phase conformational energies for molecular balances with turned H-bonds (Fig. 3)

Balance	Number of H-bonds in chain	B3LYP/6-311G*			B3LYP/aug-cc-pVDZ		
		E (O conf) / kJ mol^{-1}	*E* (H conf) / kJ mol^{-1}	Δ*E* / kJ mol^{-1}	*E* (O conf) / kJ mol^{-1}	*E* (H conf) / kJ mol^{-1}	Δ*E* / kJ mol^{-1}
**2H**	**1**	-2315341.96	-2315330.98	-11.0	-2315122.72	-2315110.94	-11.8
**3H**	**1**	-2512882.75	-2512873.35	-9.4	-2512658.17	-2512647.96	-10.2
**4H**	**1**	-2710419.03	-2710408.68	-10.4	-2710187.89	-2710176.36	-11.5
**3H**	**2**	-2512892.55	-2512873.97	-18.6	-2512666.87	-2512648.61	-18.3
**4H**	**2**	-2710429.00	-2710409.5	-19.5	-2710196.74	-2710177.35	-19.4
**4H**	**3**	-2710430.13	-2710409.19	-20.9	-2710197.94	-2710176.81	-21.13
**Balance**	**Number of H-bonds in chain**	**M06-2X/6-311G***					
		E (O conf) / kJ mol^{-1}	*E* (H conf) / kJ mol^{-1}	Δ*E* / kJ mol^{-1}			
**2H**	**1**	-2314475.74	-2314467.69	-8.1			
**3H**	**1**	-2511958.73	-2511951.73	-7.0			
**4H**	**1**	-2709437.71	-2709428.55	-9.2			
**3H**	**2**	-2511967.03	-2511952.36	-14.7			
**4H**	**2**	-2709445.63	-2709429.54	-16.1			
**4H**	**3**	-2709446.90	-2709429.63	-17.3			

OH groups were flipped 180° to create the specified number of H-bonds in a chain to the formamide (Fig. 3)

Table S10. Calculated gas-phase conformational energies for molecular balances minimized with a terminal intermolecular phenol molecule acting as a H-bond donor. Data are plotted in Figure 3C in the main text.

Balance	Number of H-bonds in chain	B3LYP/6-311G*		
		E (O conf) / kJ mol^{-1}	*E* (H conf) / kJ mol^{-1}	Δ*E* / kJ mol^{-1}
**1H + phenol**	**2**	-2925298.65	-2925274.91	-23.7
**2H + phenol**	**3**	-3122841.25	-3122818.59	-22.7
**3H + phenol**	**4**	-3320383.01	-3320361.00	-22.0

BSSE (Counterpoise) Corrected Interaction Energies for Complexes

BSSE (Counterpoise) corrected interaction energies were calculated using Gaussian '09[3] at the B3LYP level of theory. Initially, the complex of trimethylphosphine oxide with each compound/chain was subjected to a geometry minimization using the specified basis set. The interaction energy between the phosphine oxide and the rest of each of the complexes (i.e. an input where all atoms of the phenol derivative = fragment 1 and all atoms of trimethylphosphine oxide = fragment 2) was then determined using the counterpoise method to account for basis set superposition errors (BSSE), and the values are listed in Table S11. All calculations were determined to be minima by the lack of imaginary vibrational frequencies.

Table S11. Counterpoise-corrected H-bond energies with a $POMe_3$ H-bond acceptor as the number of members in the H-bond chain was varied.

Complex (with $POMe_3$)	Basis set (B3LYP/)	Int. Energy$_{1HB}$ / kJ mol^{-1}	Int. Energy$_{2HB}$ / kJ mol^{-1}	Int.Energy$_{3HB}$ / kJ mol^{-1}	Int. Energy$_{4HB}$ / kJ mol^{-1}
**Phenol**	6-311G*	**-46.3**	**-52.2**	**-52.4**	**-51.4**
	aug-cc-pVDZ	**-47.8**	**-53.9**	**-54.4**	**-53.3**
**Tetrahydroxybenzene**	6-311G*	-46.1	-50.7	-51.1	**-51.4**
OHs Turned	aug-cc-pVDZ	-	-52.8	-53.2	**-53.3**
**Pyrogallol**	6-311G*	-46.7	-51.6	**-52.4**	N/A
OHs **Turned**	aug-cc-pVDZ	-	-53.9	**-54.4**	N/A
**Catechol**	6-311G*	-46.7	**-52.2**	N/A	N/A
OHs Turned	aug-cc-pVDZ	-49.5	**-53.9**	N/A	N/A

Phenol derivatives: 1HB: Phenol, 2HB: catechol, 3HB: pyrogallol, 4HB: tetrahydroxybenezne. Numbers in bold represent true energetic minima while others are: "OHs turned" which represent the compound specified with OH groups either aligned to create an H-bond network or spun 180° to create the specified length of H-bond network. Structural data for these complexes are presented in Figures S19 and S20.

Versions of Computational Figures at Alternative Levels of Theory

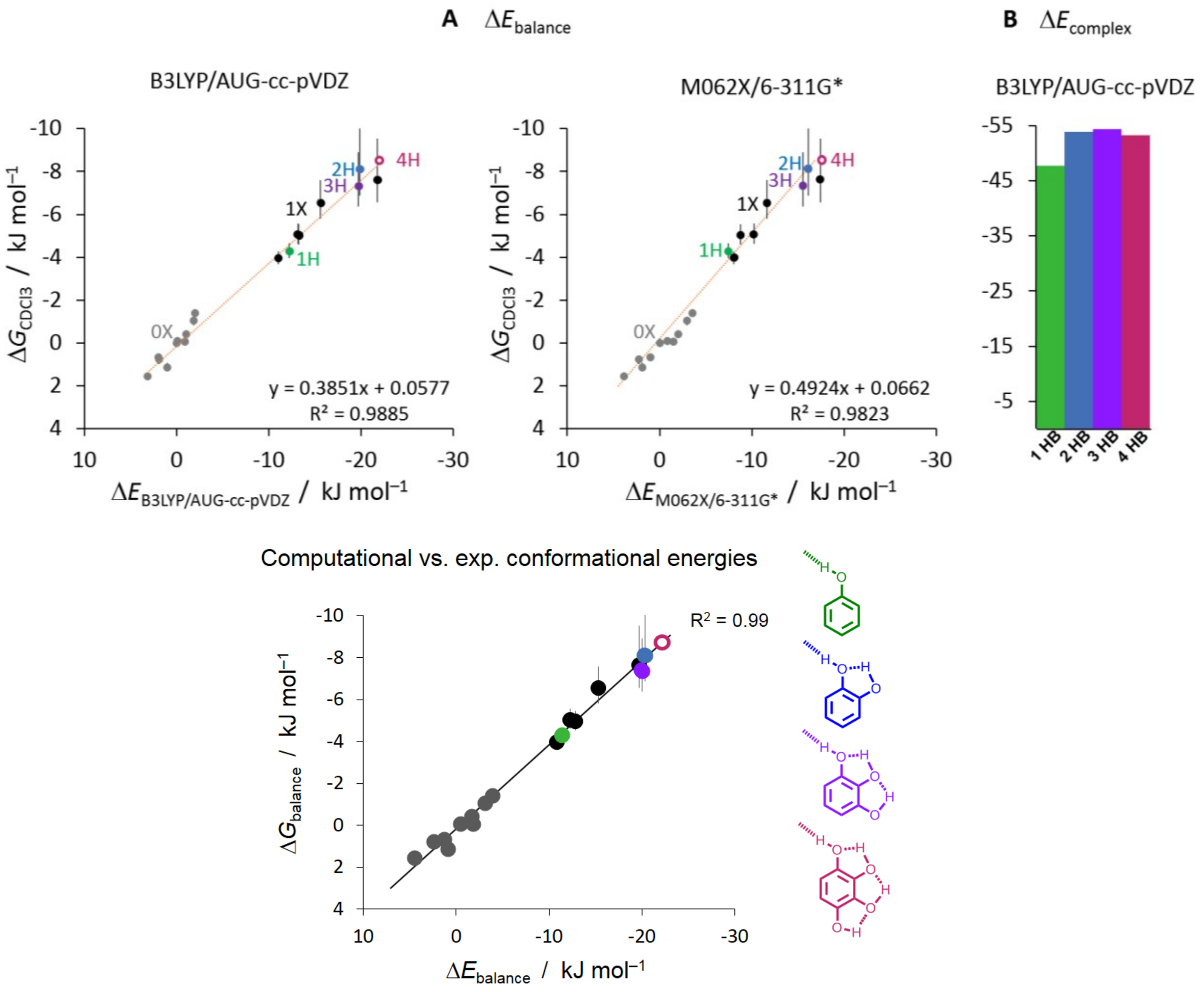

Figure S18. (A) Correlations of experimental conformational free energies of all of the molecular balances shown in Figure 2 (ΔG) against calculated energies (ΔE_e). The hollow pink circle is the calculated prediction for the tetrahydroxybenzene derivative. (B) Key data similar to that in Figure 3 of the main text, but calculated using B3LYP/AUG-cc-pVDZ.

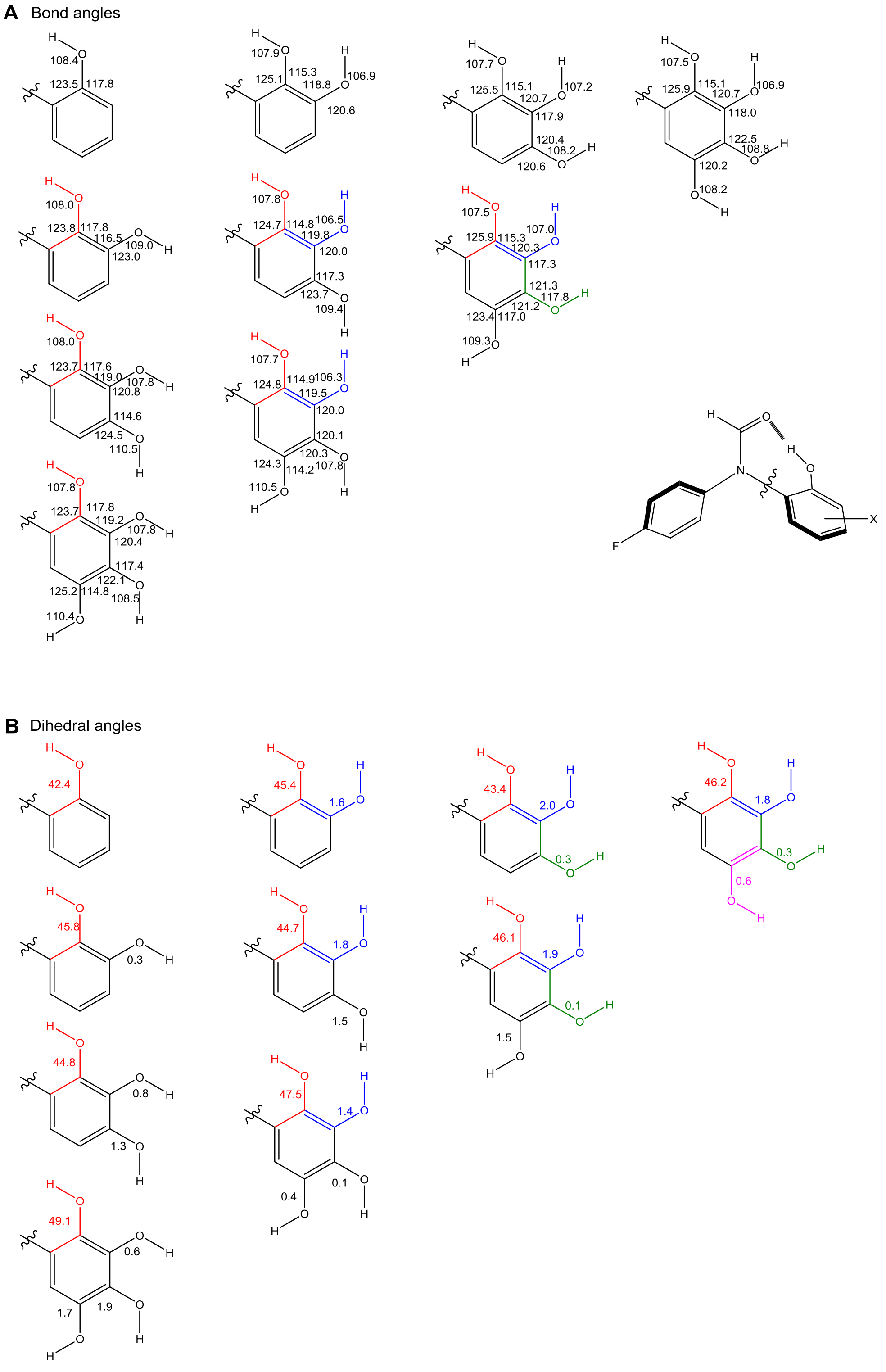

Figure S19. (A) Calculated bond angles and dihedral angles for balances **1H**, **2H**, **3H** and **4H** in the global minimum conformations (top row) and the indicated local minimum conformations in which the H-bond chain was broken by flipping the OH groups (B3LYP/6-311G*). No relationship between bond angles and dihedral angles and the strength of the terminal H-bond is revealed.

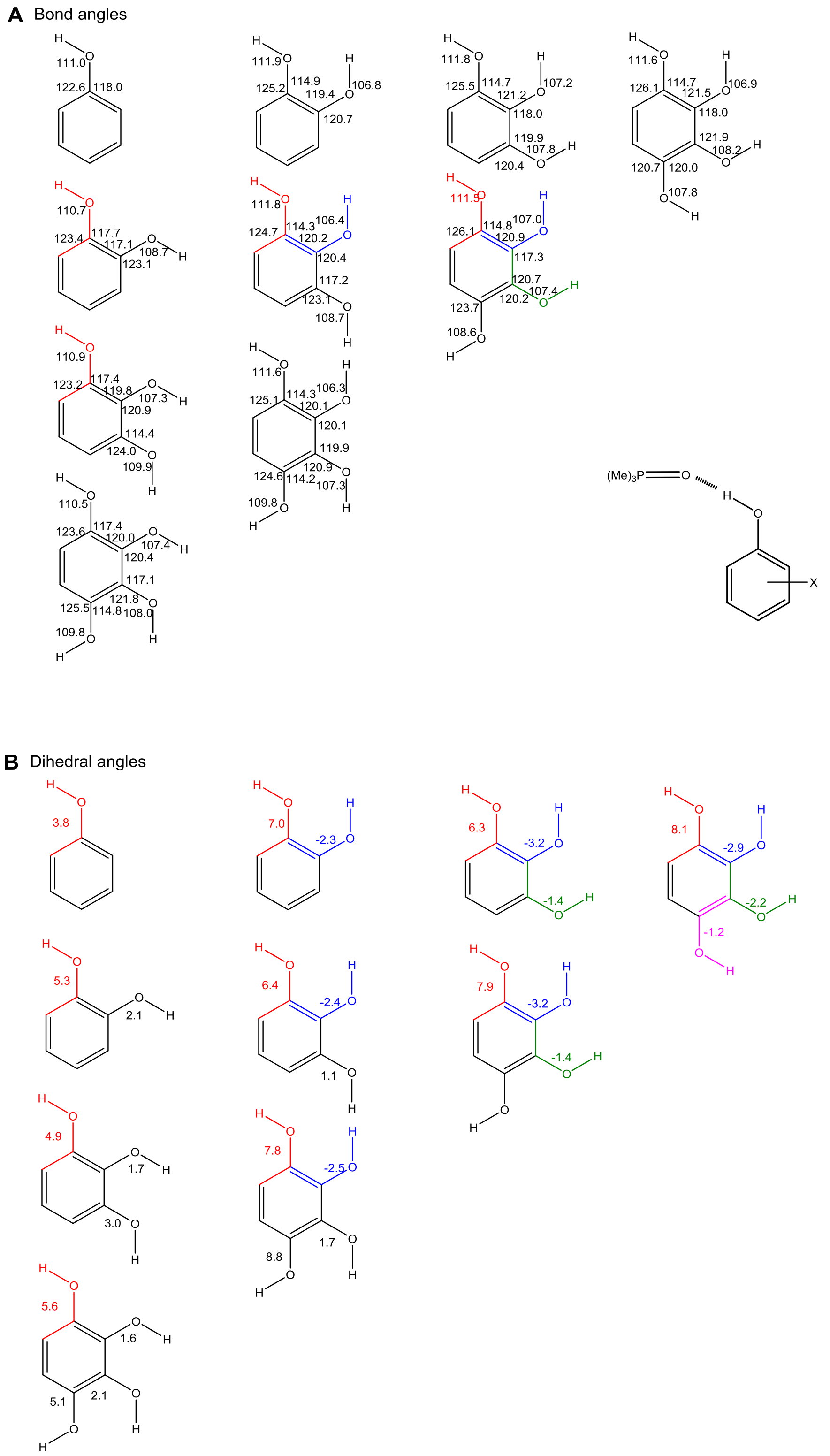

Figure S20. (A) Calculated bond angles and dihedral angles for the phosphine oxide-phenol derivative complexes shown inset in the global minimum conformations (top row) and the indicated local minimum conformations in which the H-bond chain was broken by flipping the OH groups (B3LYP/6-311G*). No relationship between bond angles and dihedral angles and the strength of the terminal H-bond is revealed.

6. Synthesis and Compound Characterization

General

NMR spectra were recorded using the following instruments: Bruker Ultrashield 400 MHz, heteronuclear; Bruker Ascend 500 MHz equipped with a DCH cryoprobe and ^{13}C optimization; Bruker Ascend 500 MHz with prodigy cryoprobe, heteronuclear; Bruker Ultrashield 600 MHz with TCI cryoprobe. In all cases ^{1}H and ^{13}C spectra were referenced to deuterated solvent peaks as reported in *J. Org. Chem*. 1997, 62, 7512 and coupling constants are quoted to the nearest 0.1 Hz.

High-resolution mass spectroscopy was carried out using a Bruker micrOTOF II. Logan Mackay (University of Edinburgh) is thanked for this service.

Unless otherwise specified, the term "ether" relates to diethyl ether, "petroleum ether" relates to the 40-60 °C boiling point range grade and "dppf" refers to 1,1'-bis(diphenylphosphino)ferrocene. Where 'anhydrous' solvents (THF, toluene, DCM, ether) are specified these were purified using a "Glass Contour" brand solvent purification system (SPS). 'Anhydrous' DMF was purchased as such from Sigma-Aldrich or Acros organics and used as supplied. Glassware was dried overnight in an oven (130-140 °C). Reactions were carried out in septum-sealed vessels under an atmosphere of nitrogen. Solvents for other reactions, purification, etc. were purchased from commercial suppliers and used as supplied unless stated otherwise. Degassing was carried out by sonication whilst bubbling nitrogen through the solvent for 30 minutes. All reagents were purchased from commercial suppliers and used as supplied unless specified otherwise. Flash chromatography was carried out using Geduran silica gel 60. Analytical TLC was carried out using Merck silica gel 60 F254 plates. Visualization was carried out using UV light and/or "Goofy's dip", an aqueous solution containing molybdic acid, cerium sulfate and sulfuric acid.

Synthesis of Compound 1H: *N*-(4-fluorophenyl)-*N*-(2-hydroxyphenyl)formamide

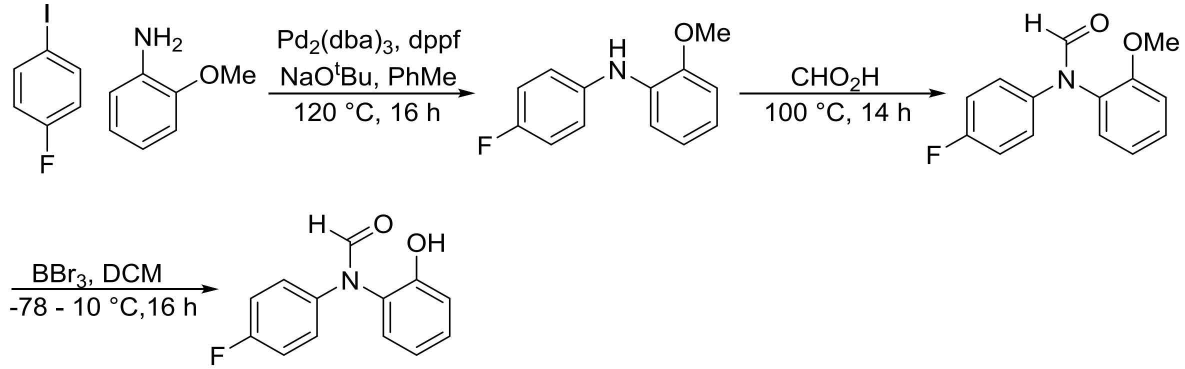

***N*-(4-fluorophenyl)-2-methoxy-aniline**

2-Methoxyaniline (1 equiv, 3.25 mmol, 400 mg, 370 μL), 4-fluoroiodobenzene (1 equiv, 3.25 mmol, 721 mg, 375 μL), $Pd_2(dba)_3$ (0.02 equiv, 0.065 mmol, 59.5 mg), dppf (0.03 equiv, 0.10 mmol, 54 mg) and sodium *tert*-butoxide (1.5 equiv, 4.87mmol, 468 mg) were combined in a 5 mL microwave tube and purged with nitrogen. Degassed toluene (4.6 mL) was added and the reaction heated to 120 °C for 16 h. The reaction mixture was filtered through kieselguhr eluting with ethyl acetate (40 mL) and washed with saturated aqueous sodium bicarbonate (40 mL) and then brine (30 mL). Organics were dried over sodium sulfate and solvents were removed under reduced pressure. The residue was purified by flash chromatography (4% ethyl acetate in petroleum ether) to yield *N*-(4-fluorophenyl)-2-methoxy-aniline (581 mg, 82%).

^{1}H NMR (500 MHz, $CDCl_3$) δ 7.15 – 7.08 (m, 3H), 7.02 – 6.95 (m, 2H), 6.91 – 6.81 (m, 3H), 6.04 (s, 1H), 3.90 (s, 3H).

^{13}C NMR (126 MHz, $CDCl_3$) δ 158.3 (d, J = 240.2 Hz), 148.0 (s), 138.7 (d, J = 2.4 Hz), 134.0 (s), 121.4 (d, J = 7.8 Hz), 121.0 (s), 119.7 (s), 116.0 (d, J = 22.4 Hz), 113.8 (s), 110.6 (s), 55.7 (s).

^{19}F NMR (471 MHz, $CDCl_3$) δ -121.88 – -122.00 (m).

HRMS (ESI): obtained m/z 218.0977 ($M+H^+$). Expected 218.0976.

^{1}H NMR

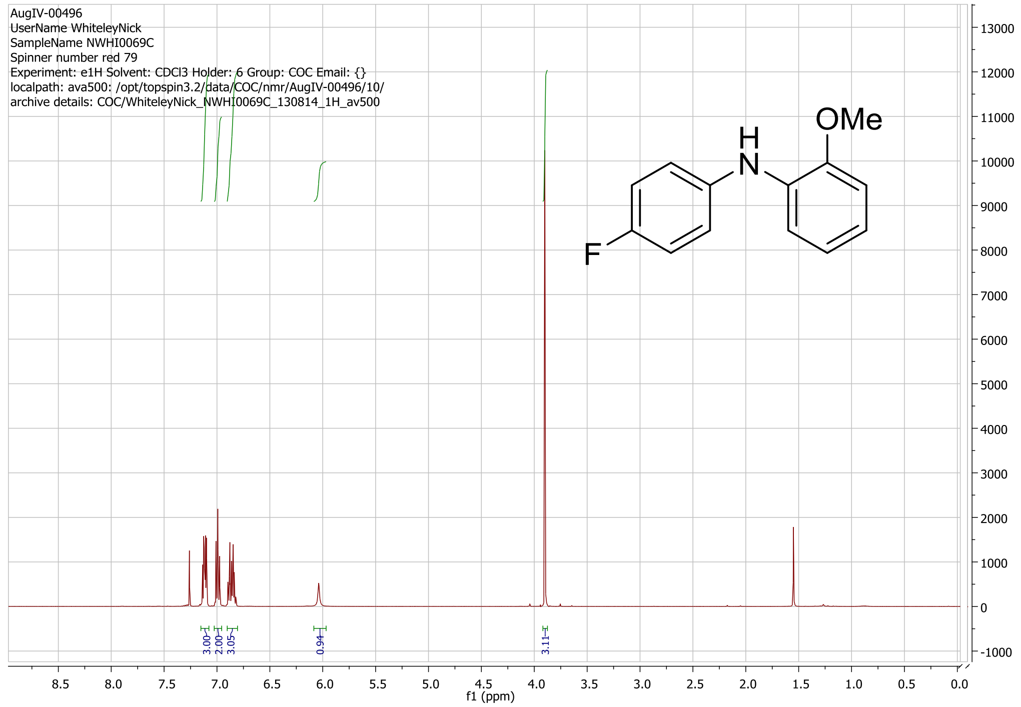

^{13}C NMR

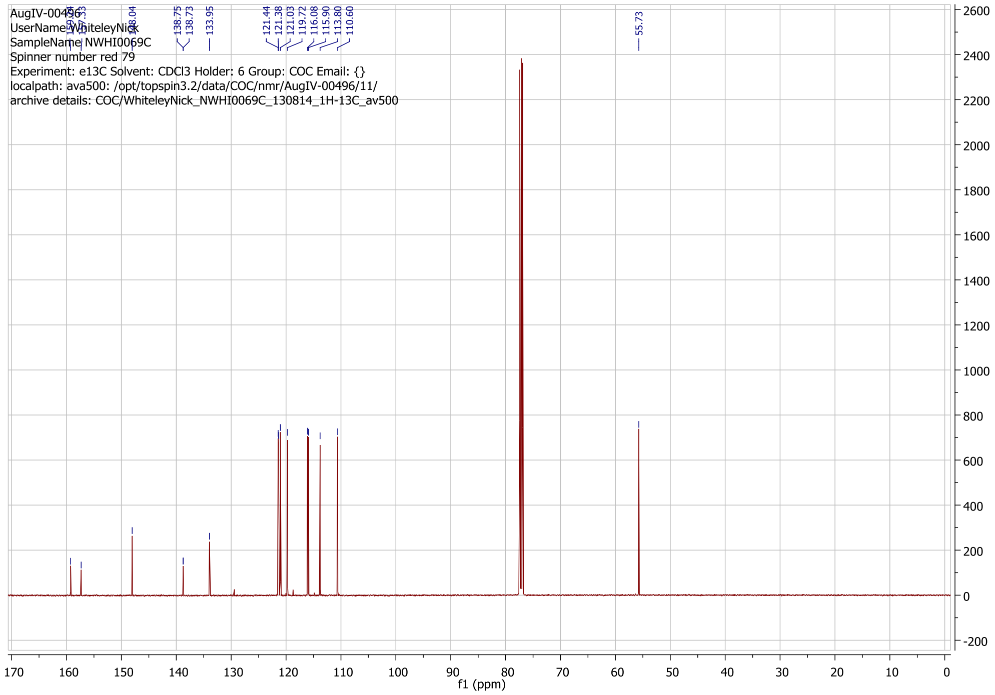

N-(4-fluorophenyl)-N-2-methoxyphenyl-formamide

N-(4-fluorophenyl)-2-methoxy-aniline (1 equiv, 3.64 mmol, 575 mg) was dissolved in formic acid (8.8 mL) and refluxed for 14 h. Solvent was removed under reduced pressure, the residue dissolved in ethyl acetate (40 mL) and washed with saturated aqueous sodium bicarbonate (40 mL) followed by brine (30 mL). Organics were dried over sodium sulfate and solvents removed under reduced pressure. The residue was purified by flash chromatography (15-25% ethyl acetate in petroleum ether) to yield *N*-(4-fluorophenyl)-*N*-2-methoxyphenyl-formamide (536 mg, 83%).

^{1}H NMR (500 MHz, $CDCl_3$) δ 8.63 (s, 0.4H, minor conformer), 8.37 (s, 0.6H, major conformer), 7.40 – 7.33 (m, 1H), 7.32 – 7.27 (m, 1H), 7.23 – 7.18 (m, 1H), 7.16 – 7.11 (m, 1H), 7.07 – 6.98 (m, 4H), 3.79 (s, 1.8H, major conformer), 3.79 (s, 1.2H, minor conformer).

^{13}C NMR (126 MHz, $CDCl_3$) δ 163.0 (s, major conformer), 161.9 (s, minor conformer), 161.1 (d, J = 246.2 Hz, minor conformer), 160.6 (d, J = 245.5 Hz, major conformer), 155.7 (s, major conformer), 155.20 (s, minor conformer), 138.2 (d, J = 3.0 Hz, minor conformer), 136.4 (d, J = 3.0 Hz, major conformer), 130.0 (s), 129.9 (s), 129.9 (s), 129.8 (s), 129.7 (s), 127.9 (s), 126.6 (d, J = 8.2 Hz, major conformer), 125.3 (d, J = 8.4 Hz, minor conformer), 121.3 (s), 116.3 (d, J = 22.8 Hz, minor conformer), 115.7 (d, J = 22.6 Hz, major conformer), 112.7 (s, minor conformer), 112.5 (s, major conformer), 55.9 (s, minor conformer), 55.9 (s, major conformer). One conformer peak (from methoxy-substituted ring) not observed.

^{19}F NMR (471 MHz, $CDCl_3$) δ -115.85 – -115.92 (m, 0.4F), -116.05 – -116.13 (m, 0.6F). Two conformers.

HRMS (ESI): obtained m/z 246.0923 ($M+H^+$). Expected 246.0925.

^{1}H NMR

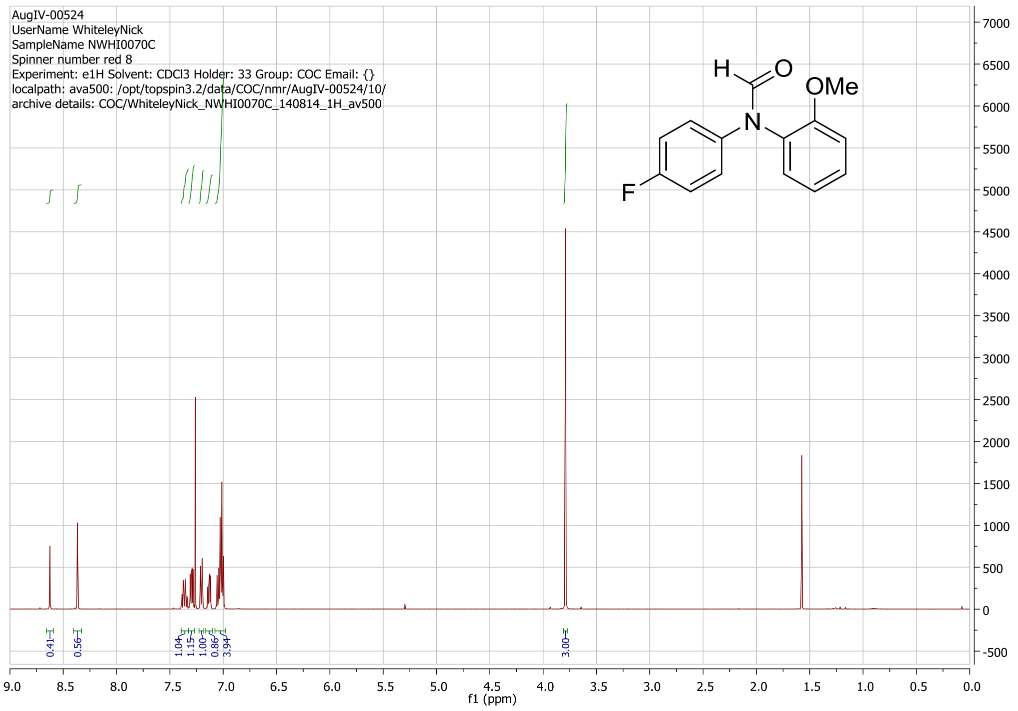

^{13}C NMR

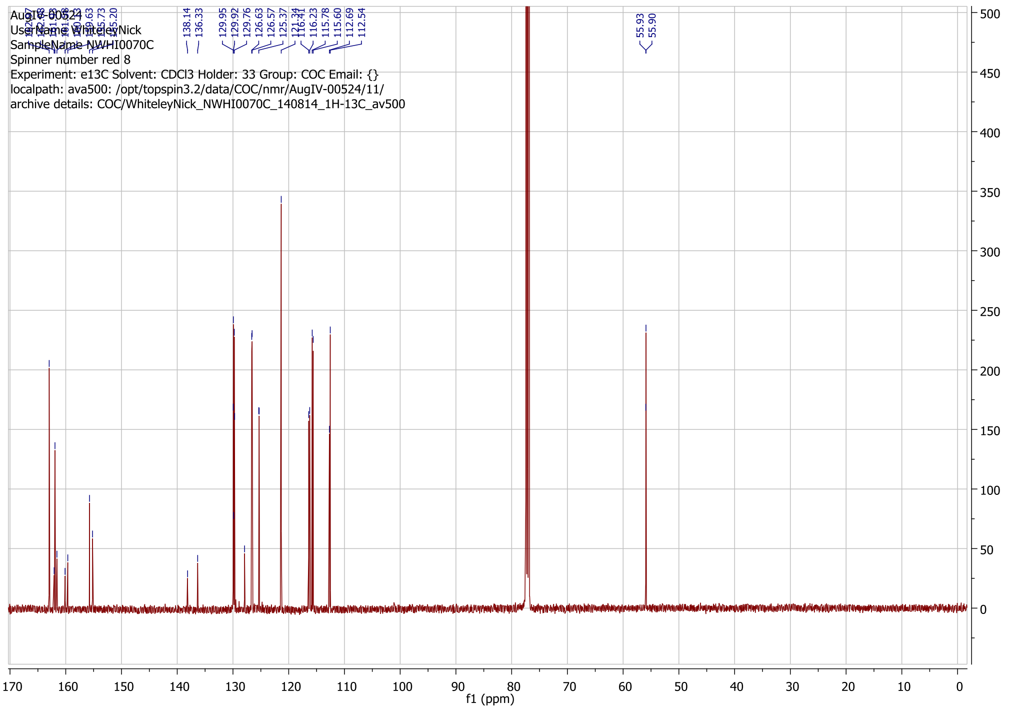

Compound 1H: *N*-(4-fluorophenyl)-*N*-(2-hydroxyphenyl)formamide

N-(4-fluorophenyl)-*N*-(2-methoxyphenyl)formamide (1 equiv, 2.19 mmol, 536 mg) was dissolved in anhydrous DCM (11 mL) and cooled to -78 °C. A solution of BBr_3 in DCM (6 equiv, 13.1 mmol, 33% v/v, 4.2 mL) was added dropwise and the mixture stirred for 20 h allowing gradual warming to 15 °C. The reaction mixture was added dropwise to water (75 mL) at 0 °C. Additional DCM (100 mL) was added and organics separated. The aqueous layer was then re-extracted with DCM (2 x 100 mL). Combined organics were washed with brine (100 mL), dried over sodium sulfate and solvents removed under reduced pressure. The residue was purified by flash chromatography (20-40% ethyl acetate in petroleum ether) to yield crude product. This was further purified by the addition of pentane (10 mL) and a few drops of DCM. A white suspension was decanted from a black oil and solvent was removed under reduced pressure. The residue obtained was re-purified by flash chromatography (20% ethyl acetate in hexane) to yield *N*-(4-fluorophenyl)-*N*-(2-hydroxyphenyl)formamide (150 mg, 30%) as a film.

^{1}H NMR (400 MHz, $CDCl_3$) δ 8.55 (s, 0.7 H, major conformer), 8.41 (s, 0.3 H, minor conformer), 7.50 – 6.56 (overlapping multiplets, 8.7 H), 5.86 (s, 0.3 H, minor conformer).

^{13}C NMR (151 MHz, $CDCl_3$) δ 162.5 (s, major conformer), 161.9 (d, J = 248.7 Hz, major conformer), 152.3 (s, minor conformer), 150.9 (s, major conformer), 137.3 (d, J = 3.4 Hz, major conformer), 130.2 (s, presumed minor conformer), 129.6 (s, presumed minor conformer), 129.0 (s, major conformer), 128.8 (s, major conformer), 127.5 (d, J = 8.6 Hz, major conformer), 126.5 (s, major conformer), 125.9 (d, J = 8.0 Hz, minor conformer), 121.5 (s, major conformer), 120.5 (s, major conformer), 117.6 (s, minor conformer), 116.9 (d, J = 23.0 Hz, major conformer), 115.9 (d, J = 22.5 Hz, minor conformer). Not all minor conformer peaks observed above baseline.

^{19}F NMR (376 MHz, $CDCl_3$) δ -112.99 (major conformer), -115.17 (minor conformer).

HRMS (ESI): obtained m/z 232.0778 ($M+H^+$). Expected 232.0768.

1D NMRS (full 2D and conformer assignment are presented in section S7)

^{1}H NMR

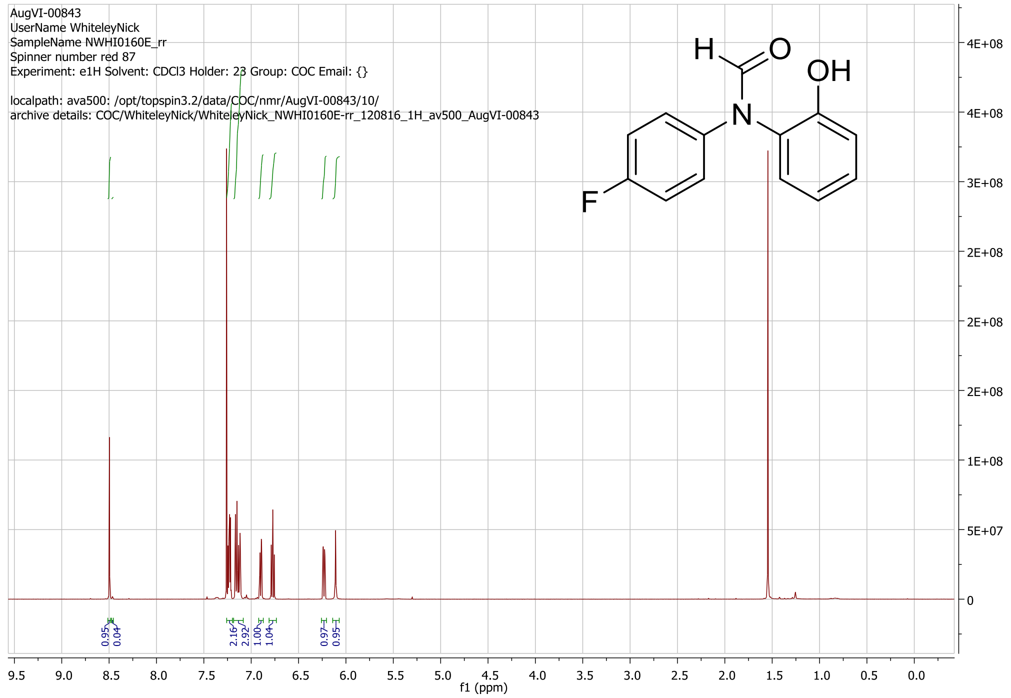

^{13}C NMR

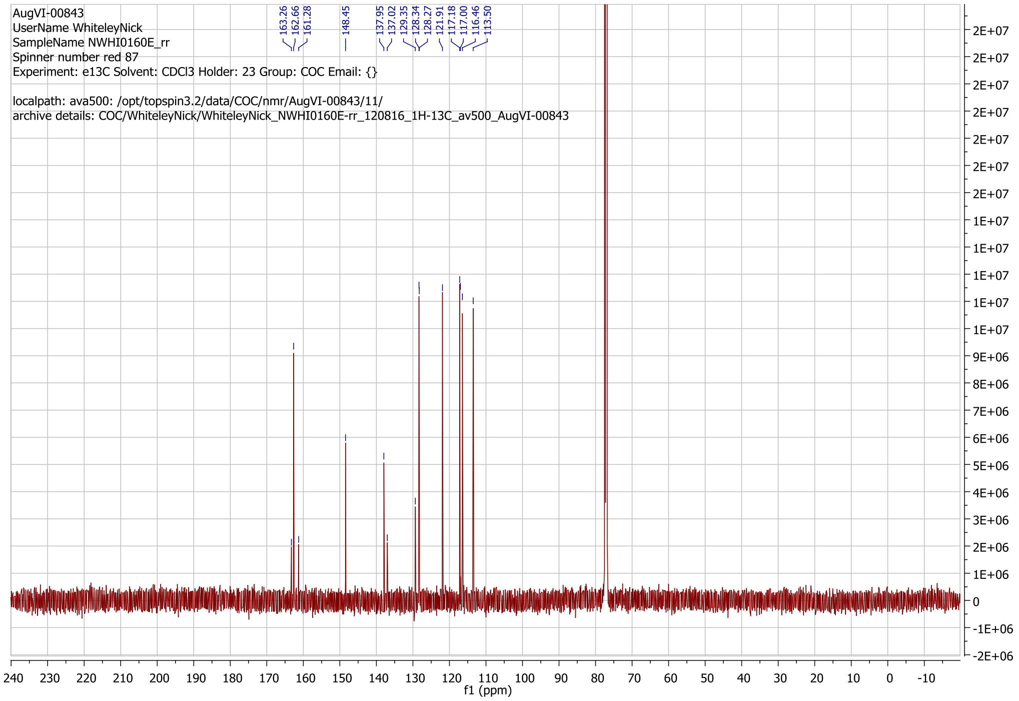

Synthesis of Compound 2H: *N*-(2,3-dihydroxyphenyl)-*N*-(4-fluorophenyl)formamide

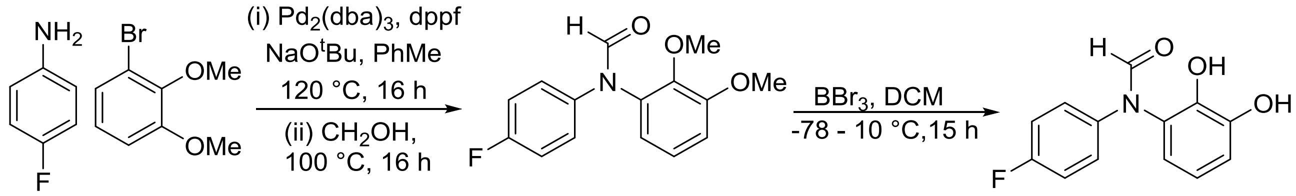

***N*-(2,3-dimethoxyphenyl)-*N*-(4-fluorophenyl)formamide**

2,3-Dimethoxyaniline (1 equiv, 4.90 mmol, 749 mg, 0.66 mL), 4-fluoroiodobenzene (1 equiv, 4.90 mmol, 1.07 g, 0.57 mL), $Pd_2(dba)_3$ (0.02 equiv, 0.12 mmol, 112 mg), dppf (0.04 equiv, 0.18 mmol, 102 mg), and sodium *tert*-butoxide (1.5 equiv, 7.34 mmol, 825 mg) were combined and the vessel sealed under nitrogen. Anhydrous toluene (7 mL) was added and the reaction heated to 120 °C for 48 h. The reaction mixture was then washed with saturated aqueous sodium bicarbonate (15 mL). The aqueous layer then re-extracted with toluene (3 x 5 mL). The organic layers were then combined, dried over magnesium sulfate and solvent removed under reduced pressure. The residue was taken up in formic acid (20 mL) and heated to reflux under a nitrogen atmosphere for 16 h. Solvent was removed under reduced pressure, the residue dissolved in ethyl acetate (100 mL) and washed with saturated aqueous sodium bicarbonate (2 x 50 mL) and then brine (2 x 50 mL). Organics were dried over magnesium sulfate and solvents removed under reduced pressure. The residue was purified by flash chromatography (0-20% ethyl acetate in petroleum ether) to yield *N*-(2,3-dimethoxyphenyl)-*N*-(4-fluorophenyl)formamide (837 mg, 58% over 2 steps).

^{1}H NMR (500 MHz, $CDCl_3$) δ 8.66 (s, 0.4H, minor conformer), 8.44 (s, 0.6H, major conformer), 7.33 – 7.28 (m, 1H), 7.17 – 7.01 (m, 4H), 6.95 (apparent d, J = 1.4 Hz, 0.6H, major conformer), 6.94 (apparent d, J = 1.4 Hz, 0.4H, minor conformer) 6.83 (dd, J = 8.0, 1.4 Hz, 0.4H, minor conformer), 6.79 (dd, J = 8.0, 1.5 Hz, 0.6H, major conformer), 3.90 (s, 1.6H, major conformer), 3.88 (s, 1.4H, minor conformer), 3.75 (s, 1.4H, minor conformer), 3.66 (s, 1.6H, major conformer).

^{13}C NMR (126 MHz, $CDCl_3$) δ 162.6 (s, major conformer), 162.1 (s, minor conformer), 161.2 (d, J = 246.2 Hz, minor conformer), 160.7 (d, J = 246.0 Hz, major conformer), 154.1 (s, major conformer), 154.1 (s, minor conformer), 145.5 (s, minor conformer), 145.4 (s, major conformer), 138.1 (d, J = 3.0 Hz, minor conformer), 136.4 (d, J = 3.0 Hz, major conformer), 135.0 (s, major conformer), 132.9 (s, minor conformer), 126.8 (d, J = 8.3 Hz, major conformer), 125.4 (d, J = 8.4 Hz, minor conformer), 124.4 (s, major conformer), 124.3 (s, minor conformer), 121.1 (s, minor conformer), 120.6 (s, major conformer), 116.4 (d, J = 22.8 Hz, minor conformer), 115.8

(d, J = 22.7 Hz, major conformer), 112.8 (s, minor conformer), 112.4 (s, major conformer), 60.9 (s, major conformer), 60.6 (s, minor conformer), 56.2 (s, major conformer), 56.1 (s, minor conformer).

^{19}F NMR (471 MHz, $CDCl_3$) δ -115.59 – -115.65 (m, 0.4F), -115.68 – -115.75 (m, 0.6F). Two conformers.

HRMS (ESI): obtained m/z 276.1039 (M+H). Expected 276.1030.

^{1}H NMR

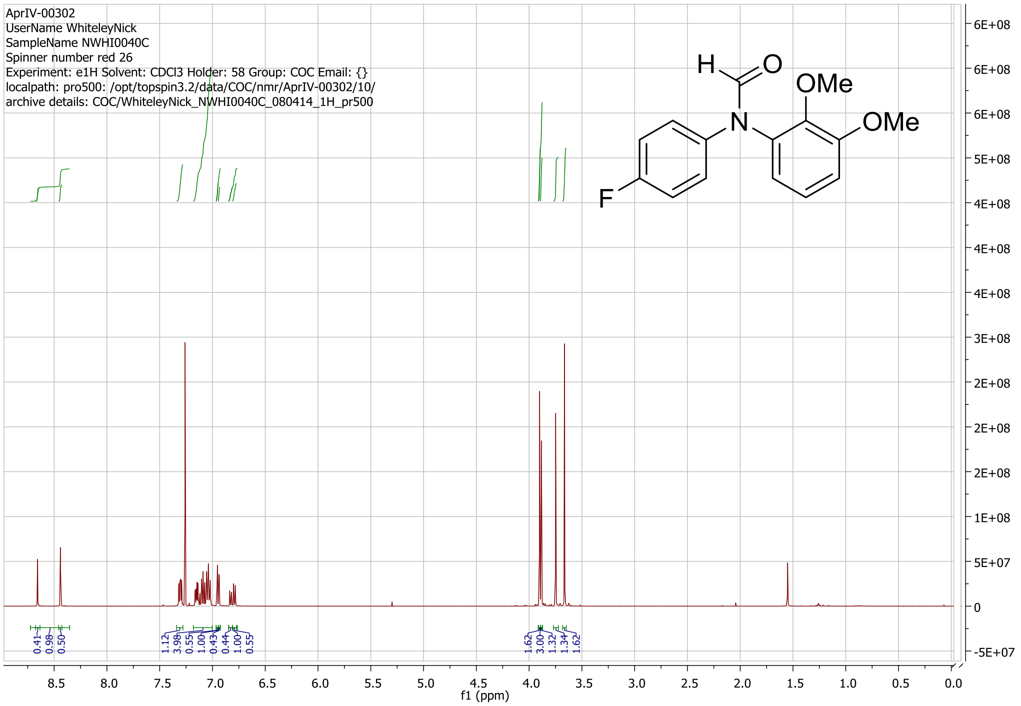

^{13}C NMR

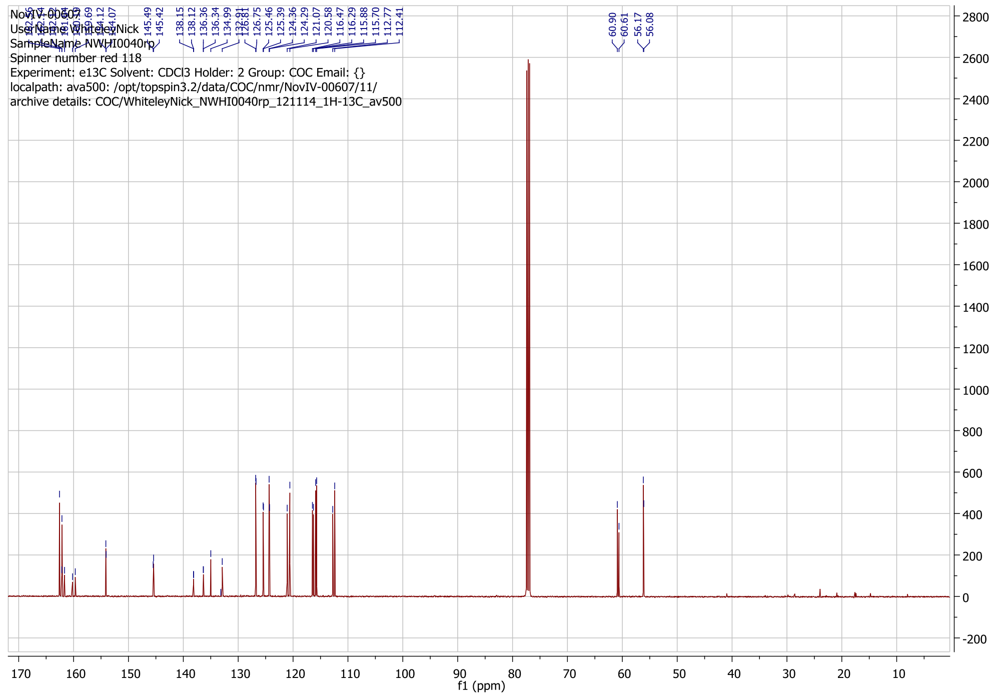
NovIV-00607
UserName WhiteleyNick
SampleName NWHI0040rp
Spinner number red 118
Experiment: e13C Solvent: CDCl3 Holder: 2 Group: COC Email: {}
localpath: ava500: /opt/topspin3.2/data/COC/nmr/NovIV-00607/11/
archive details: COC/WhiteleyNick_NWHI0040rp_121114_1H-13C_av500
159.69
154.12
154.07
145.49
145.42
138.15
138.12
136.36
136.34
134.99
126.75
125.46
125.39
124.36
124.29
121.07
120.58
116.47
116.29
115.88
115.70
112.77
112.41
60.90
60.61
56.17
56.08
f1 (ppm)

Compound 2H: *N*-(2,3-dihydroxyphenyl)-*N*-(4-fluorophenyl)formamide

N-(2,3-dimethoxyphenyl)-*N*-(4-fluorophenyl)formamide (1 equiv, 1.37 mmol, 376 mg) was dissolved in anhydrous DCM (6.8 mL) and cooled to -78 °C. A solution of BBr_3 in DCM (11 equiv, 15 mmol, 30% v/v, 6.8 mL) was added dropwise and the reaction allowed to warm gradually to 10 °C over a period of 15 h. The reaction mixture was added dropwise to water at 0 °C, and the reaction vessel rinsed with additional DCM (10 mL). The mixture obtained was extracted with DCM (50 mL) and the aqueous layer re-extracted with chloroform (3 x 50 mL). Combined organics were washed with brine (50 mL), dried over sodium sulfate and solvents removed under reduced pressure. The residue was triturated from DCM to yield *N*-(2,3-dihydroxyphenyl)-*N*-(4-fluorophenyl)formamide in quantitative yield (333 mg).

^{1}H NMR (601 MHz, $CDCl_3$) δ 8.52 (s, 0.9H, major conformer), 8.48 (s, 0.1H, minor conformer), 7.28 – 7.22 (m, 2H), 7.20 – 7.15 (m, 2H), 7.14 (s, 1H), 6.92 (dd, *J* = 8.0, 1.0 Hz, 1H), 6.80 (apparent t, *J* = 8.1 Hz, 1H), 6.26 (dd, *J* = 8.3, 1.3 Hz, 1H), 6.14 (s, 1H). Minor conformer peaks only sufficiently well-defined to be reported for the formyl proton.

^{13}C NMR (126 MHz, $CDCl_3$) δ 162.7 (s), 162.3 (d, *J* = 250.7 Hz), 148.4 (s), 138.0(s), 137.0 (d, *J* = 3.3 Hz), 129.3(s), 128.3 (d, *J* = 8.8 Hz), 121.9(s), 117.1(d, *J* = 23.0 Hz), 116.5(s), 113.5(s). Major conformer observed exclusively.

^{19}F NMR (376 MHz, $CDCl_3$) δ -112.33 (major conformer), -115.01 (minor conformer).

HRMS (ESI): Obtained m/z 248.0718 ($M+H^+$). Expected 248.0717.

1D NMRs (full 2D assignments are given in the Conformer Assignment section below)

^{1}H NMR

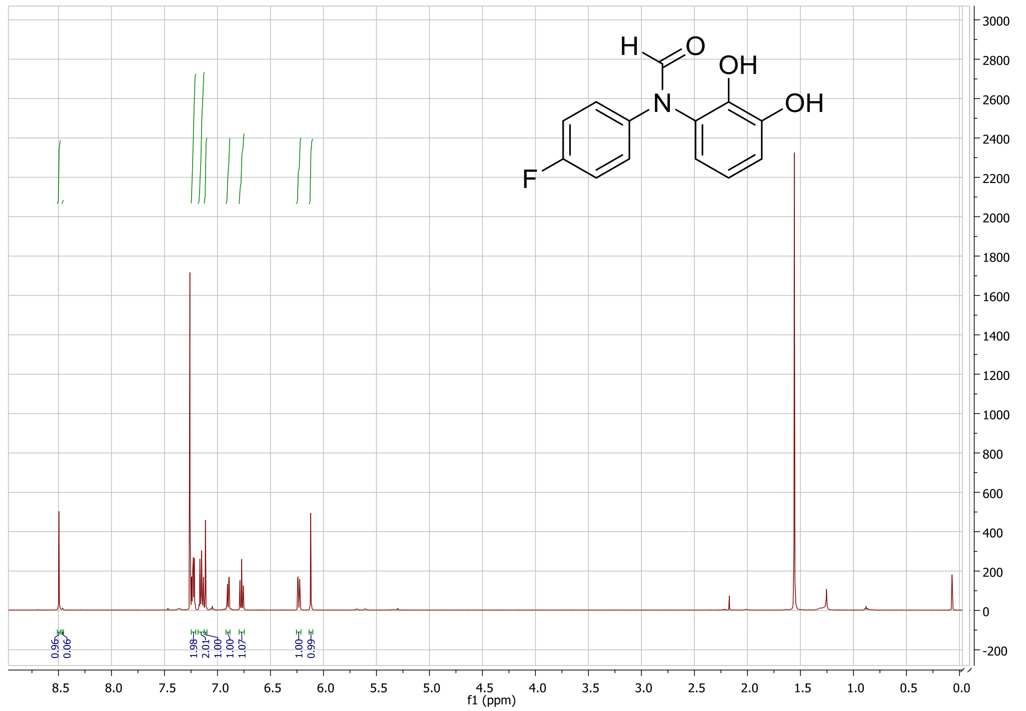

^{19}F NMRs (repeats showing determination of extreme conformer integrals)

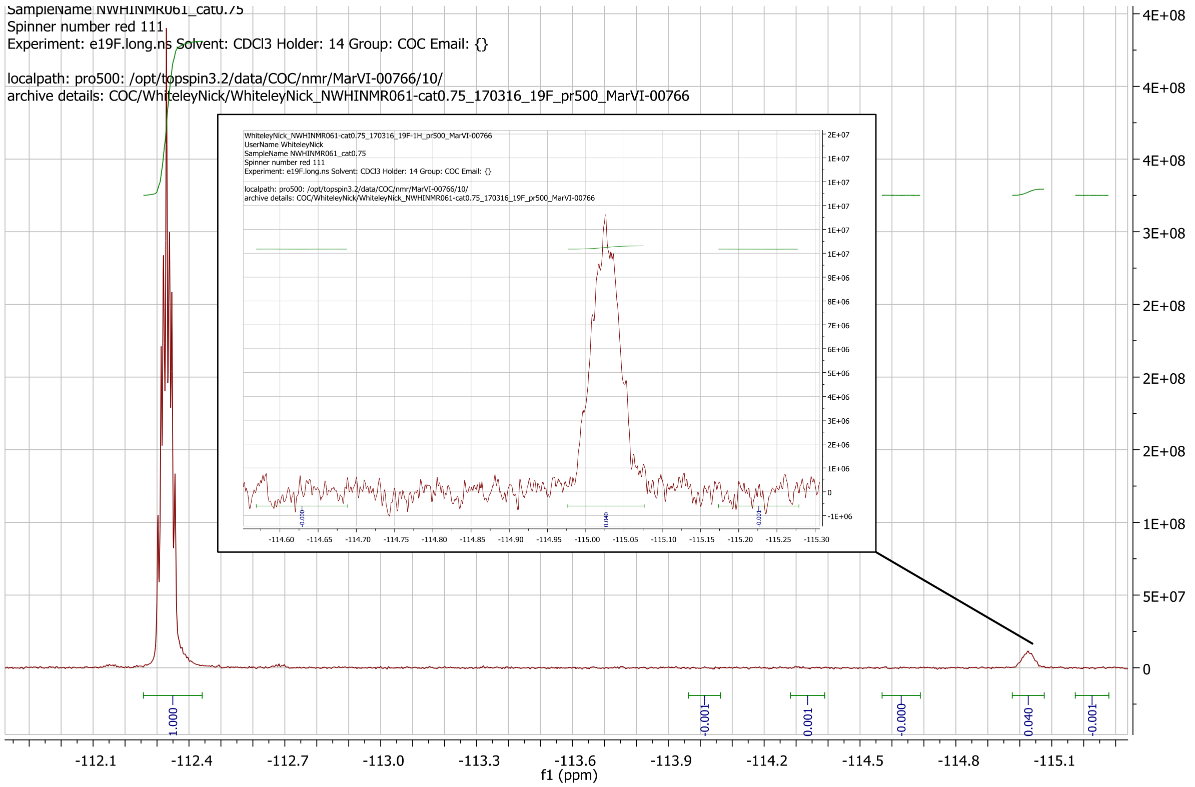

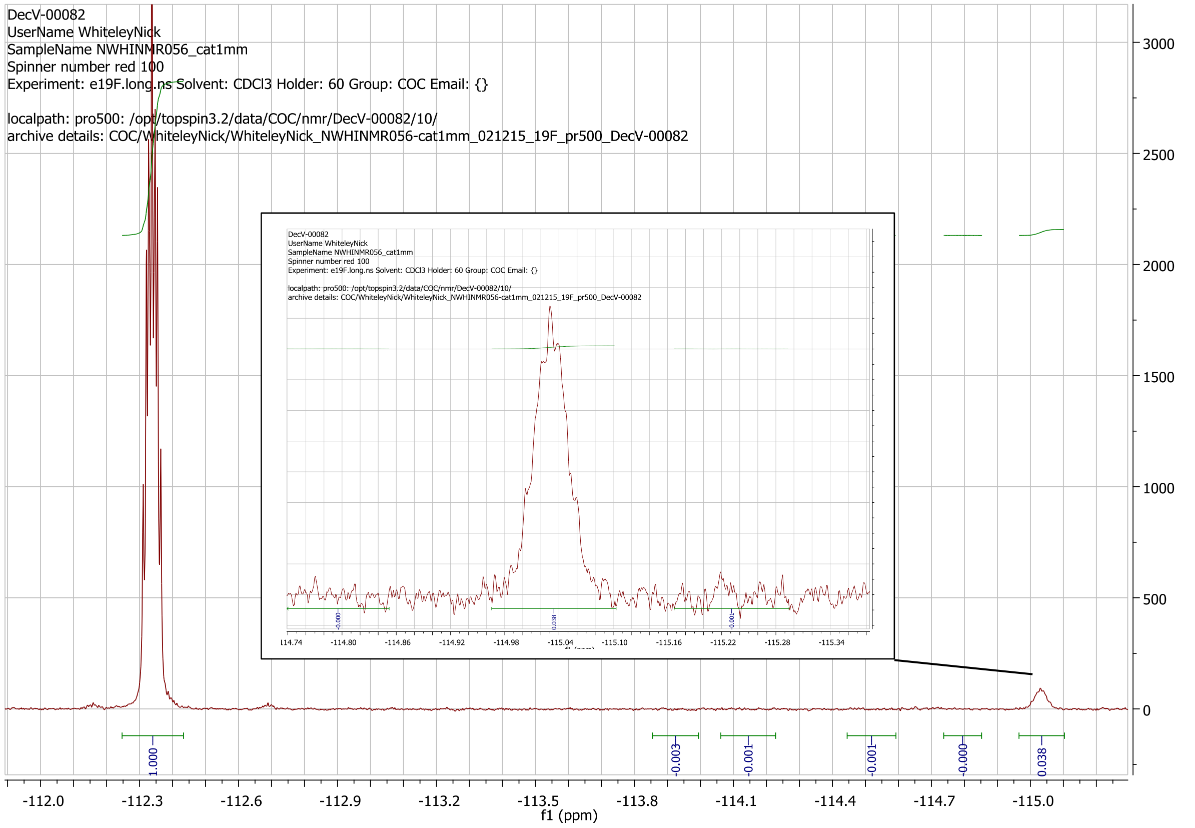

^{13}C NMR

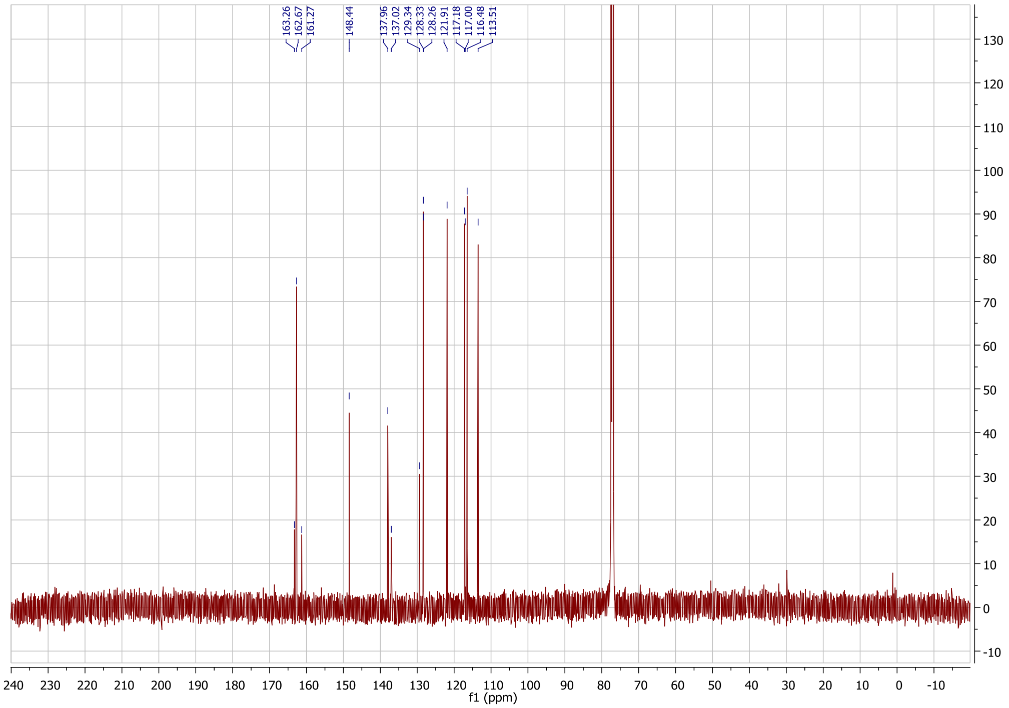
163.26
162.67
161.27
148.44
137.96
137.02
129.34
128.33
128.26
121.91
117.18
117.00
116.48
113.51
f1 (ppm)

Synthesis of Compound 3H: *N*-(4-fluorophenyl)-*N*-(2,3,4-trihydroxyphenyl)formamide

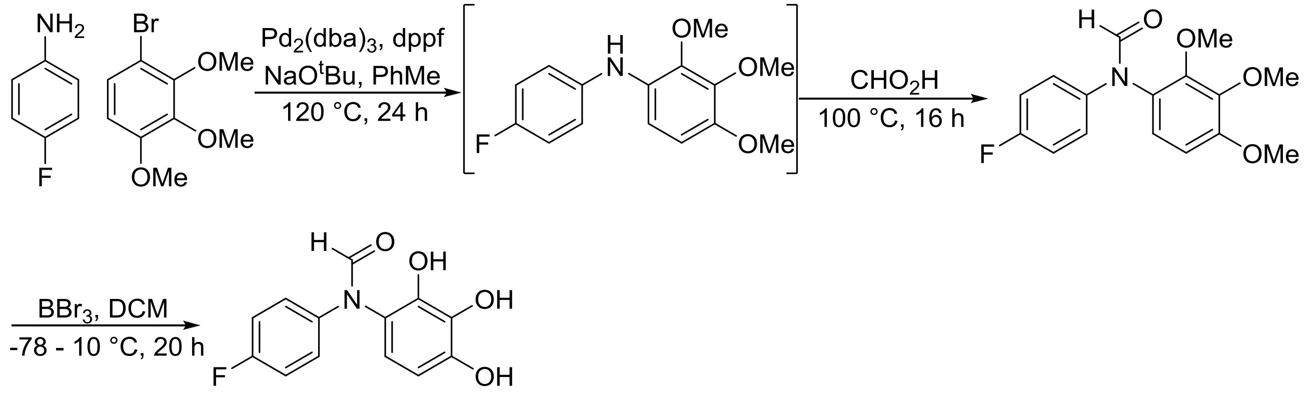

***N*-(4-fluorophenyl)-*N*-(2,3,4-trimethoxyphenyl)formamide**

2,3,4-(Trimethoxy)bromobenzene (1 equiv, 3.63 mmol, 897 mg), 4-fluoroaniline (1.6 equiv, 5.81 mmol, 645 mg), $Pd_2(dba)_3$ (0.019 equiv, 0.067 mmol, 61.5 mg), dppf (0.028 equiv, 0.102 mmol, 56.3 mg) and sodium *tert*-butoxide (1.4 equiv, 5.08 mmol, 488 mg) were combined in a 5 mL microwave tube and purged with nitrogen. Degassed toluene (4.9 mL) was added and the reaction heated to 120 °C for 24 h. The reaction mixture was filtered through kieselguhr eluting with ethyl acetate (40 mL). This was then washed with saturated aqueous sodium bicarbonate (30 mL) and then brine (30 mL). Organics were dried over sodium sulfate and solvents removed under reduced pressure. The residue was purified by flash chromatography (4-6% ethyl acetate in petroleum ether to yield *N*-(4-fluorophenyl)-2,3,4-trimethoxy-aniline (650 mg, 65% at *circ*a 85% purity).

Crude *N*-(4-fluorophenyl)-2,3,4-trimethoxy-aniline (650 mg) from the previous reaction was then dissolved in formic acid (7.8 mL) and refluxed for 12 h. Solvent was removed under reduced pressure, the residue dissolved in ethyl acetate (25 mL) and washed with saturated aqueous sodium bicarbonate (25 mL) then brine (25 mL). Organics were dried over sodium sulfate and solvents removed under reduced pressure. The residue was purified by flash chromatography (20-30% ethyl acetate in petroleum ether) to yield *N*-(4-fluorophenyl)-*N*-(2,3,4-trimethoxyphenyl)formamide (636 mg, 58% over 2 steps).

^{1}H NMR (500 MHz, $CDCl_3$) δ 8.65 (s, 0.5H), 8.38 (s, 0.5H), 7.33 – 7.28 (m, 1H), 7.16 – 7.11 (m, 1H), 7.08 – 7.01 (m, 2H), 6.93 (overlapping doublets, *J* = 8.7 Hz, 1H), 6.70 (apparent t, *J* = 8.7 Hz, 1H), 3.89 (overlapping singlets, 6H), 3.75 (s, 1.5H), 3.68 (s, 1.5H).

^{13}C NMR (126 MHz, $CDCl_3$) δ 162.6 (s, major conformer), 162.4 (s, minor conformer), 161.1 (d, *J* = 247.0 Hz, minor conformer), 160.5 (d, *J* = 245.7 Hz, major conformer), 154.3 (s, major

conformer), 154.2 (s, minor conformer), 150.4 (s, minor conformer), 150.1 (s, major conformer), 143.4 (s, minor conformer), 143.3 (s, major conformer), 138.5 (d, *J* = 3.0 Hz, minor conformer), 136.7 (d, *J* = 3.0 Hz, major conformer), 127.8 (s, minor conformer), 126.3 (d, *J* = 8.2 Hz, major conformer) 125.7 (s, major conformer), 125.2 (d, *J* = 8.4 Hz, minor conformer), 123.8 (s, minor conformer), 123.6 (s, major conformer), 116.4 (d, *J* = 22.8 Hz, minor conformer), 115.7 (d, *J* = 22.6 Hz, major conformer), 107.3 (s, minor conformer), 107.1 (s, major conformer), 61.2 (s), 61.1 (s), 61.0 (s), 60.9 (s), 56.3 (s, major conformer), 56.3 (s, minor conformer).

^{19}F NMR (376 MHz, CD_3CN, proton decoupled) δ -118.09 (s, 0.5F), -118.38 (s, 0.5F). Two conformers.

HRMS (ESI): Obtained m/z 328.0954 (M+Na). Expected 328.0956.

^{1}H NMR

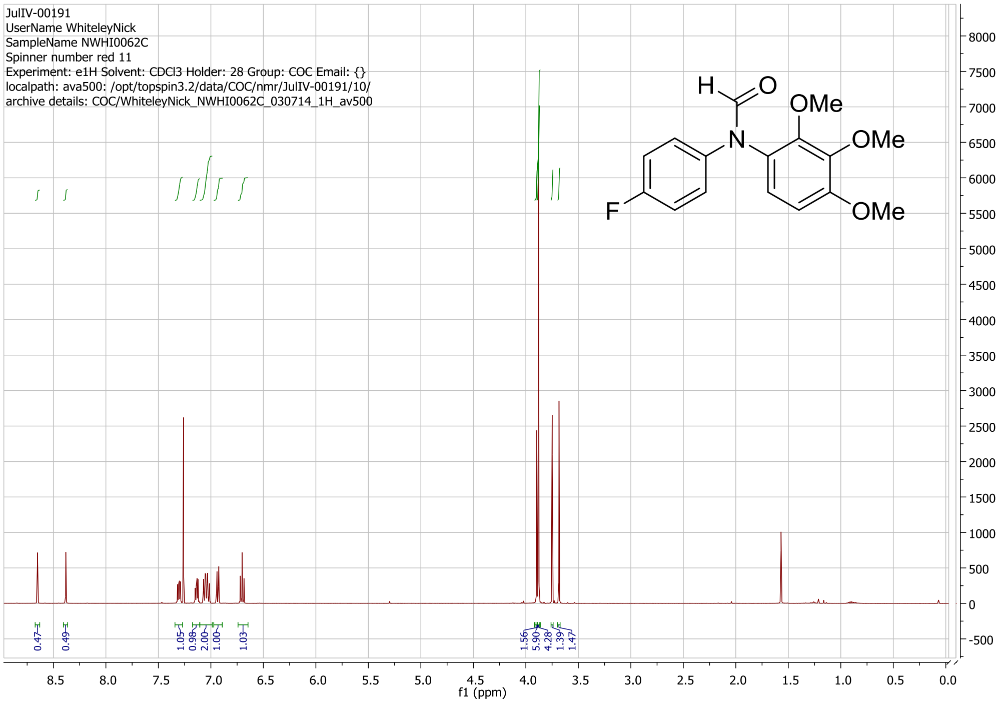

^{13}C NMR

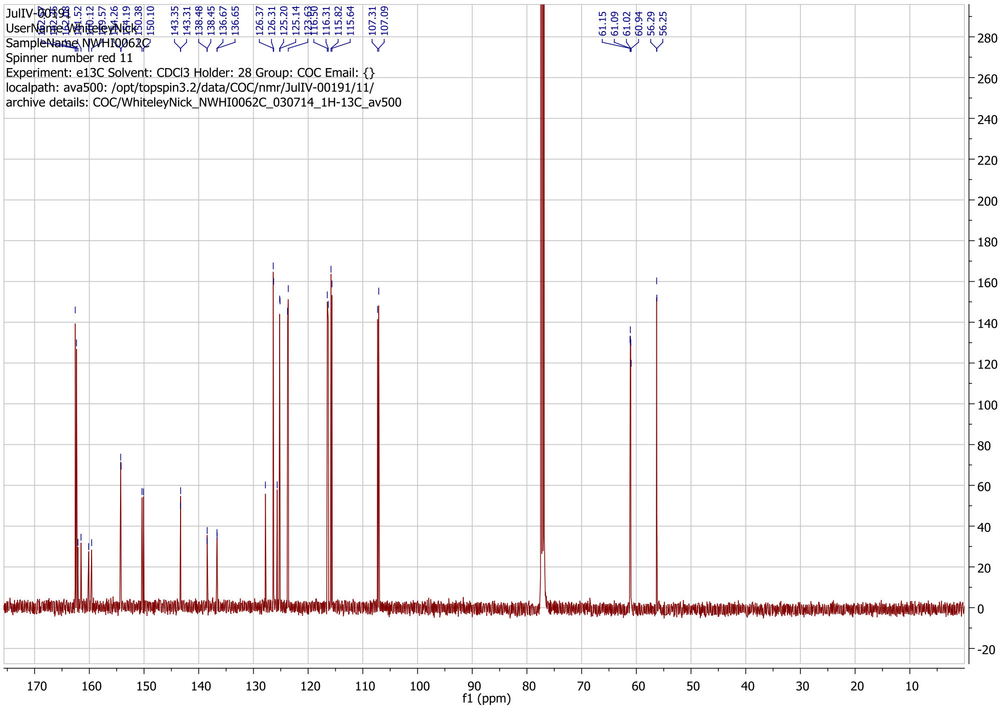

Compound 3H: *N*-(4-fluorophenyl)-*N*-(2,3,4-trihydroxyphenyl)formamide

N-(4-fluorophenyl)-*N*-(2,3,4-trimethoxyphenyl)formamide (1 equiv, 1.60 mmol, 490 mg) was dissolved in anhydours DCM (8 mL) and cooled to -78 °C. A solution of BBr_3 in DCM (11 equiv, 17.7 mmol, 33 % v/v, 5.7 mL) was added dropwise and the mixture stirred for 20 h allowing gradual warming to 15 °C. The reaction mixture was added dropwise to water (75 mL) at 0 °C. Additional DCM (100 mL) was added and organics separated. The aqueous layer was then re-extracted with DCM (2 x 100 mL). Combined organics were washed with brine (100 mL), dried over sodium sulfate and solvents removed under reduced pressure. The residue was purified by trituration from 1:1 pentane:DCM and dried overnight under vacuum to yield *N*-(4-fluorophenyl)-*N*-(2,3,4-trihydroxyphenyl)formamide (100 mg, 24%) as a white solid.

^{1}H NMR (500 MHz, CD_3CN) δ 8.60 (s, 0.5H), 8.30 (s, 0.5H), 7.39 – 7.32 (m, 1H), 7.26 – 7.18 (m, 1H), 7.16 – 7.03 (m, 2H), 6.76 (broad s, 3H), 6.66 (d, *J* = 8.7 Hz, 1H), 6.48 (d, *J* = 8.7 Hz, 1H), 6.45 (s, 1H).

^{1}H NMR (500 MHz, $CDCl_3$) δ 8.49 (s, 1H, major conformer), 8.43 (s, trace, minor conformer), 7.27 – 7.20 (m, 3H), 7.20 – 7.10 (m, 2H), 6.52 (d, *J* = 9.0 Hz, 1H), 6.21 (d, *J* = 9.0 Hz, 1H), 5.93 (broad s, 1H), 5.40 (broad s, 1H).

^{13}C NMR (126 MHz, CD_3CN) δ 162.9 (s), 162.3 (s), 160.8 (d, *J* = 243.4 Hz), 160.0 (d, *J* = 242.9 Hz), 145.6 (s), 145.1 (s), 142.8 (s), 141.7 (s), 138.5 (d, *J* = 2.8 Hz), 137.1 (d, *J* = 2.9 Hz), 133.6 (s, *J* = 32.0 Hz), 133.3 (s), 125.9 (d, *J* = 8.3 Hz), 125.2 (d, *J* = 8.6 Hz), 121.3 (s), 120.6 (s), 120.1 (s), 119.2 (s), 116.0 (d, *J* = 23.0 Hz), 115.2 (d, *J* = 22.7 Hz), 107.4 (s), 107.1 (s). Two conformers of similar distribution

^{13}C NMR (126 MHz, $CDCl_3$) δ 162.5 (s), 162.0 (d, *J* = 249.1 Hz), 143.5 (s), 138.5 (s), 137.0 (d, *J* = 3.3 Hz), 134.5 (s), 127.8 (d, *J* = 8.7 Hz), 122.2 (s), 116.9 (d, *J* = 23.0 Hz), 116.2 (s), 108.8 (s). Major conformer reported exclusively. Some minor conformer peaks visible at the limit of detection with regards to baseline noise.

^{19}F NMR (376 MHz, $CDCl_3$, proton decoupled) δ -112.61 (major conformer), -115.39 (minor conformer).

HRMS (ESI) obtained m/z 286.0494 ($M+Na^+$). Expected 286.0486.

1D NMRS (full 2D assignments are given in the Conformer Assignment section below)

^{1}H NMR (CD_3CN)

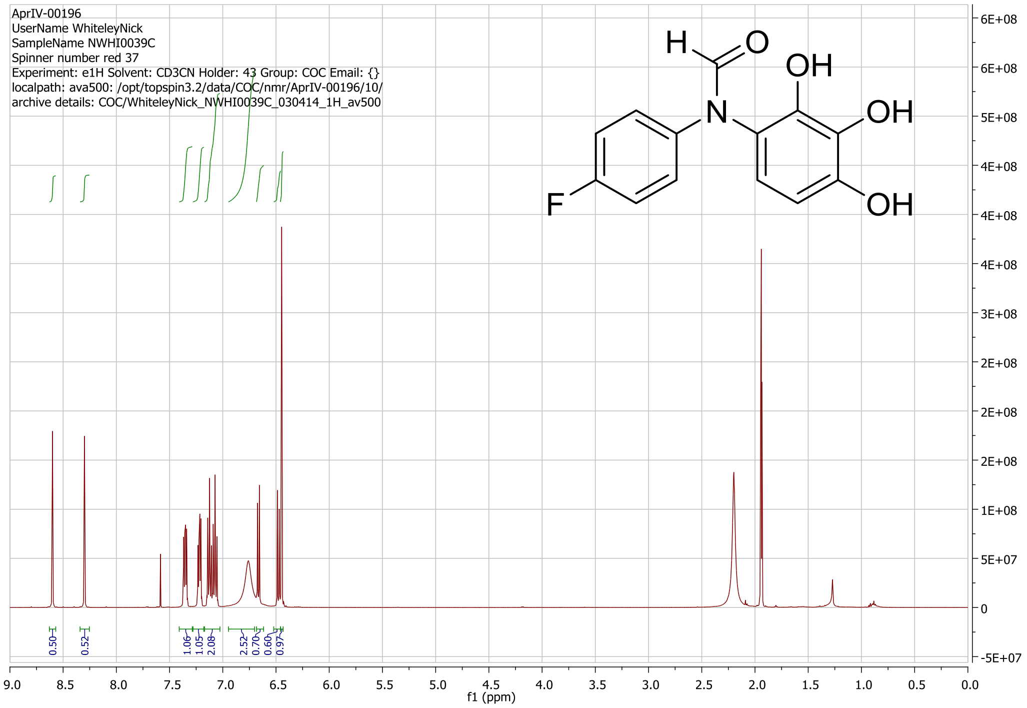

^{13}C NMR

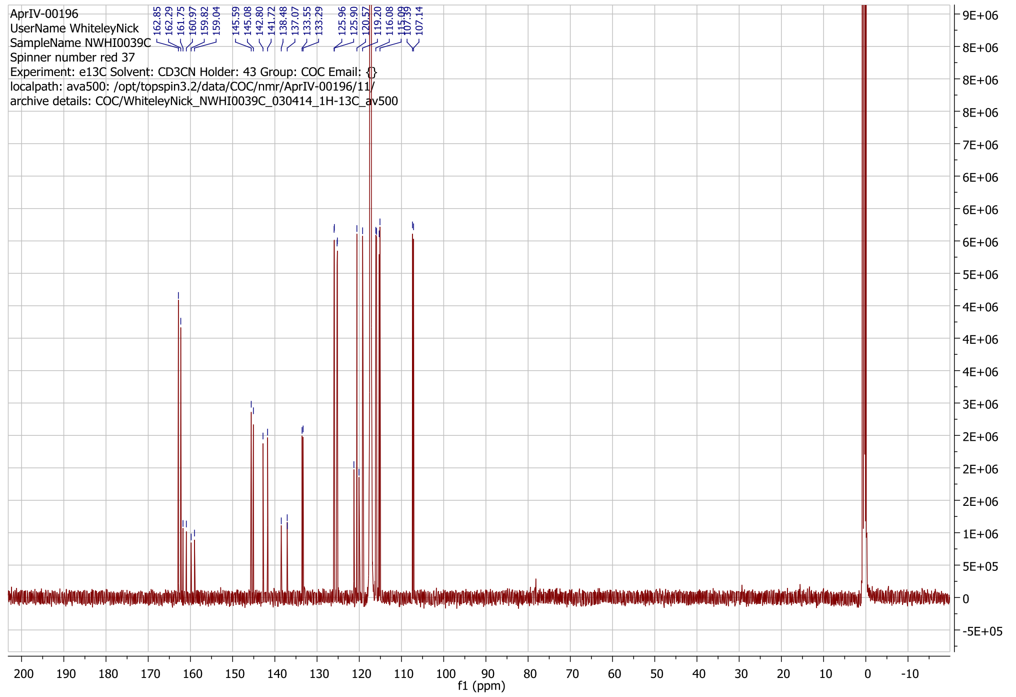

Synthesis of Compound 1-*p*-Me: *N*-(4-fluorophenyl)-*N*-(2-hydroxy-5-methyl-phenyl)formamide

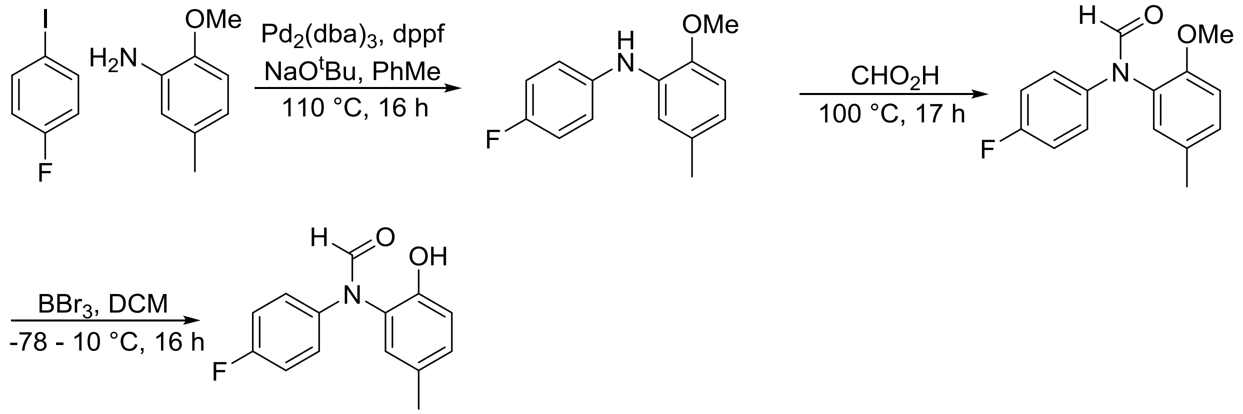

***N*-(4-fluorophenyl)-2-methoxy-5-methyl-aniline**

2-Metoxy-5-methyl-aniline (1 equiv, 10.6 mmol, 1.46 g, 1.3 mL), 4-fluoroiodobenzene (1 equiv, 10.6 mmol, 2.6 g, 1.2 mL), $Pd_2(dba)_3$ (0.01 equiv, 0.106 mmol, 97 mg), dppf (0.15 equiv, 0.16 mmol, 88 mg) and sodium *t*-butoxide (1.5 equiv, 15.9 mmol, 1.53 g) were combined in degassed toluene (15 mL) under nitrogen and the reaction heated to 110 °C for 16 h. The reaction mixture was diluted with ethyl acetate (150 mL) and washed with aqueous sodium bicarbonate (100 mL) and then brine (100 mL). Organics were dried over sodium sulphate and solvents were removed under reduced pressure. The residue was purified by flash chromatography (1-2% ethyl acetate in petroleum ether) to yield *N*-(4-fluorophenyl)-2-methoxy-5-methyl-aniline (1.88 g, 76%).

^{1}H NMR (601 MHz, $CDCl_3$) δ 7.13 – 7.08 (m, 2H), 7.02 – 6.97 (m, 2H), 6.94 (s, 1H), 6.77 (d, J = 8.1 Hz, 1H), 6.63 (d, J = 8.1 Hz, 1H), 5.98 (broad s, 1H), 3.87 (s, 3H), 2.24 (s, 3H).

^{13}C NMR (126 MHz, $CDCl_3$) δ 158.3 (d, J = 240.1 Hz), 146.1 (s), 138.8 (d, J = 2.4 Hz), 133.7 (s), 130.5 (s), 121.5 (d, J = 7.8 Hz), 119.9 (s), 116.0 (d, J = 22.4 Hz), 114.7 (s), 110.6 (s), 55.9 (s), 21.1 (s).

^{19}F NMR (376 MHz, $CDCl_3$, proton decoupled) δ -121.96 (s).

HRMS (ESI) obtained m/z 232.1134 (M+H$^+$). Expected 232.1132.

^{1}H NMR

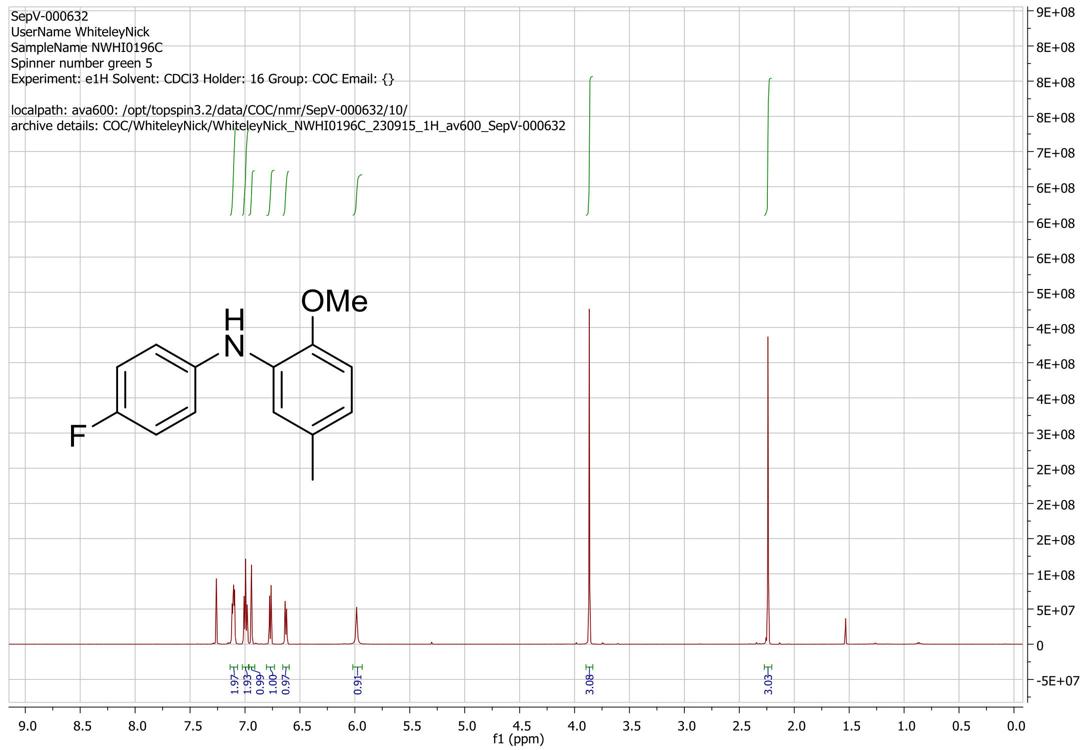

^{13}C NMR

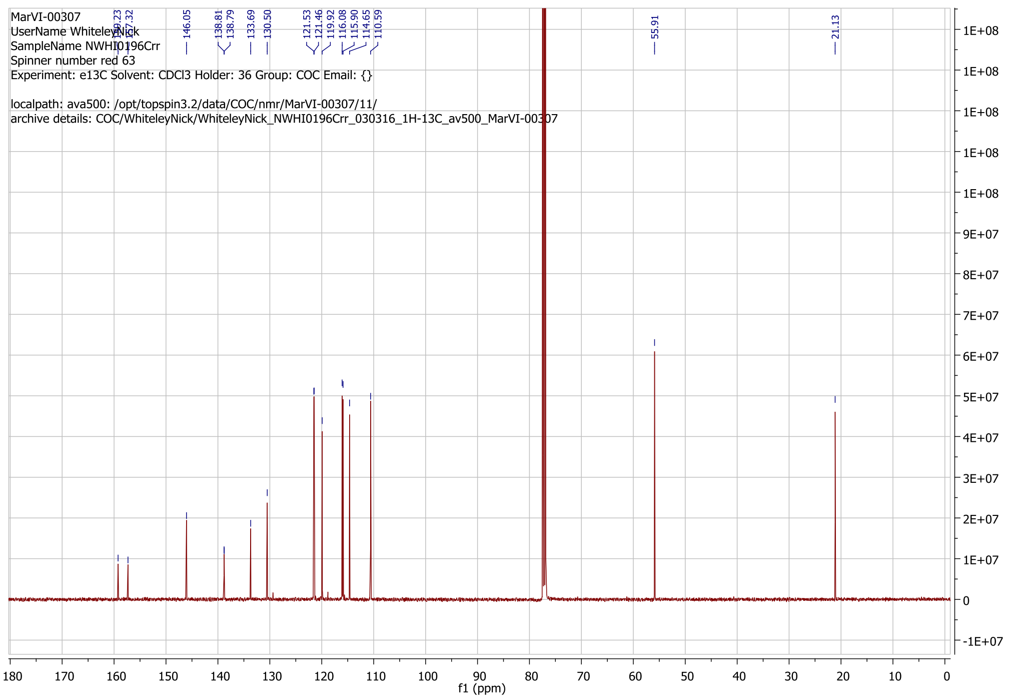

***N*-(4-fluorophenyl)-*N*-(2-methoxy-5-methyl-phenyl)formamide**

N-(4-fluorophenyl)-2-methoxy-5-methyl-aniline (1 equiv, 8.13 mmol, 1.88 g) was dissolved in formic acid (27 mL), placed under nitrogen and heated to reflux for 16 h. The reaction was then concentrated under reduced pressure. The residue was re-dissolved in ethyl acetate (100 mL), washed with saturated aqueous sodium bicarbonate (2 x 75 mL) and brine (75 mL). Organics were dried over magnesium sulfate and solvents removed under reduced pressure. The residue was purified by flash chromatography (15-20% ethyl acetate in petroleum ether) to yield *N*-(4-fluorophenyl)-*N*-(2-methoxy-5-methyl-phenyl)formamide (1.825 g, 87%)

^{1}H NMR (500 MHz, $CDCl_3$) δ 8.62 (s, 0.5H, conformer peak), 8.35 (s, 0.5H, conformer peak), 7.32 – 7.27 (m, 3H), 7.18 – 7.10 (s, 1H), 7.06 – 6.97 (m, 2H), 6.91 (d, *J* = 8.4 Hz, 1H), 3.76 (s, 3H), 2.30 (s, 1.5H, conformer peak), 2.29 (s, 1.5H, conformer peak).

^{13}C NMR (126 MHz, $CDCl_3$) δ 163.0 (s), 162.0 (s), 161.1 (d, *J* = 246.1 Hz), 160.6 (d, *J* = 245.4 Hz), 153.6 (s), 153.0 (s), 138.2 (d, *J* = 2.9 Hz), 136.4 (d, *J* = 3.1 Hz), 131.0 (d, *J* = 6.7 Hz), 130.4 (s), 130.2 (s), 130.1 (s), 129.5 (s), 127.6 (s), 126.6 (d, *J* = 8.3 Hz), 125.3 (d, *J* = 8.4 Hz), 116.3 (d, *J* = 22.8 Hz), 115.7 (d, *J* = 22.7 Hz), 112. 7 (s), 112.5 (s), 56.1 (s), 56.0 (s), 20.5 (s), 20.5 (s). Spectra contains two conformers of approximately equal distribution.

^{19}F NMR (376 MHz, $CDCl_3$, proton decoupled) δ -115.95 (s), -116.15 (s).

HRMS (ESI) obtained m/z 260.1085 ($M+H^+$). Expected 260.1081.

^{1}H NMR

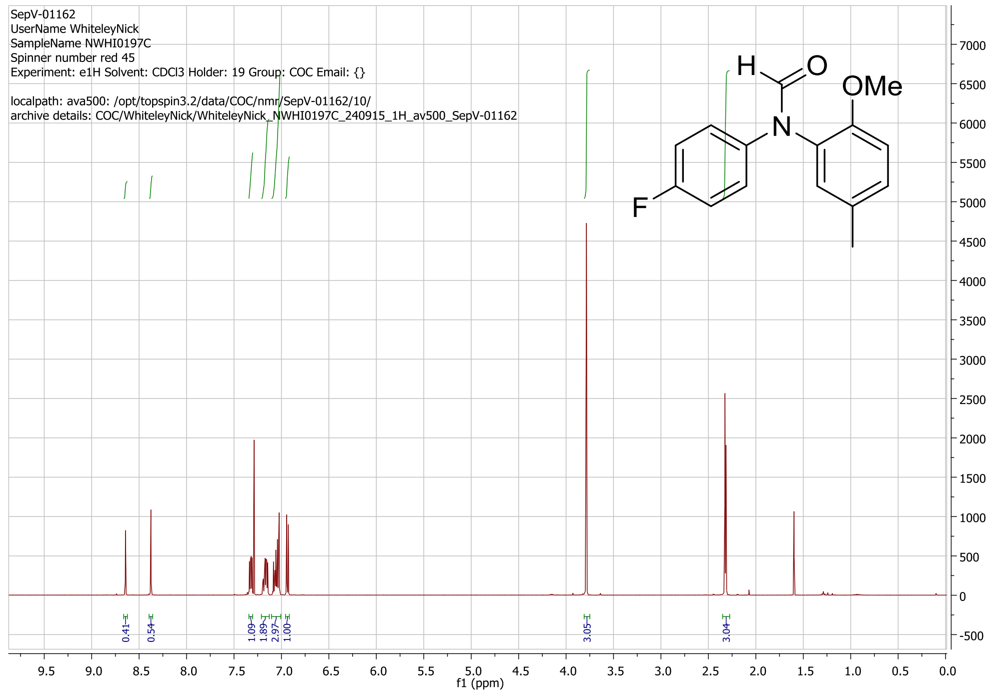

^{13}C NMR

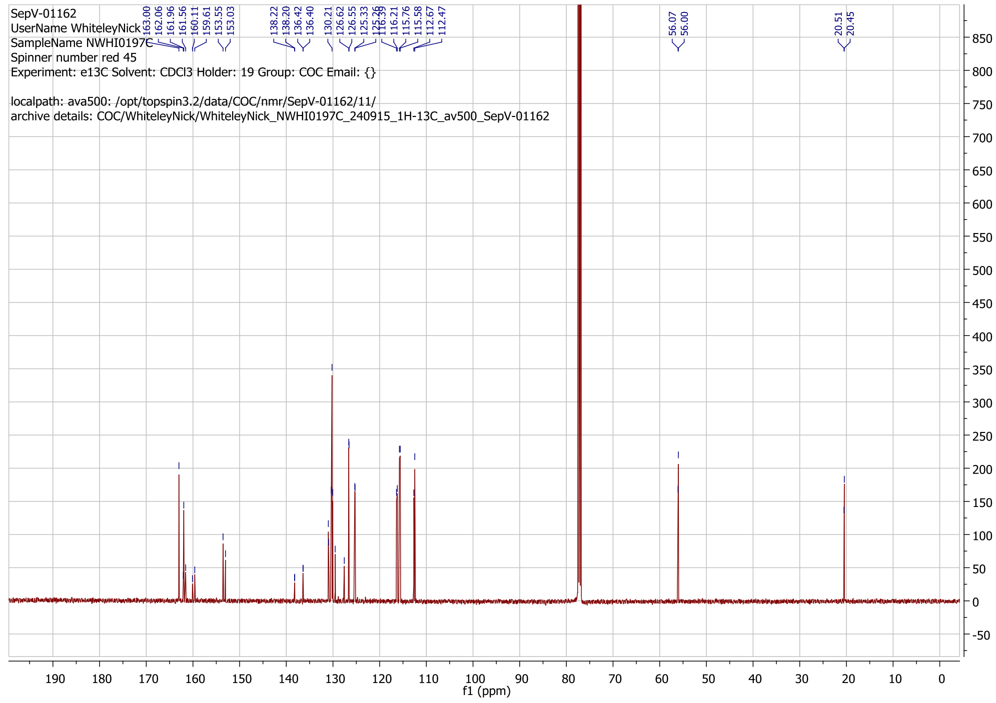

Compound 1-*p*-Me: *N*-(4-fluorophenyl)-*N*-(2-hydroxy-5-methyl-phenyl)formamide

N-(4-fluorophenyl)-*N*-(2-methoxy-5-methyl-phenyl)formamide (1 equiv, 2.71 mmol, 705 mg) was dissolved in dry DCM (14 mL) and cooled to -78 °C. A solution of BBr_3 in DCM (6 equiv, 1.57 mmol, 33% v/v, 5.2 mL) was added dropwise and the mixture stirred for 16 h allowing gradual warming to 20 °C (insulated Dewar). The reaction mixture was added dropwise to a mixture of saturated aqueous ammonium chloride (70 mL) and water (30 mL) at 0 °C. The aqueous layer was then extracted with DCM (3 x 100 mL). Combined organics were washed with brine (100 mL), dried over magnesium sulfate and solvents removed under reduced pressure. The residue was purified by flash chromatography (60% ether in hexane concentrating fractions under reduced pressure without heating) to yield *N*-(4-fluorophenyl)-*N*-(2-hydroxy-5-methyl-phenyl)formamide (281 mg, 42%).

^{1}H NMR (500 MHz, $CDCl_3$) δ 8.55 (s, 0.8H, major conformer), 8.41 (s, 0.2H, minor conformer), 7.40 – 7.34 (m, 0.4H, minor conformer), 7.24 – 7.19 (m, 1.6H, major conformer), 7.18 – 7.08 (m, 1.8H), 7.08 – 7.01 (m, 2H), 6.99 (s, 0.2H), 6.90 (d, *J* = 8.3 Hz, 0.2H), 6.61 (s, 0.8H), 6.45 (broad s, 0.8H), 5.84 (broad s, 0.2H), 2.31 (s, 0.6H), 2.21 (s, 2.4H).

^{13}C NMR (126 MHz, $CDCl_3$) δ 163.0 (s), 162.5 (s), 161.9 (d, *J* = 248.5 Hz), 160.7 (d, *J* = 246.5 Hz), 150.1 (s), 148.8 (s), 137.5 (d, *J* = 3.1 Hz), 135.7 (d, *J* = 2.6 Hz), 131.3 (s), 131.3 (s), 130.8 (s), 129.9 (s), 128.4 (s), 127.5 (d, *J* = 8.6 Hz), 126.8 (s), 126.0 (d, *J* = 8.2 Hz), 120.4 (s), 117.5 (s), 116.9 (d, *J* = 23.0 Hz), 116.0 (d, *J* = 22.7 Hz), 115.7 (s), 115.5 (s), 20.6 (s), 20.5 (s).

^{19}F NMR (471 MHz, $CDCl_3$) δ -113.14 – -113.26 (m, major conformer), -115.26 – -115.41 (m, minor conformer).

HRMS (ESI) obtained m/z 246.0929 ($M+H^+$). Expected 246.0925.

^{1}H NMR

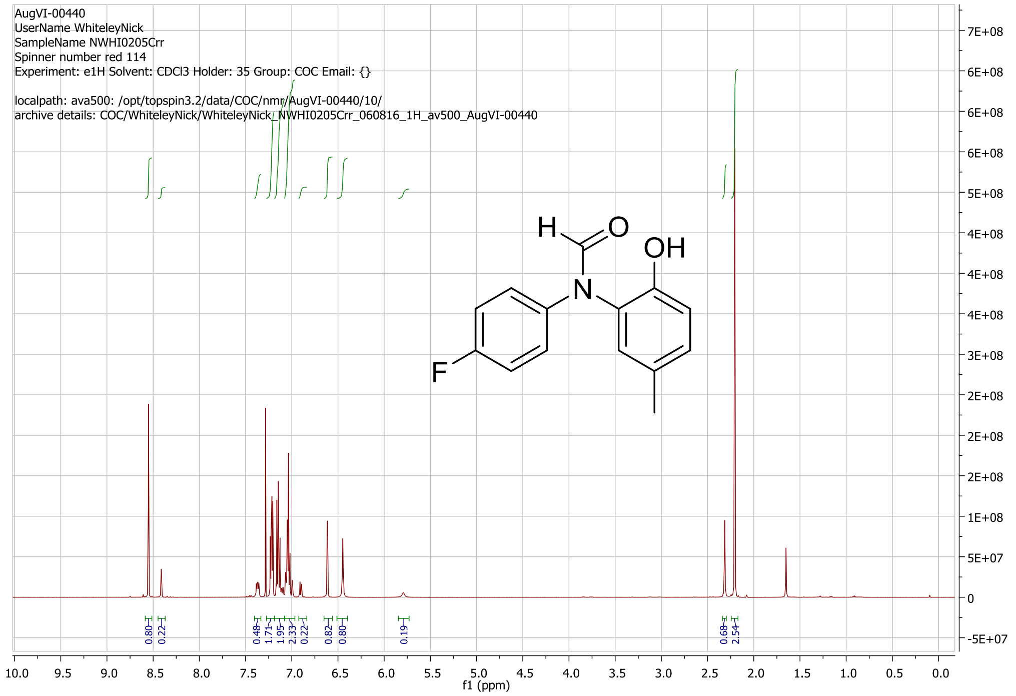

^{13}C NMR

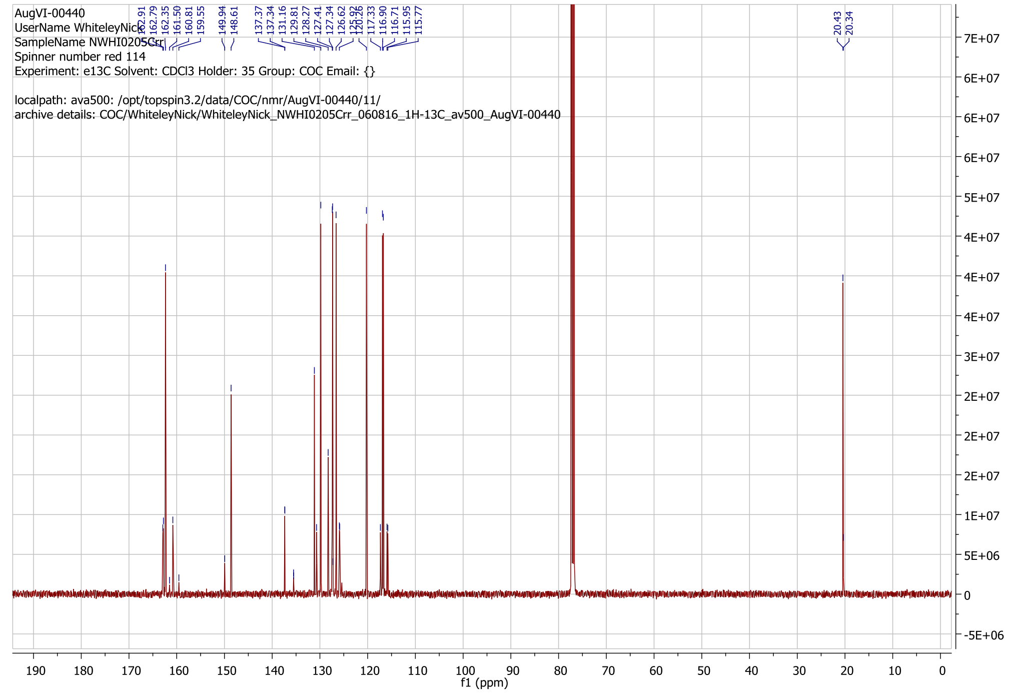

Synthesis of Compound 1-*p*-OH: *N*-(2,5-dihydroxyphenyl)-*N*-(4-fluorophenyl)formamide

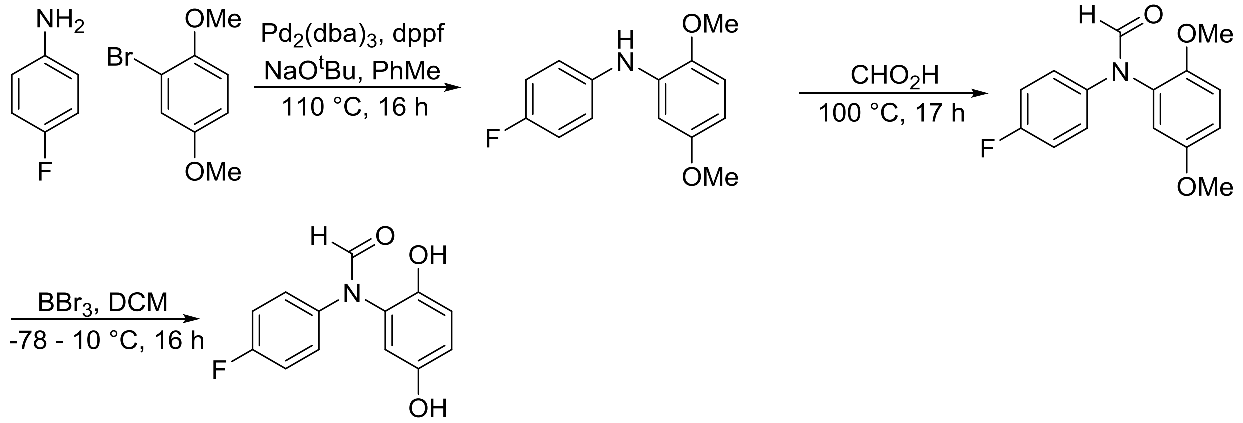

***N*-(4-fluorophenyl)-2,5-dimethoxyaniline**

1-Bromo-2,5-Dimethoxybenzene (1 equiv, 5.99 mmol, 1.3 g), 4-fluoroaniline (1 equiv, 6.35 mmol, 705 mg, 600 μL), $Pd_2(dba)_3$ (0.01 equiv, 0.06 mmol, 55 mg), dppf (0.015 equiv, 0.09 mmol, 50 mg) and sodium *t*-butoxide (1.5 equiv, 8.98 mmol, 862 mg) were combined in toluene (8.6 mL), purged with nitrogen and heated to 110 °C for 16 h. The reaction mixture was then diluted with ethyl acetate (100 mL) and washed with saturated sodium bicarbonate (3 x 75 mL) and then brine (2 x 75 mL). Organics were dried over magnesium sulfate and solvents removed under reduced pressure. The residue was purified by flash chromatography (1-2% ethyl acetate in petroleum ether gradient) to yield *N*-(4-fluorophenyl)-2,5-dimethoxy-aniline (1.08 g, 73%).

1H NMR (500 MHz, CDCl3) δ 7.17 – 7.09 (m, 1H), 7.03 – 6.96 (m, 1H), 6.78 (d, J = 8.7 Hz, 1H), 6.71 (d, J = 2.9 Hz, 1H), 6.32 (dd, J = 8.7, 2.9 Hz, 1H), 6.07 (broad s, 1H), 3.86 (s, 3H), 3.72 (s, 3H).

^{13}C NMR (126 MHz, $CDCl_3$) δ 158.5 (d, *J* = 240.7 Hz), 154.4 (s), 142.4 (s), 138.2 (d, *J* = 2.5 Hz), 135.1 (s), 122.1 (d, *J* = 7.9 Hz), 116.1 (d, *J* = 22.5 Hz), 111.2 (s), 102.5 (s), 101.0 (s), 56.3 (s), 55.8 (s).

^{19}F NMR (471 MHz, $CDCl_3$) δ -121.23 – -121.34 (m).

HRMS (ESI) obtained m/z 270.0912 ($M+H^+$). Expected 270.0901.

^{1}H NMR

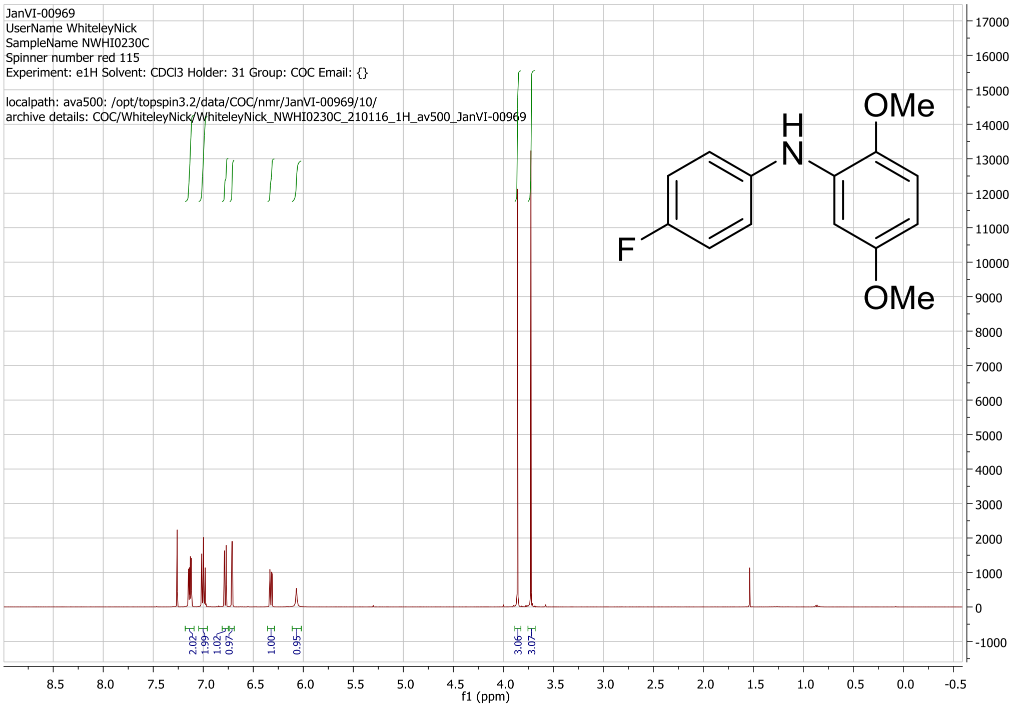

^{13}C NMR

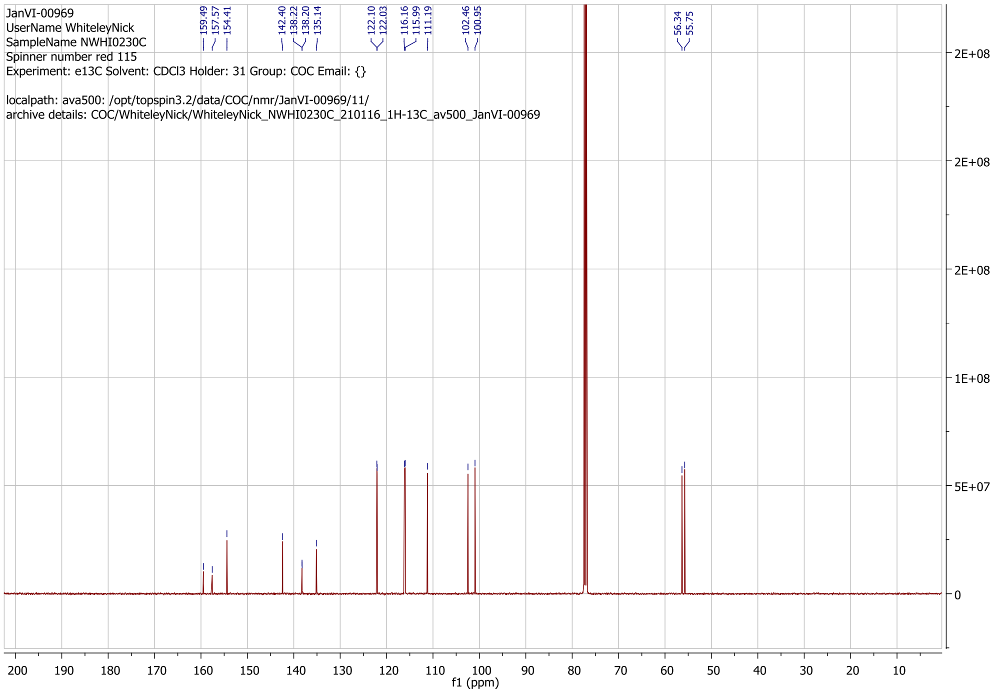

N-(2,5-dimethoxyphenyl)-N-(4-fluorophenyl)formamide

N-(4-fluorophenyl)-2,4-dimethoxy-aniline (1 equiv, 4.29 mmol, 1.06 g) was dissolved in formic acid (14 mL) and refluxed for 17 h. Solvent was removed under reduced pressure and the residue dissolved in ethyl acetate (150 mL). Organics were washed with aqueous sodium bicarbonate (3 x 100 mL), brine (2 x 100 mL) and then dried over magnesium sulfate. Solvents were removed under reduced pressure. The residue was purified by flash chromatography (10-25% ethyl acetate in petroleum ether gradient) to yield *N*-(2,5-dimethoxyphenyl)-*N*-(4-fluorophenyl)formamide (1.01 g, 86%).

1H NMR (500 MHz, CDCl3) δ 8.61 (s, 0.4H, minor conformer), 8.37 (s, 0.6H, major conformer), 7.32 – 7.27 (m, 1H), 7.16 – 7.10 (m, 2H), 7.06 – 6.98 (m, 2H), 6.96 (s, 0.4H, minor conformer), 6.94 (s, 0.6H, major conformer), 6.90 (t, J = 2.7 Hz, 0.6H, major conformer), 6.88 (t, J = 2.7 Hz, 0.4H, minor conformer), 6.76 (t, J = 3.4 Hz, 1H), 3.76 (s, 1.6H, major conformer), 3.75 (s, 1.4H, minor conformer), 3.74 (s, 1.4H, minor conformer), 3.74 (s, 1.6H, major conformer).

^{13}C NMR (126 MHz, $CDCl_3$) δ 162.9 (s, major conformer), 161.9 (s, minor conformer), 161.1 (d, *J* = 246.2 Hz, minor conformer), 160.6 (d, *J* = 245.6 Hz, major conformer), 154.1 (s, minor conformer), 154.0 (s, major conformer), 149.8 (s, major conformer), 149.4 (s, minor conformer), 138.0 (d, *J* = 2.9 Hz, minor conformer), 136.2 (d, *J* = 3.1 Hz, major conformer), 130.4 (s, major conformer), 128.5 (s, minor conformer), 126.6 (d, *J* = 8.3 Hz, major conformer), 125.3 (d, *J* = 8.4 Hz, minor conformer), 116.3 (d, *J* = 22.8 Hz, major conformer), 115.7 (d, *J* = 22.6 Hz, major conformer), 115.6 (s, major conformer), 115.4 (s, minor conformer), 114.8 (s, minor conformer), 114.4 (s, major conformer), 113.8 (s, minor conformer), 113.6 (s, major conformer), 56.6 (s, minor conformer), 56.5 (s, major conformer), 56.0 (s, major conformer), 56.0 (s, minor conformer).

^{19}F NMR (376 MHz, $CDCl_3$, proton decoupled) δ -115.75, -115.93.

HRMS (ESI) obtained m/z 276.1016 ($M+H^+$). Expected 276.1030.

^{1}H NMR

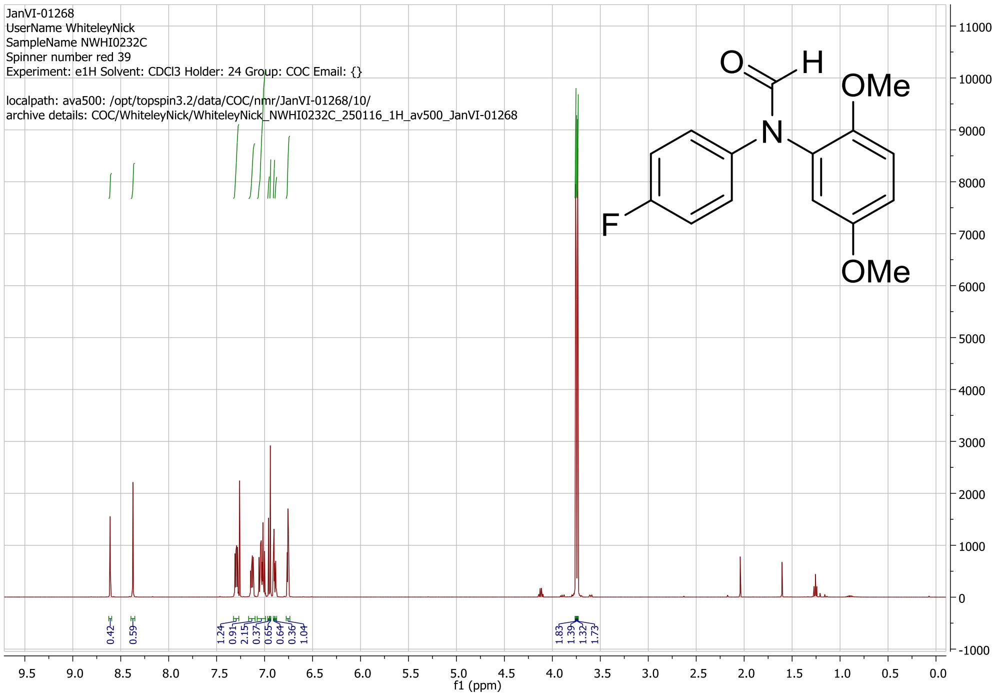

^{13}C NMR

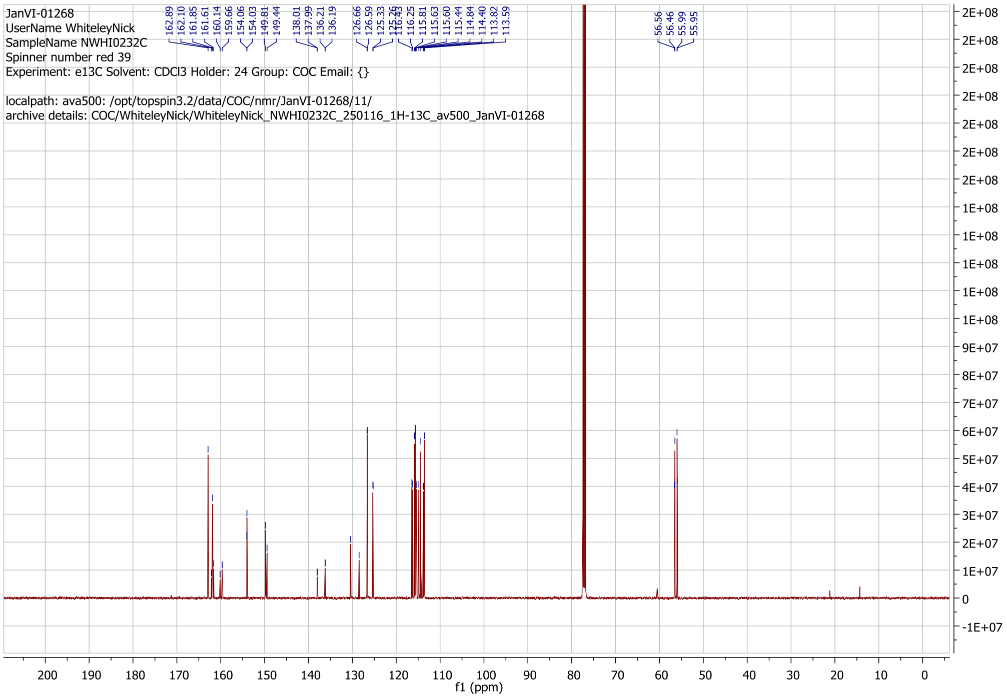

Compound 1-*p*-OH: *N*-(2,5-dihydroxyphenyl)-*N*-(4-fluorophenyl)formamide

N-(2,5-dimethoxyphenyl)-*N*-(4-fluorophenyl)formamide (1 equiv, 1.85 mmol, 510 mg) was dissolved in dry DCM (9 mL) and cooled to -78 °C. A solution of BBr_3 in DCM (11 equiv, 20.4 mmol, 33% v/v, 6.5 mL) was added dropwise and the reaction allowed to gradually warm to 20 °C over 16 h. The reaction mixture was added dropwise to a mixture of saturated ammonium chloride solution (100 mL) and water (30 mL), rinsing the reaction vessel with further DCM (100 mL). The mixture was extracted with further DCM (2 x 100 mL). Combined organics were dried over magnesium sulfate and solvents removed under reduced pressure. The residue was triturated from chloroform to yield *N*-(2,5-dihydroxyphenyl)-*N*-(4-fluorophenyl)formamide (211 mg, 46%).

^{1}H NMR (601 MHz, CD_3CN) δ 8.58 (s, 0.5H), 8.32 (s, 0.5H), 7.36 – 7.31 (m, 1H), 7.25 – 7.21 (m, 1H), 7.17 – 7.12 (m, 1H), 7.12 – 7.07 (m, 1H), 6.85 (t, J = 8.7 Hz, 1H), 6.78 – 6.71 (m, 1H), 6.68 (d, J = 2.9 Hz, 2H), 6.59 (s, 1H), 6.52 (d, J = 2.9 Hz, 2H).

^{13}C NMR (126 MHz, CD_3CN) δ 162.6 (s), 162.1 (s), 160.9 (d, J = 243.6 Hz), 160.2 (d, J = 243.1 Hz), 150.6 (s), 150.5 (s), 146.2 (s), 145.6 (s), 138.1 (d, J = 2.9 Hz), 136.6 (d, J = 2.9 Hz), 128.5 (s), 127.3 (s), 126.5 (d, J = 8.4 Hz), 125.5 (d, J = 8.6 Hz), 118.1 (s), 117.7 (s), 116.4 (s), 116.3 (s), 116.2 (s), 116.1 (d, J = 23.1 Hz), 115.3 (d, J = 22.8 Hz), 115.3 (s). Contains two conformers of approximately equal distribution (in CD_3CN)

^{19}F NMR (471 MHz, $CDCl_3$) δ -112.60 – -112.79 (m, major conformer), -114.92– -115.00 (m, minor conformer).

HRMS (ESI) obtained m/z 248.0724 ($M+H^+$). Expected 248.0645.

^{1}H NMR

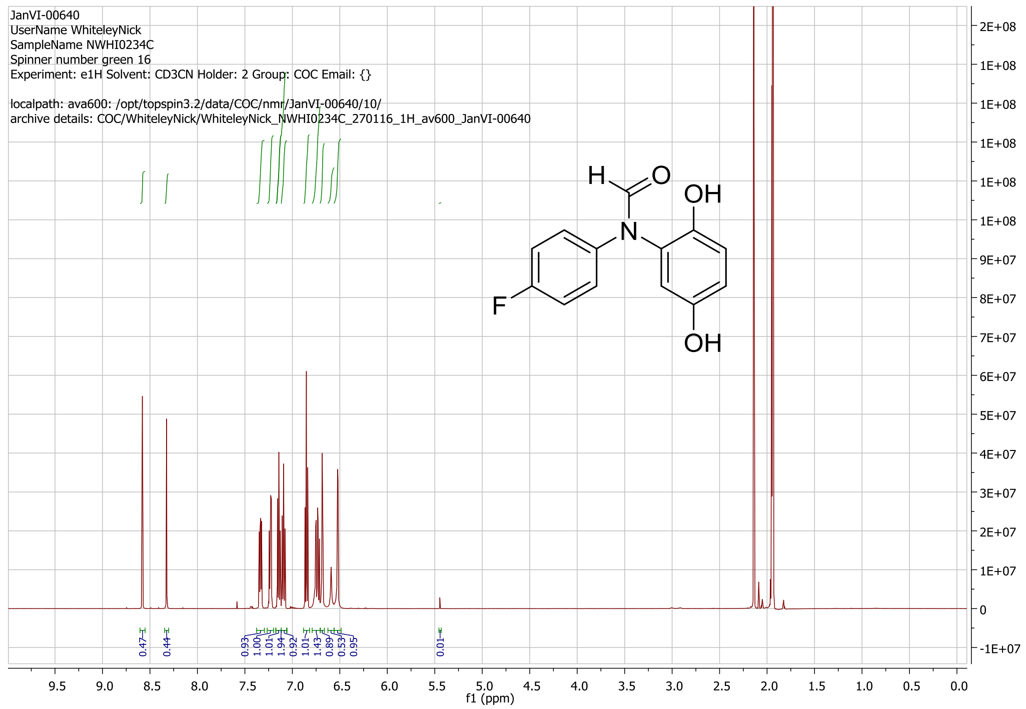

^{13}C NMR

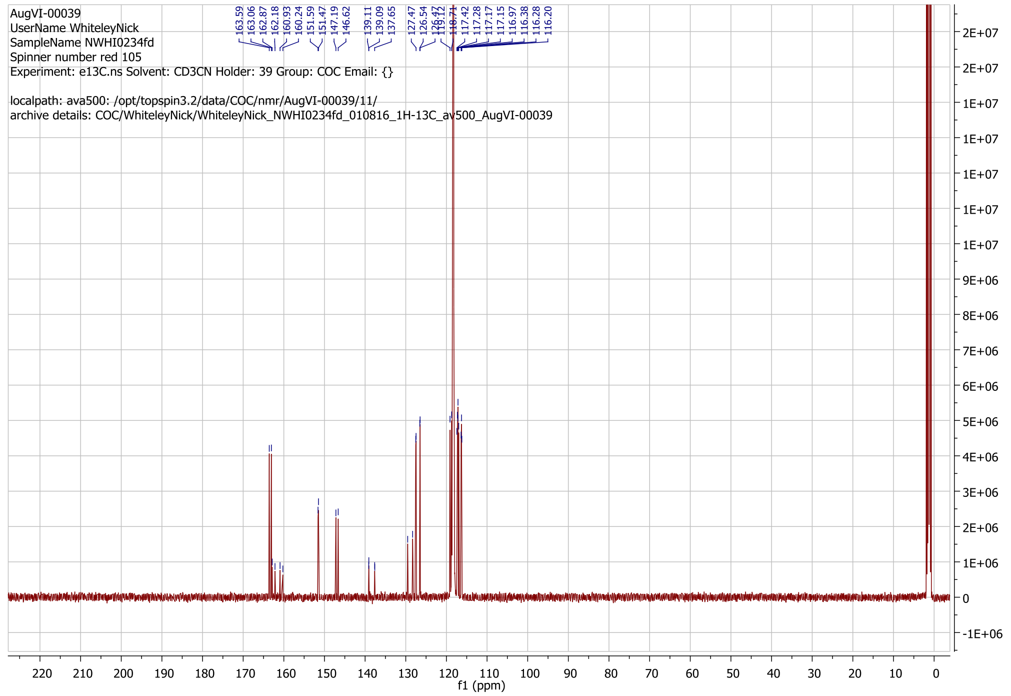

Synthesis of Compound 1-*p*-F: *N*-(4-fluoro-2-hydroxy-phenyl)-*N*-(4-fluorophenyl)formamide

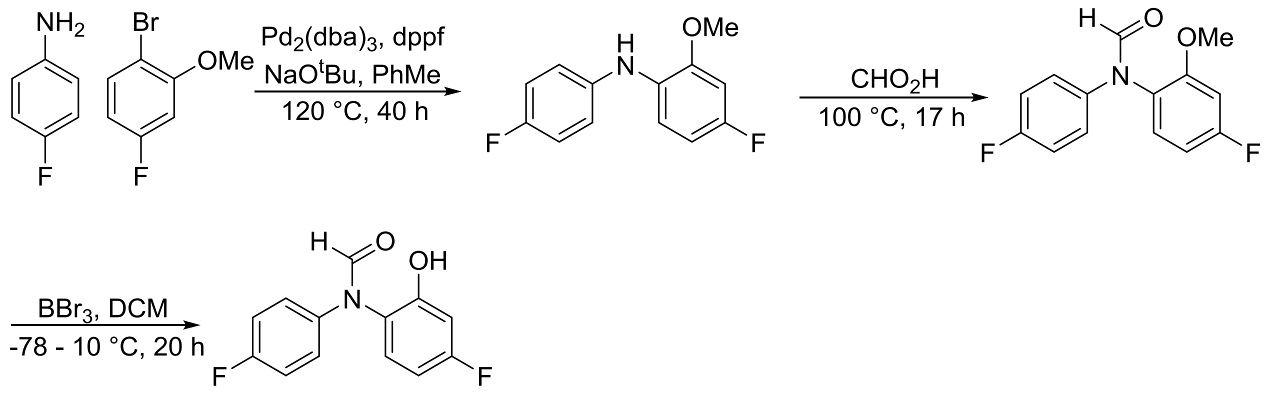

4-Fluoro-*N*-(4-fluorophenyl)-2-methoxy-aniline

2-Bromo-5-fluoroanisole (1 equiv, 6.25 mmol, 1.282 g), 4-fluoroaniline (1.35 equiv, 8.44 mmol, 937 mg, 800 µL), $Pd_2(dba)_3$ (0.01 equiv, 0.063 mmol, 57 mg), dppf (0.015 equiv, 0.093 mmol, 52 mg) and sodium *t*-butoxide (1.5 equiv, 9.38 mmol, 0.9 g) were combined in toluene (9 mL), purged with nitrogen and heated to 110 °C for 40 h. The reaction mixture was diluted with ethyl acetate (150 mL) and washed with aqueous sodium bicarbonate (2 x 100 mL) and then brine (50 mL). Organics were dried over magnesium sulfate and solvents were removed under reduced pressure. The residue was purified by flash chromatography (3% ethyl acetate in petroleum ether) to yield crude product which was re-purified flash chromatography (1-3% ethyl acetate in petroleum ether) to yield 4-fluoro-*N*-(4-fluorophenyl)-2-methoxy-aniline (961 mg, 65%) as an oil.

^{1}H NMR (500 MHz, $CDCl_3$) δ 7.08 – 6.91 (m, 5H), 6.65 (dd, J = 10.1, 2.7 Hz, 1H), 6.60 – 6.54 (m, 1H), 5.76 (broad s, 1H), 3.87 (s, 3H).

^{13}C NMR (126 MHz, $CDCl_3$) δ 158.1 (d, J = 239.8 Hz), 157.4 (d, J = 238.5 Hz), 149.5 (d, J = 9.6 Hz), 139.4 (d, J = 2.4 Hz), 129.8 (d, J = 2.8 Hz), 120.4 (d, J = 7.8 Hz), 116.0 (d, J = 22.5 Hz), 115.6 (d, J = 9.3 Hz), 106.5 (d, J = 21.9 Hz), 99.5 (d, J = 27.1 Hz), 56.0 (s).

^{19}F NMR (471 MHz, $CDCl_3$) δ -120.97 (s), -122.17 (s).

HRMS (ESI) obtained m/z 236.0889 (M+H$^+$). Expected 236.0081.

^{1}H NMR

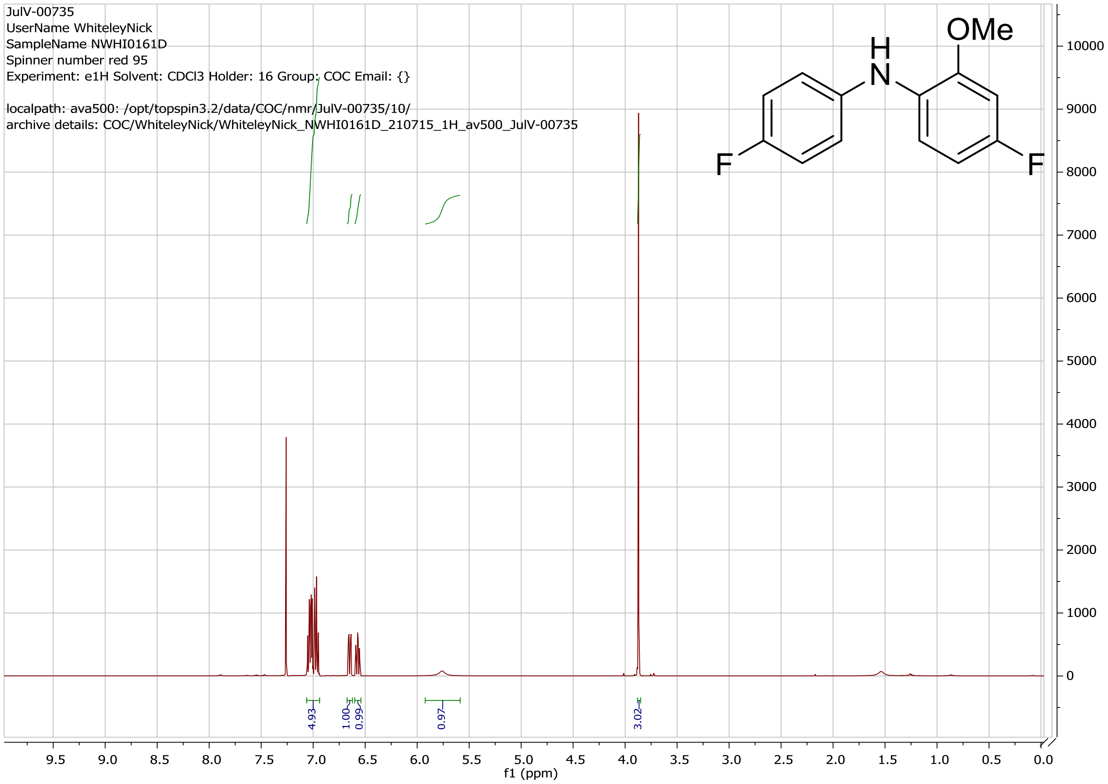

^{13}C NMR

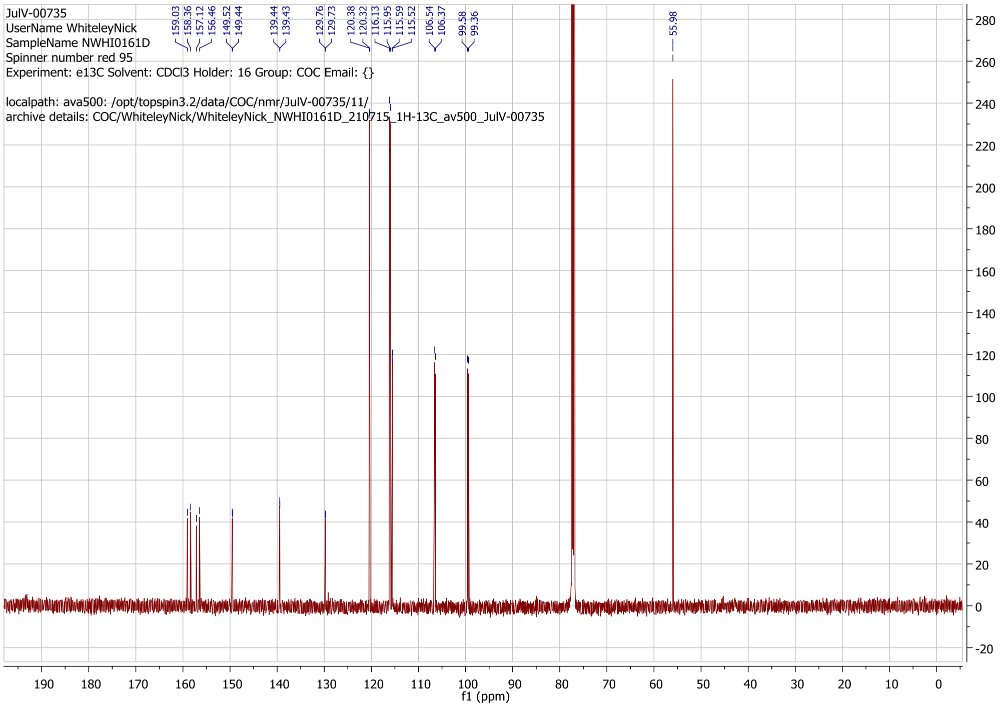

***N*-(4-fluoro-2-methoxy-phenyl)-*N*-(4-fluorophenyl)formamide**

4-Fluoro-*N*-(4-fluorophenyl)-2-methoxy-aniline (1 equiv, 3.62 mmol, 961 mg) was dissolved in formic acid (12 mL) and refluxed for 17 h. Solvent was removed under reduced pressure, the residue dissolved in ethyl acetate (150 mL), washed with aqueous sodium bicarbonate (100 mL) and then brine (50 mL). Organics were dried over magnesium sulfate and solvents removed under reduced pressure. The residue was purified by flash chromatography (7-20% ethyl acetate in petroleum ether gradient) to yield *N*-(4-fluoro-2-methoxy-phenyl)-*N*-(4-fluorophenyl)formamide (1.024 g, 95%) as an oil.

^{1}H NMR (601 MHz, $CDCl_3$) δ 8.61 (s, 0.5H, conformer peak), 8.31 (s, 0.5H, conformer peak), 7.30 – 7.26 (m, 1H), 7.21 – 7.15 (m, 1H), 7.13 – 7.09 (m, 1H), 7.07 – 6.99 (m, 2H), 6.76 – 6.69 (m, 2H), 3.79 (s, 1.5H, conformer peak), 3.78 (s, 1.5H, conformer peak).

^{13}C NMR (151 MHz, $CDCl_3$) δ 163.3 (d, *J* = 248.0 Hz), 163.2 (d, *J* = 248.9 Hz), 162. 7 (s), 161.9 (s), 161.0 (d, *J* = 246.5 Hz), 160.5 (d, *J* = 245.8 Hz), 157.0 (d, *J* = 10.6 Hz), 156.3 (d, *J* = 10.6 Hz), 137.8 (d, *J* = 2.9 Hz), 136.1 (d, *J* = 3.2 Hz), 130.7 (d, *J* = 10.5 Hz), 130.5 (d, *J* = 10.6 Hz), 126.3 (d, *J* = 8.3 Hz), 125.8 (d, *J* = 3.5 Hz), 125.1 (d, *J* = 8.4 Hz), 123.8 (d, *J* = 3.6 Hz), 116.3 (d, *J* = 22.9 Hz), 115.6 (d, *J* = 22.7 Hz), 107.7 (d, *J* = 4.1 Hz), 107.6 (d, *J* = 4.1 Hz), 100.8 (d, *J* = 26.7 Hz), 100.7 (d, *J* = 26.8 Hz), 56.1 (s), 56.1 (s). Contains two conformers of similar distribution.

^{19}F NMR (471 MHz, $CDCl_3$) δ -108.52 – -108.75 (m, 0.5F), -109.35 – -109.60 (m, 0.5F), -115.48 – -115.70 (m, 0.5F), -115.73 – -115.99 (m, 0.5F).

HRMS (ESI) obtained m/z 264.0840 ($M+H^+$). Expected 264.0831.

^{1}H NMR

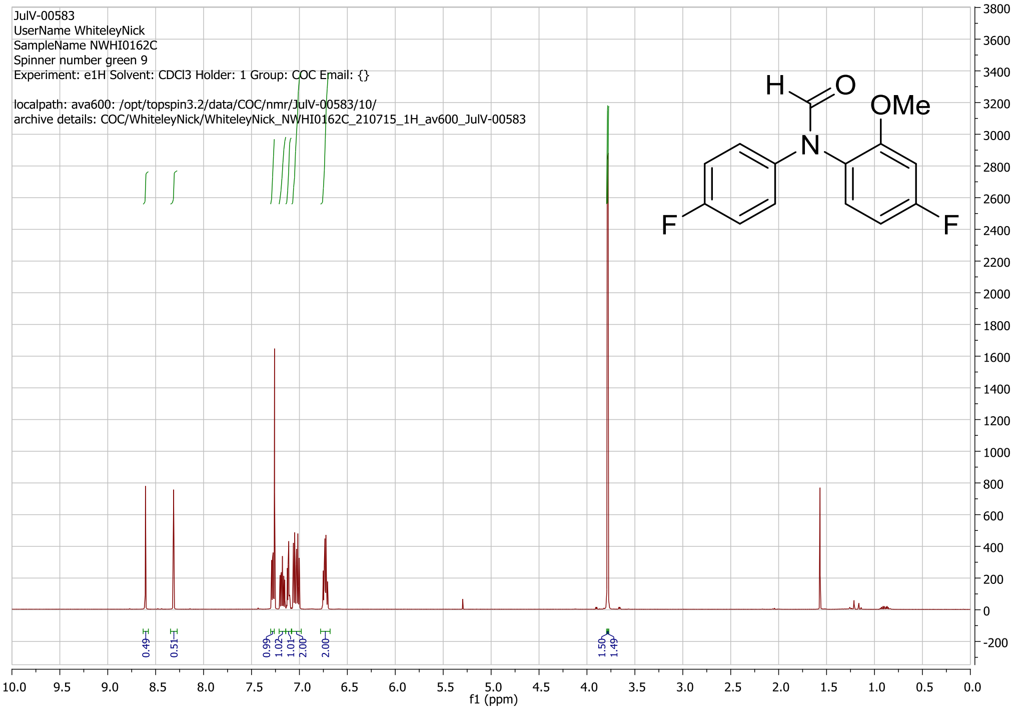

^{13}C NMR

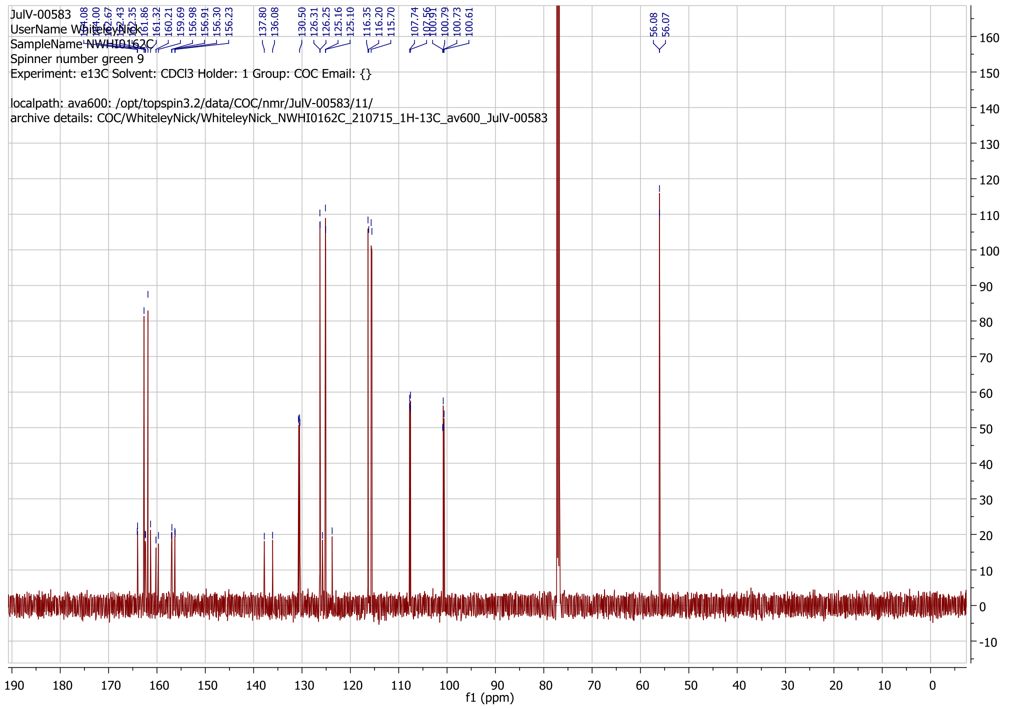

Compound 1-*p*-F: *N*-(4-fluoro-2-hydroxy-phenyl)-*N*-(4-fluorophenyl)formamide

N-(4-fluoro-2-methoxy-phenyl)-*N*-(4-fluorophenyl)formamide (1 equiv, 3.84 mmol, 1011 mg) was dissolved in dry DCM (19 mL) and cooled to -78 °C. A solution of BBr_3 in DCM (6 equiv, 23.0 mmol, 33 % v/v, 7.3 mL) was added dropwise and the mixture stirred for 16 h allowing gradual warming to ~15 °C. The reaction mixture was added dropwise to a mixture of saturated aqueous ammonium chloride (100 mL) and water (50 mL) at 0 °C. Additional DCM (100 mL) was added and organics separated. The aqueous layer was then re-extracted with DCM (2 x 100 mL). Combined organics were washed with brine (100 mL), dried over magnesium sulphate and solvents removed under reduced pressure. The residue was purified by flash chromatography (15-25% ethyl acetate in petroleum ether) to yield crude product which was re-purified by trituration/recrystalization DCM. The filtrate was repurified by trituration from 60% ether in hexane to yield *N*-(4-fluoro-2-hydroxy-phenyl)-*N*-(4-fluorophenyl)formamide (167 mg, 17%) as a white solid.

^{1}H NMR (500 MHz, $CDCl_3$) δ 8.52 (s, 0.8H, major conformer), 8.33 (s, 0.2H, minor conformer), 7.34 – 7.28 (m, 0.4H, minor conformer), 7.23 – 7.09 (m, 4.2H), 7.06 – 6.99 (m, 0.4H, minor conformer), 6.82 – 6.75 (m, 1.6H), 6.75 – 6.66 (m, 0.6H), 6.63 – 6.58 (m, 0.8H).

^{13}C NMR (126 MHz, $CDCl_3$) δ 163.2 (s, minor conformer), 162.7 (s, major conformer), 162.6 (d, *J* = 248 Hz, major conformer), 161.9 (d, *J* = 249 Hz, major conformer), 153.9 (d, *J* = 12.3 Hz, minor conformer), 152.6 (d, *J* = 12.3 Hz, major conformer), 137.2 (d, *J* = 3.2 Hz, major conformer), 135.3 (d, *J* = 2.8 Hz, minor conformer), 130.6 (d, *J* = 10.7 Hz, minor conformer), 127.7 (d, *J* = 10.6 Hz, major conformer), 127.2 (d, *J* = 8.5 Hz, major conformer), 125.9 (d, *J* = 8.5 Hz, minor conformer), 124.7 (d, *J* = 3.3 Hz, major conformer), 123.9 (d, *J* = 2.8 Hz, minor conformer) 116.9 (d, *J* = 23.0 Hz, major conformer), 116.0 (d, *J* = 22.6 Hz, minor conformer), 108.6 (d, *J* = 23.2 Hz, major conformer), 108.4 (d, *J* = 23.2 Hz, minor conformer) 107.2 (d, *J* = 24.6 Hz, major conformer), 105.1 (d, *J* = 25.8 Hz, minor conformer). Minor conformer peaks not resolved for C-F bonded carbons.

^{19}F NMR (471 MHz, $CDCl_3$) δ -109.42 (minor conformer), -112.03 (major conformer), -112.93 (major conformer), -114.86 (minor conformer).

HRMS (ESI) obtained m/z 272.0511 ($M+Na^+$). Expected 272.0494.

^{1}H NMR

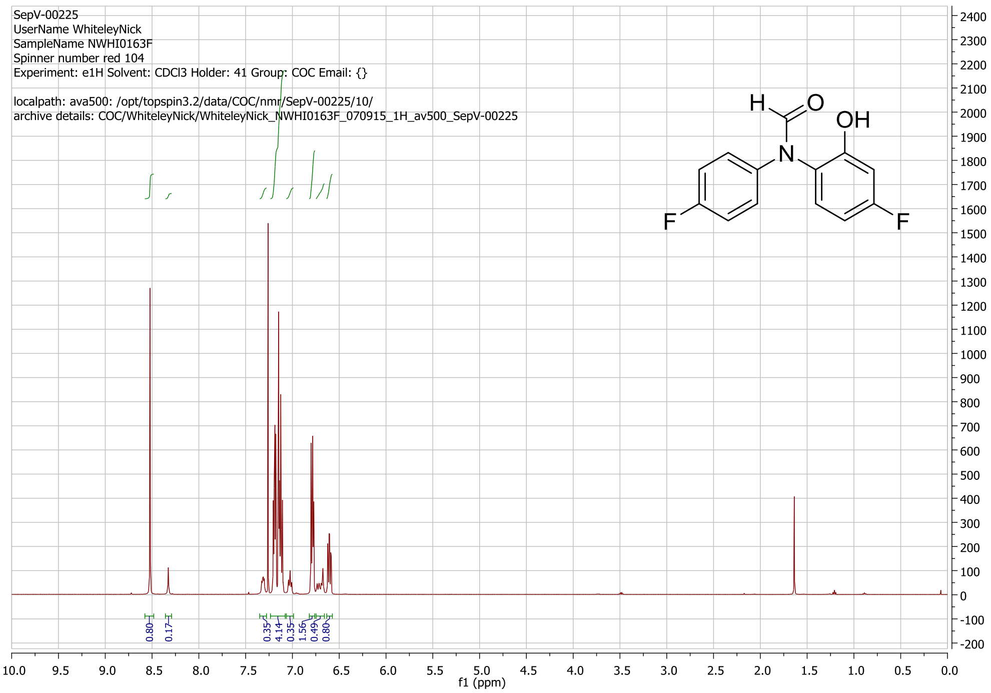

^{13}C NMR

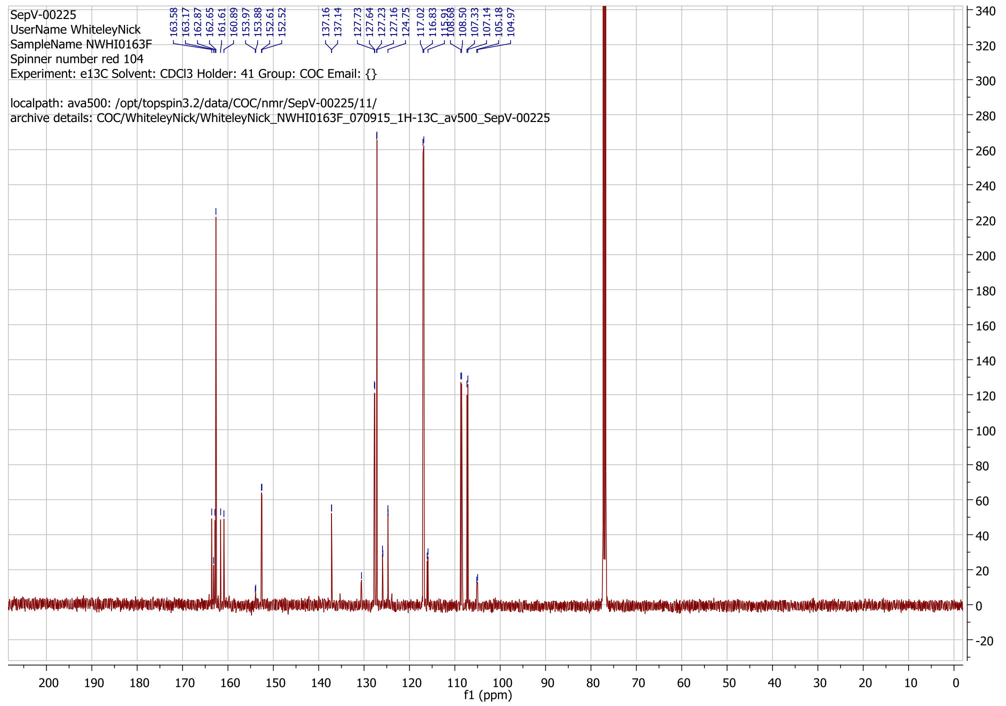

<u>Synthesis of Compound 1-*p*-CF_3: *N*-(4-fluorophenyl)-*N*-[2-hydroxy-4-(trifluoromethyl) phenyl] formamide</u>

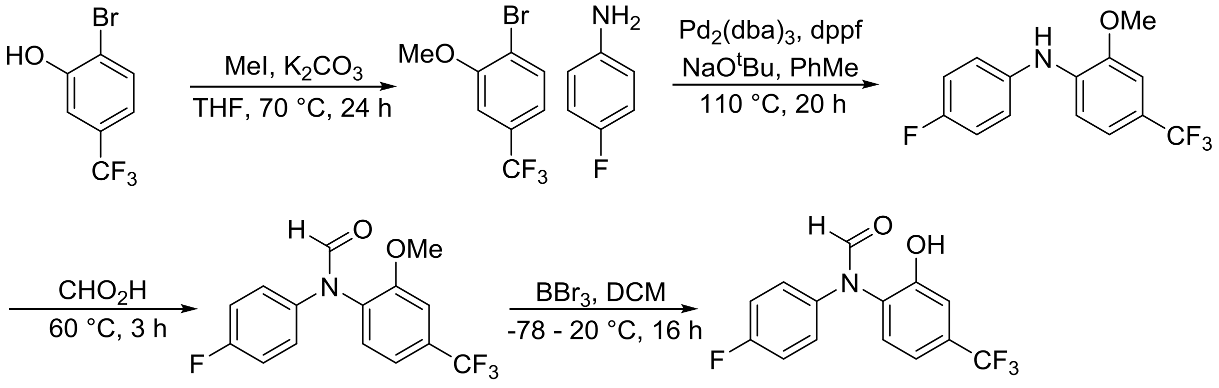

1-Bromo-2-methoxy-4-(trifluoromethyl)benzene

2-Bromo-5-trifluoromethylphenol (1 equiv, 12.7 mmol, 3.1 g) and potassium carbonate (1.3 equiv, 16.5mmol, 2.28 g) were combined in THF (25 mL). Methyl iodide (1.2 equiv, 15.3 mmol, 2.3 g, 950 μL) was added slowly and the reaction heated to 70 °C for 24 h. Methanol (20 mL) was then added and the reaction stirred for a further 1 h. The mixture was concentrated under reduced pressure, diluted with ether (150 mL) and washed with saturated sodium carbonate solution (2 x 100 mL). Organics were dried over magnesium sulfate and solvents removed under reduced pressure to yield 1-bromo-2-methoxy-4-(trifluoromethyl)benzene (2.72 g, 84%).

^{1}H NMR (400 MHz, $CDCl_3$) δ 7.65 (d, J = 7.9 Hz, 1H), 7.13 – 7.06 (m, 2H), 3.95 (s, 3H).

^{13}C NMR (126 MHz, $CDCl_3$) δ 156.4 (s), 133.9 (s), 131.1 (q, J = 32.8 Hz), 123.9 (q, J = 272.3 Hz), 118.6 (q, J = 3.9 Hz), 116.0 (d, J = 1.3 Hz), 108.7 (q, J = 3.7 Hz), 56.6 (s).

^{19}F NMR (471 MHz, $CDCl_3$) δ -62.71.

HRMS (EI) obtained m/z 253.9586 (M+H$^+$). Expected 253.9586.

^{1}H NMR

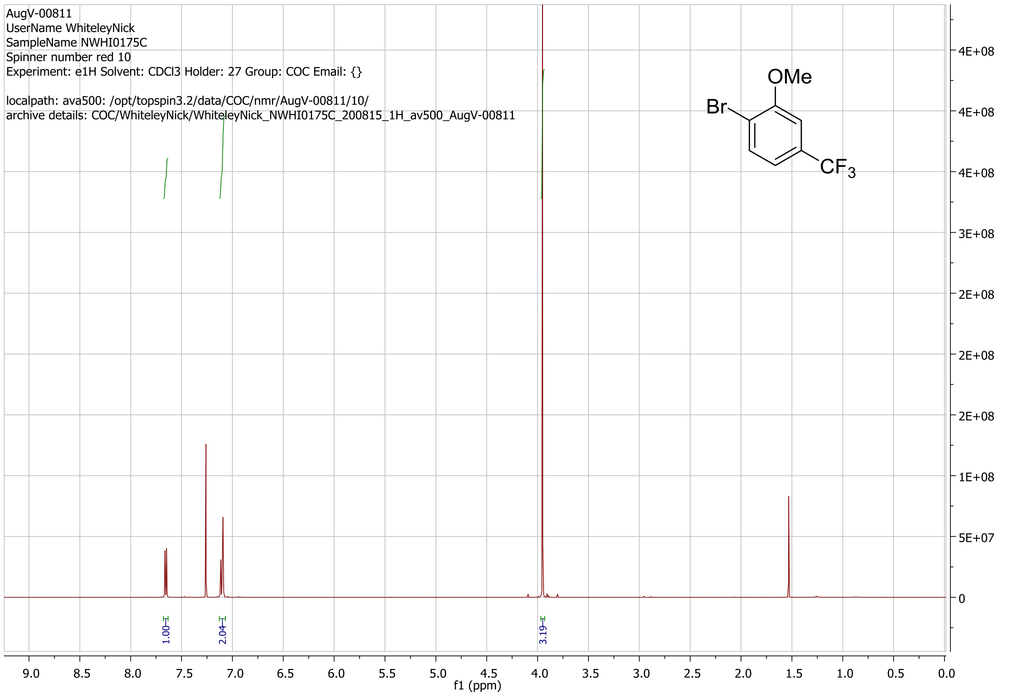

^{13}C NMR

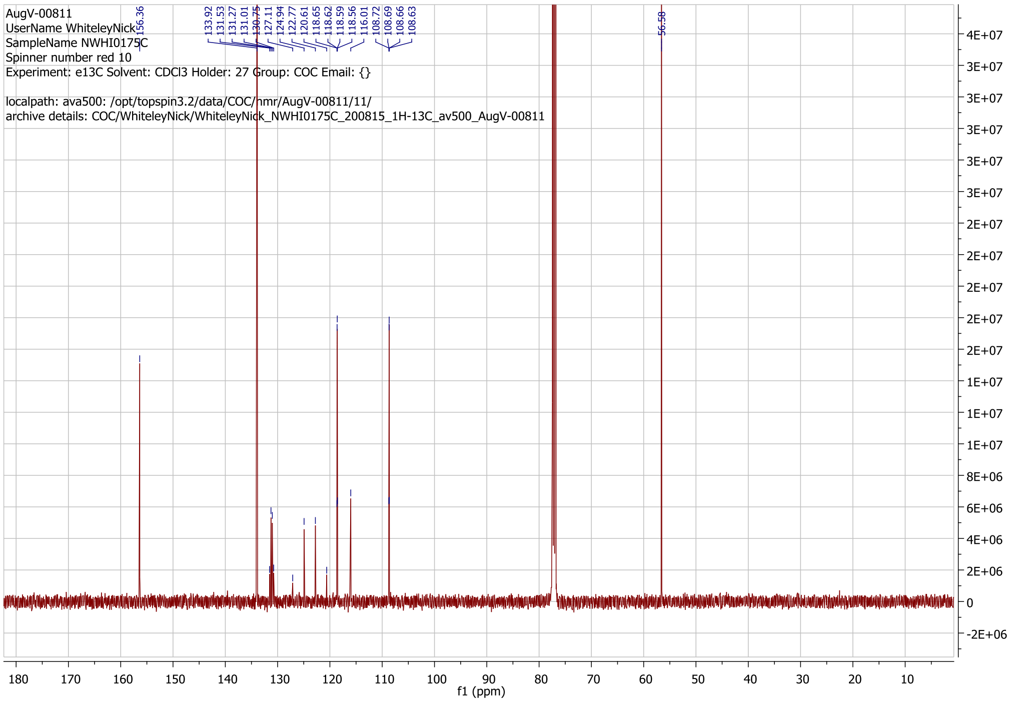

***N*-(4-fluorophenyl)-2-methoxy-4-(trifluoromethyl)aniline**

1-Bromo-2-methoxy-4-(trifluoromethyl)benzene (1 equiv, 10.7 mmol, 2.716 g), 4-fluoroaniline (1 equiv, 10.7 mmol, 1182 mg, 1 mL), $Pd_2(dba)_3$ (0.01 equiv, 0.11 mmol, 98 mg), dppf (0.02 equiv, 0.15 mmol, 89 mg) and sodium *t*-butoxide (1.5 equiv, 16.0 mmol, 1.53 g) were combined in toluene (11.3 mL), purged with nitrogen and heated to 110 °C for 20 h. The reaction mixture was diluted with ethyl acetate (300 mL) and washed with aqueous sodium bicarbonate (200 mL) and then brine (150 mL). Organics were dried over magnesium sulfate and solvents were removed under reduced pressure. The residue was purified by flash chromatography (2% ethyl acetate in petroleum ether gradient) to yield *N*-(4-fluorophenyl)-2-methoxy-4-(trifluoromethyl)aniline (1.74 g, 57%).

^{1}H NMR (500 MHz, $CDCl_3$) δ 7.18 – 7.14 (m, 2H), 7.12 – 7.08 (m, 1H), 7.07 – 7.01 (m, 4H), 6.26 (broad s, 1H), 3.95 (s, 3H).

^{13}C NMR (126 MHz, $CDCl_3$) δ 159.2 (d, *J* = 242.5 Hz), 146.9 (s), 137.6 (s), 137.0 (d, *J* = 2.7 Hz), 124.8 (q, *J* = 271.3 Hz), 123.5 (d, *J* = 8.0 Hz), 120.5 (q, *J* = 32.6 Hz), 118.7 (q, *J* = 4.2 Hz), 116.3 (d, *J* = 22.5 Hz), 111.1 (s), 107.1 (q, *J* = 3.6 Hz), 56.0 (s), 1.2 (s).

^{19}F NMR (376 MHz, $CDCl_3$, proton decoupled) δ -61.18 (s, 3F), -119.41 (s, 1F).

HRMS (ESI) obtained m/z 286.0849 ($M+H^+$). Expected 286.0850.

^{1}H NMR

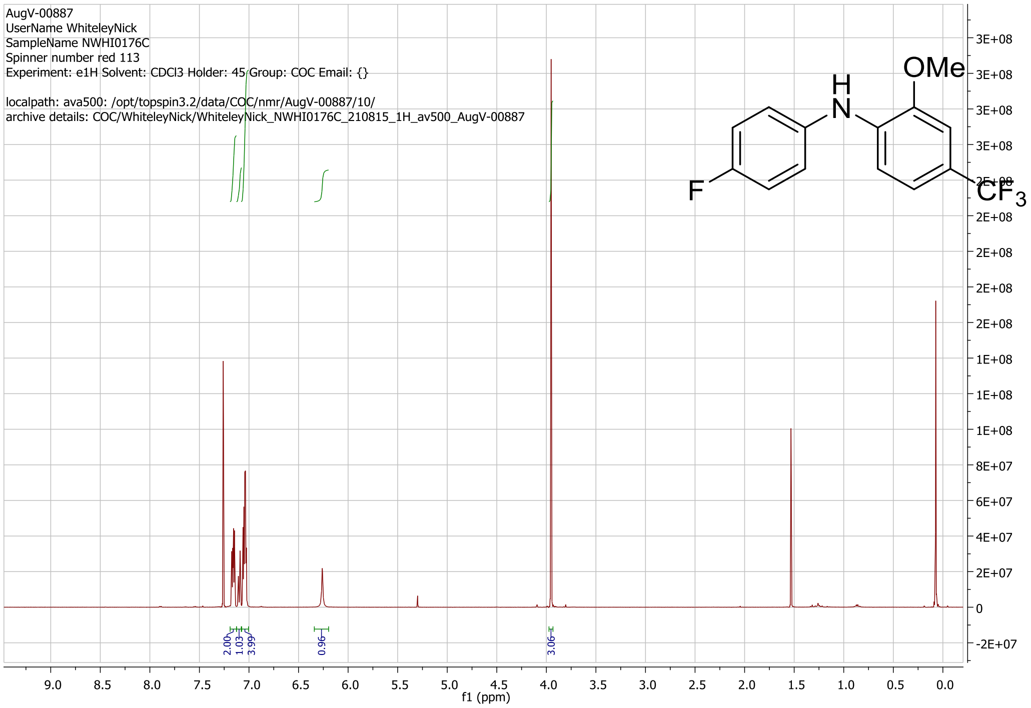

^{13}C NMR

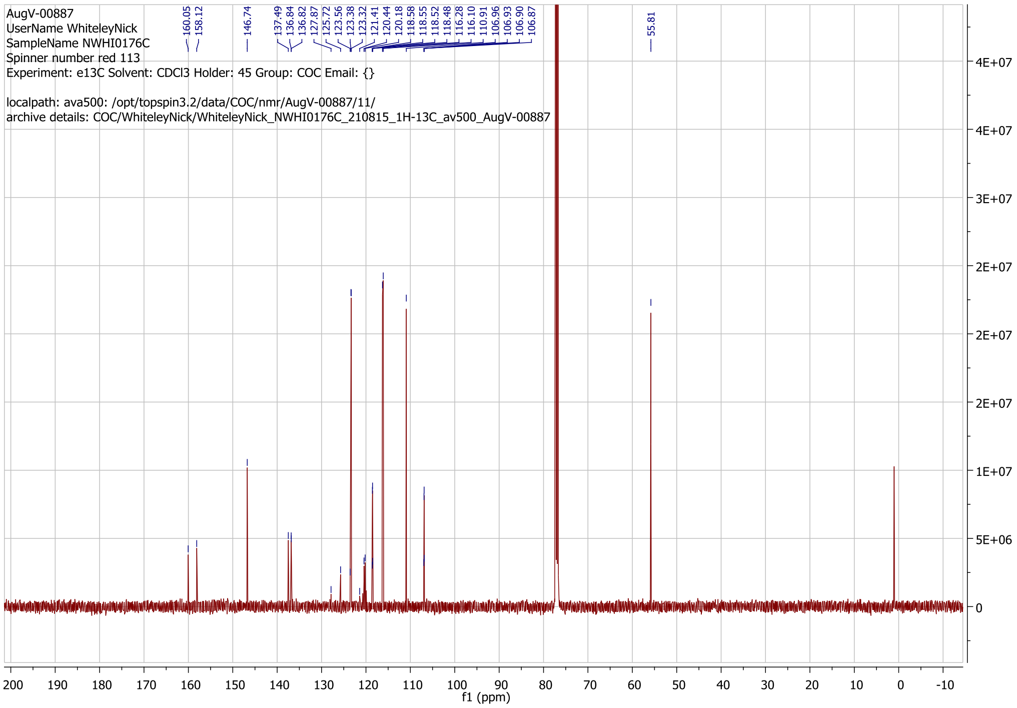

***N*-(4-fluorophenyl)-*N*-[2-methoxy-4-(trifluoromethyl)phenyl]formamide**

N-(4-fluorophenyl)-2-methoxy-4-(trifluoromethyl)aniline (1 equiv, 3.93 mmol, 1.12 g) was dissolved in formic acid (14 mL) and heated to 60 °C for 3 h. Solvent was removed under reduced pressure. The residue dissolved in ethyl acetate (150 mL), washed with aqueous sodium bicarbonate (100 mL) and then brine (50 mL). Organics were dried over magnesium sulfate and solvents removed under reduced pressure. An impurity was removed by recrystallizing from DCM, discarding a white solid (70 mg). The filtrate was concentrated and then purified by flash chromatography (10, 25% ethyl acetate in petroleum ether) to yield *N*-(4-fluorophenyl)-*N*-[2-methoxy-4-(trifluoromethyl)phenyl]formamide (300 mg, 24%).

^{1}H NMR (500 MHz, $CDCl_3$) δ 8.63 (s, 0.6H, major conformer), 8.41 (s, 0.4H, minor conformer), 7.38 – 7.29 (m, 2H), 7.28 – 7.23 (m, 1H), 7.18 – 7.12 (m, 1H), 7.12 – 7.04 (m, 2H), 3.87 (s, 1.4H), 3.86 (s, 1.6H).

^{13}C NMR (126 MHz, $CDCl_3$) δ 162.4 (s), 161.7 (s), 161.4 (d, *J* = 247.1 Hz), 161.0 (d, *J* = 246.5 Hz), 155.6 (s), 155.34 (s), 137.4 (d, *J* = 3.0 Hz), 135.7 (d, *J* = 3.0 Hz), 133.1 (s), 132.5 – 131.3 (apparent m: two overlapping quartets), 131.1 (s), 130.2 (s), 129.8 (s), 127.0 (d, *J* = 8.4 Hz), 125.6 (d, *J* = 8.5 Hz), 118.4 – 118.2 (apparent m: two overlapping quartets), 116.6 (d, *J* = 22.9 Hz), 116.0 (d, *J* = 22.7 Hz), 109.8 – 109.6 (apparent m: two overlapping quartets), 56.3 (s), 56.3 (s). Contains 2 conformers of approximately equal distribution.

^{19}F NMR (471 MHz, $CDCl_3$) δ -62.73 (s, 1.5F), -62.75 (s, 1.5F), -114.87 – -114.97 (m, 0.5F), -115.01 – -115.11 (m, 0.5F).

HRMS (ESI) obtained m/z 314.0781 ($M+H^+$). Expected 314.0799.

^{1}H NMR

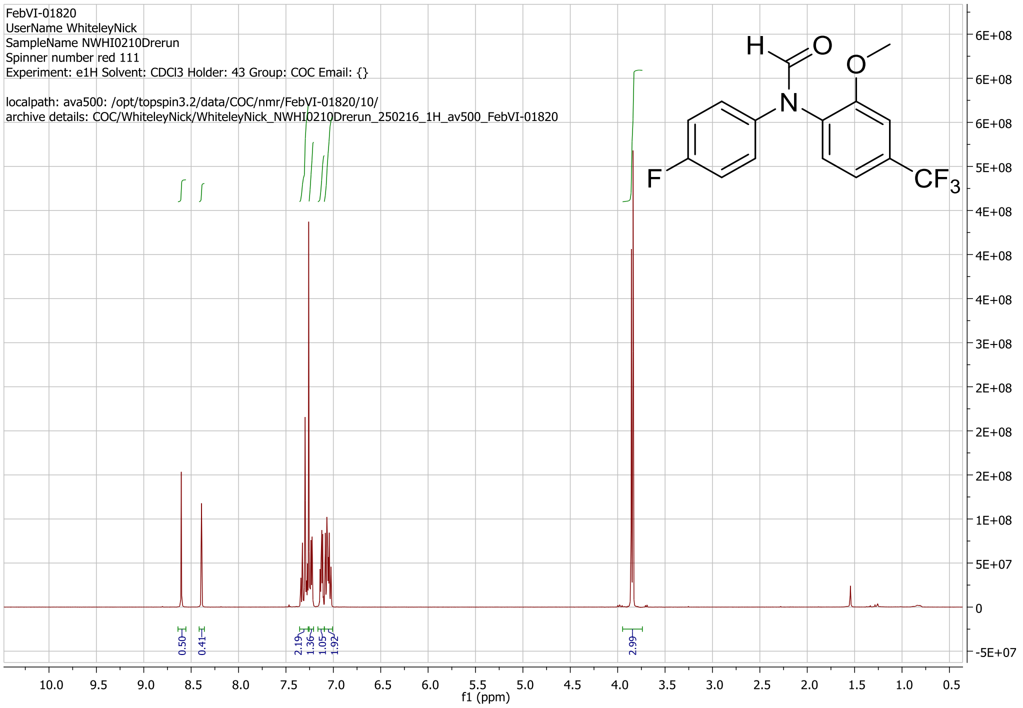

^{13}C NMR

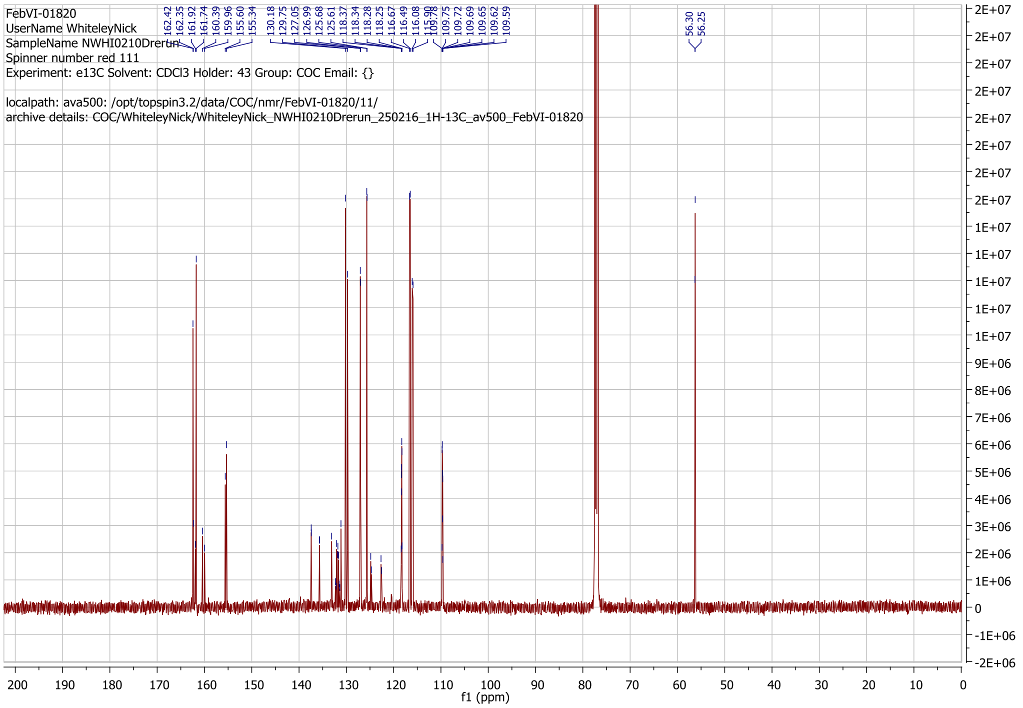

Compound 1-*p*-CF_3: *N*-(4-fluorophenyl)-*N*-[2-hydroxy-4-(trifluoromethyl)phenyl]formamide

N-(4-fluorophenyl)-*N*-[2-methoxy-4-(trifluoromethyl)phenyl]formamide (1 equiv, 0.958 mmol, 300 mg) was dissolved in dry DCM (5 mL) and cooled to -78 °C. A solution of BBr_3 in DCM (6 equiv, 5.74 mmol, 33% v/v, 1.8 mL) was added dropwise and the mixture stirred for 16 h allowing gradual warming to 20 °C (insulated dewer). The reaction mixture was added dropwise to a mixture of saturated aqueous ammonium chloride (70 mL) and water (30 mL) at 0 °C. The aqueous layer was then extracted with DCM (3 x 100 mL). Combined organics were washed with brine (100 mL), dried over magnesium sulfate and solvents removed under reduced pressure. The residue was purified by flash chromatography (60% ether in hexane, removing solvents under reduced pressure without heating) to yield *N*-(4-fluorophenyl)-*N*-[2-hydroxy-4-(trifluoromethyl)phenyl]formamide (264 mg, 92%).

^{1}H NMR (500 MHz, $CDCl_3$) δ 8.56 (s, 1H), 8.44 (s, minor conformer, trace), 7.40 (d, *J* = 1.6 Hz, 1H), 7.25 – 7.20 (m, 2H), 7.20 – 7.14 (m, 2H), 7.13 (dd, *J* = 8.4, 1.5 Hz, 1H), 7.10 – 7.01 (broad peak, 1H), 6.88 (d, *J* = 8.4 Hz, 1H).

^{13}C NMR (126 MHz, $CDCl_3$) δ 162.9 (s), 162.3 (d, *J* = 249.8 Hz), 151.4 (s), 136.8 (d, *J* = 3.2 Hz), 131.7 (s), 131.3 (q, *J* = 33.0 Hz), 127.9 (d, *J* = 8.7 Hz), 127.2 (s), 123.5 (dd, *J* = 272.9 Hz), 118.2 (q, *J* = 3.7 Hz), 118.0 (q, *J* = 3.8 Hz), 117.3 (d, *J* = 23.1 Hz). Minor conformer peaks only seen for strongest signals, not reported.

^{19}F NMR (471 MHz, $CDCl_3$) δ -62.78 (s), -111.81 – -111.95 (m, 3F), -62.92 (s, trace, minor conformer) -114.06 – -114.16 (m, 1H), -114.06 – -114.16 (m, trace, minor conformer).

HRMS (ESI) obtained m/z 300.0638 ($M+H^+$). Expected 300.0642.

^{1}H NMR

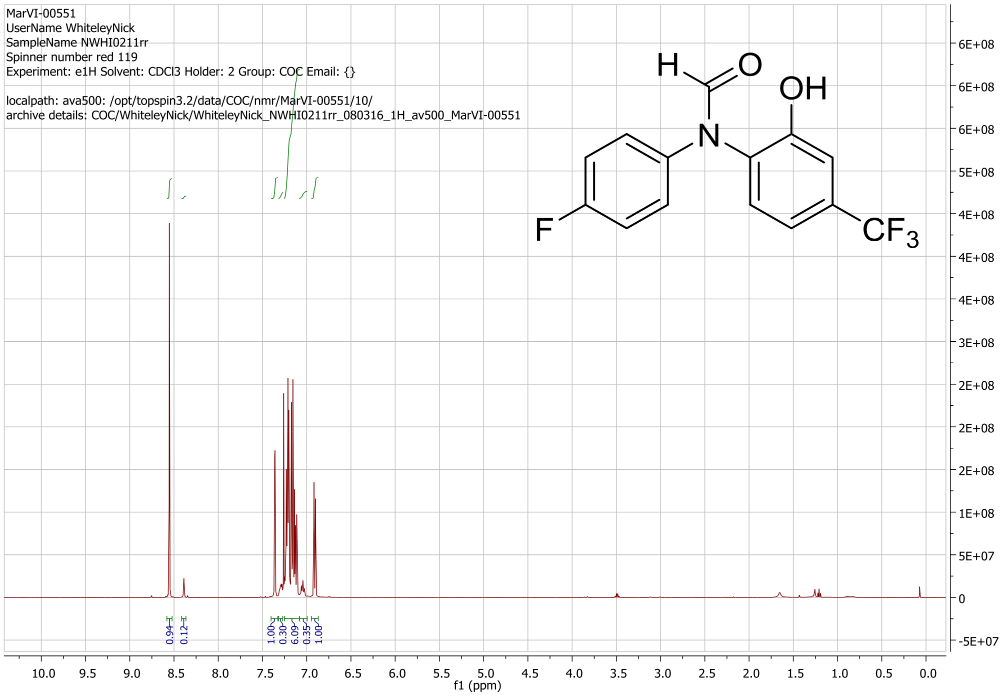

^{13}C NMR

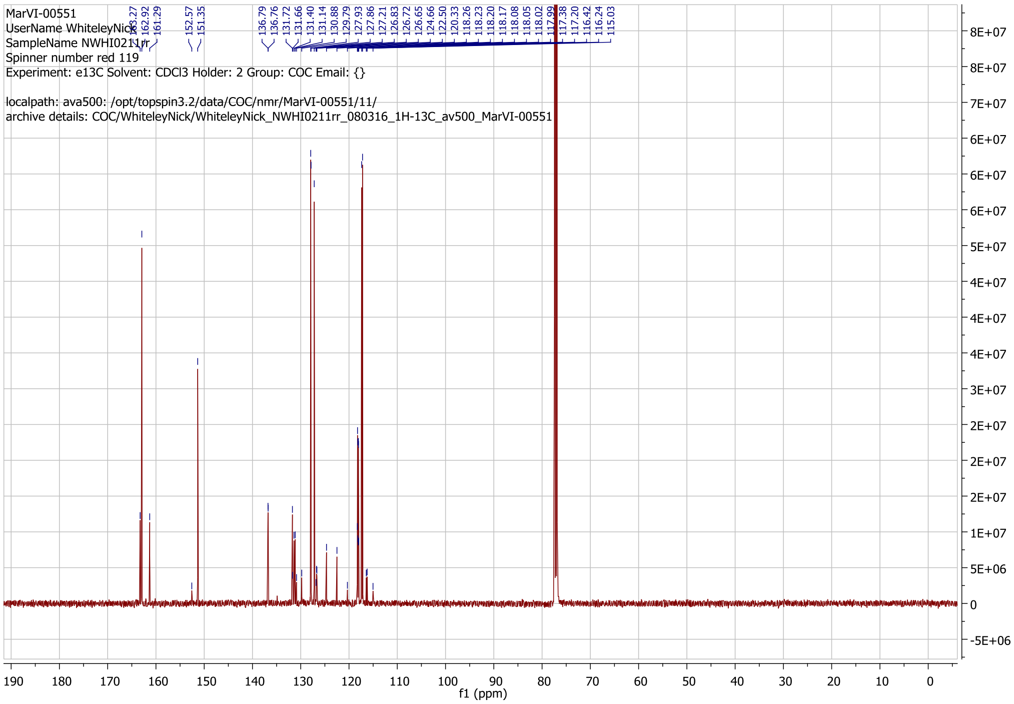

Synthesis of Compound 1-*p*-NO_2: *N*-(4-fluorophenyl)-*N*-(2-hydoxy-4-nitro-phenyl)formamide

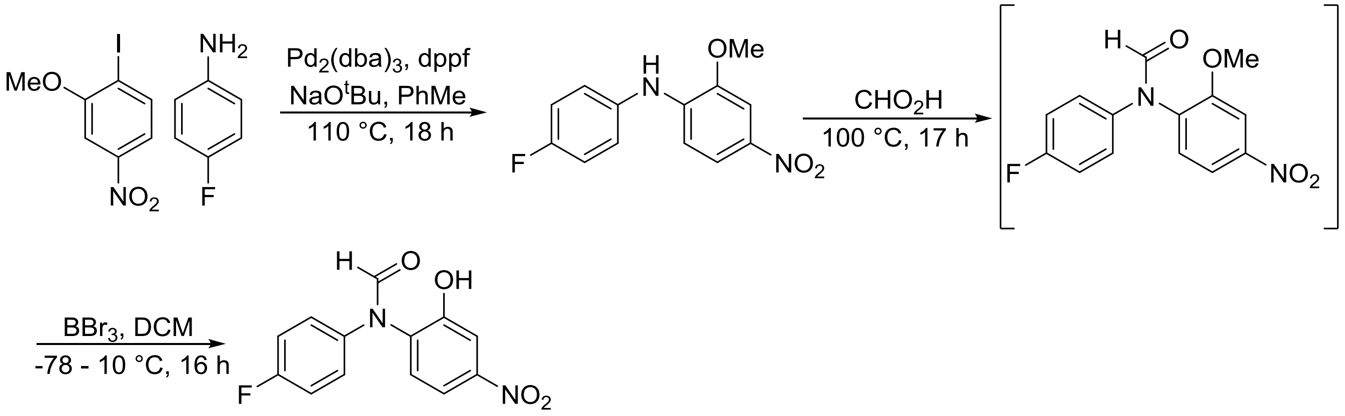

***N*-(4-fluorophenyl)-2-methoxy-4-nitro-aniline**

1-Iodo-2-methoxy-4-nitrobenzene (1 equiv, 3.91 mmol, 1.091 g), 4-fluoroaniline (1.35 equiv, 5.28 mmol, 586 mg, 500 µL), $Pd_2(dba)_3$ (0.01 equiv, 0.039 mmol, 36 mg), dppf (0.015 equiv, 0.059 mmol, 33 mg) and sodium *t*-butoxide (1.5 equiv, 5.87 mmol, 563 mg) were combined in toluene (6 mL), purged with nitrogen and heated to 110 °C for 16 h. Further toluene (15 mL) was added and heating continued for 2 h. The reaction mixture was diluted with ethyl acetate (300 mL) and washed with aqueous sodium bicarbonate (2 x 200 mL) and then brine (2 x150 mL). Organics were dried over magnesium sulfate and solvents were removed under reduced pressure. The residue was purified by flash chromatography (5-17% ethyl acetate in petroleum ether gradient) to yield *N*-(4-fluorophenyl)-2-methoxy-4-nitro-aniline (505 mg, 49%) and further product contaminated with 4-fluoroaniline (337 mg) which was triturated from ethyl acetate/hexane to yield further *N*-(4-fluorophenyl)-2-methoxy-4-nitro-aniline (170 mg). Combined yield (675 mg, 66%).

^{1}H NMR (500 MHz, $CDCl_3$) δ 7.85 – 7.80 (m, 1H), 7.73 (d, *J* = 2.4 Hz, 1H), 7.23 – 7.18 (m, 2H), 7.12 – 7.06 (m, 2H), 6.92 (d, *J* = 8.9 Hz, 1H), 6.62 (broad s, 1H), 4.01 (s, 3H).

^{13}C NMR (126 MHz, $CDCl_3$) δ 160.1 (d, *J* = 244.8 Hz), 145.9 (s), 141.4 (s), 139.1 (s), 135.4 (d, *J* = 2.9 Hz), 125.0 (d, *J* = 8.1 Hz), 119.2 (s), 116.7 (d, *J* = 22.8 Hz), 109.1 (s), 105.7 (s), 56.3 (s).

^{19}F NMR (471 MHz, $CDCl_3$) δ -117.03 – -117.12 (m).

HRMS (ESI) obtained m/z 263.0816 ($M+H^+$). Expected 263.0826.

^{1}H NMR

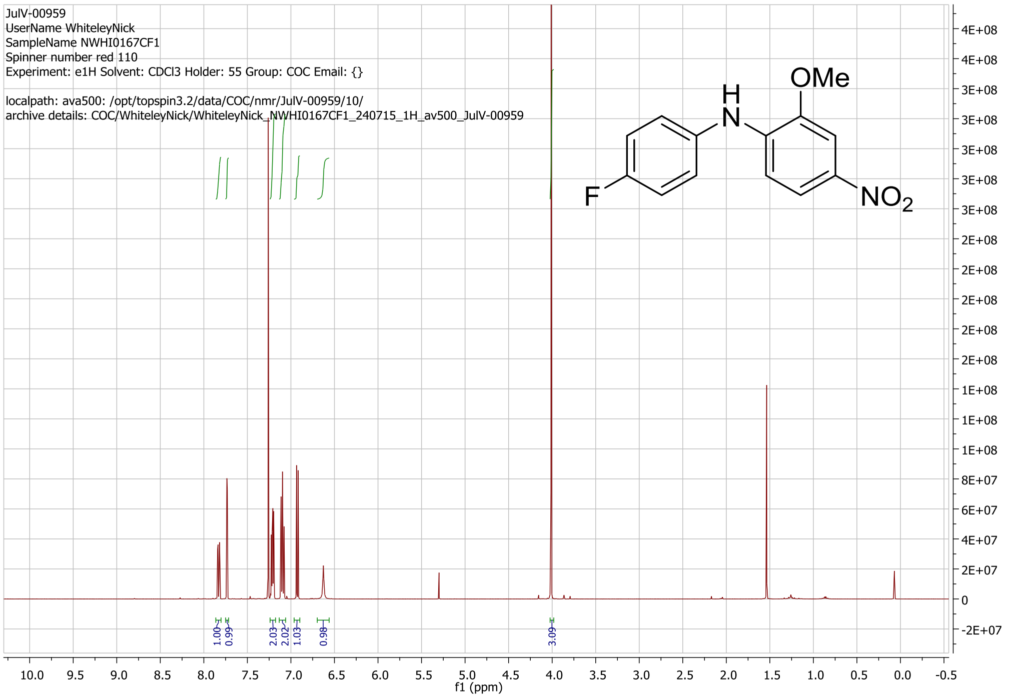

^{13}C NMR

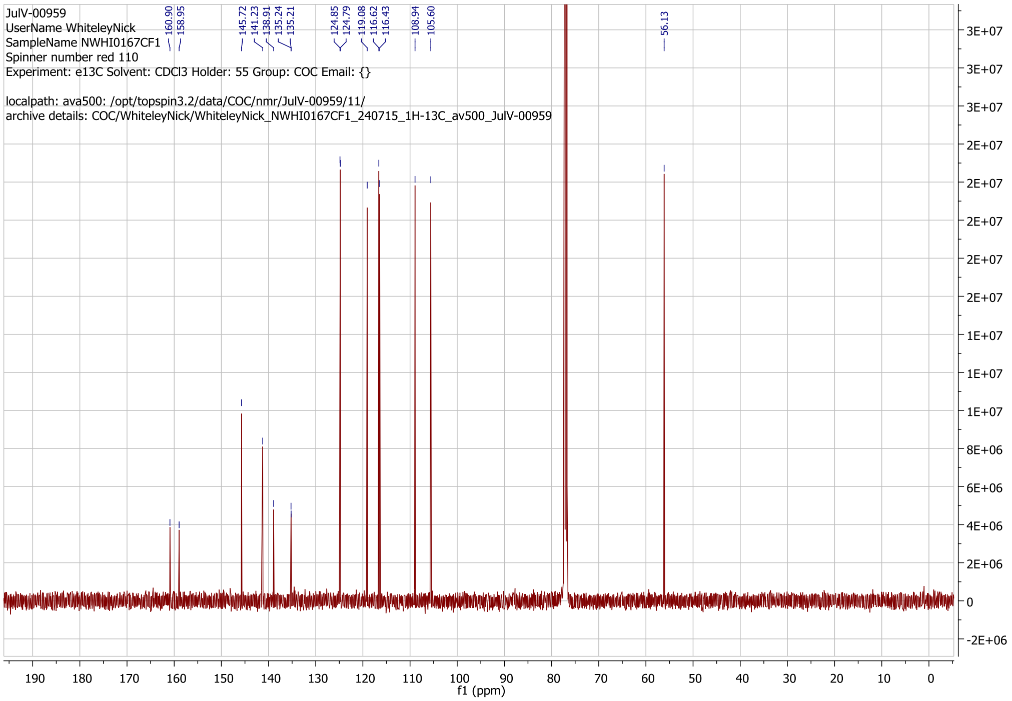

Compound 1-*p*-NO_2: *N*-(4-fluorophenyl)-*N*-(2-hydoxy-4-nitro-phenyl)formamide

N-(4-fluorophenyl)-2-methoxy-4-nitro-aniline (1 equiv, 2.55 mmol, 669 mg) was dissolved in formic acid (9 mL) and refluxed for 17 h. Solvent was removed under reduced pressure, the residue dissolved in ethyl acetate (150 mL), washed with aqueous sodium bicarbonate (100 mL) and then brine (50 mL). Organics were dried over magnesium sulfate and solvents removed under reduced pressure. The residue was purified by flash chromatography (20-45% ethyl acetate in petroleum ether gradient) to yield crude *N*-(4-fluorophenyl)-*N*-(2-methoxy-4-nitro-phenyl)formamide (525 mg, 71%). Crude *N*-(4-fluorophenyl)-*N*-(2-methoxy-4-nitro-phenyl)formamide (1 equiv, 1.79 mmol, 520 mg) was dissolved in dry DCM (9 mL) and cooled to -78 °C. A solution of BBr_3 in DCM (6 equiv, 10.7 mmol, 33 % v/v, 3.5 mL) was added dropwise and the mixture stirred for 16 h allowing gradual warming to 10 °C. The mixture was then warmed to room temperature and stirred for a further 24 h. The reaction mixture was added dropwise to a mixture of saturated aqueous ammonium chloride (70 mL) and water (30 mL) at 0 °C. The aqueous layer was then extracted with DCM (3 x 100 mL). Combined organics were washed with brine (100 mL), dried over magnesium sulphate and solvents removed under reduced pressure. The residue was purified by flash chromatography (1-1.5% methanol in chloroform) to yield crude product which was repurified by trituration from 75% ether in hexane to yield to *N*-(4-fluorophenyl)-*N*-(2-hydoxy-4-nitro-phenyl)formamide (125 mg, 25%).

^{1}H NMR (601 MHz, $CDCl_3$) δ 8.57 (s, 1H), 8.49 (s, trace, minor conformer), 7.99 (d, *J* = 2.5 Hz, 1H), 7.73 (dd, *J* = 8.9, 2.5 Hz, 1H), 7.31 (s, 1H), 7.25 – 7.17 (m, 4H), 6.91 (d, *J* = 8.9 Hz, 1H). Trace minor conformer peaks seen for most signals.

^{13}C NMR (151 MHz, $CDCl_3$) δ 163.1 (s), 162.5 (d, *J* = 250.8 Hz), 151.5 (s), 147.6 (s), 136.4 (d, *J* = 3.2 Hz), 134.8 (s), 128.3 (d, *J* = 8.8 Hz), 126.9 (s), 117.6 (d, *J* = 23.1 Hz), 116.6 (s), 116.5 (s). Major conformer observed exclusively.

^{19}F NMR (471 MHz, $CDCl_3$) δ -110.93 – -111.03 (m, 1F, major conformer), -113.31 – -113.42 (m, minor conformer, trace).

HRMS (ESI) obtained m/z 277.0613 ($M+H^+$). Expected 277.0619.

^{1}H NMR

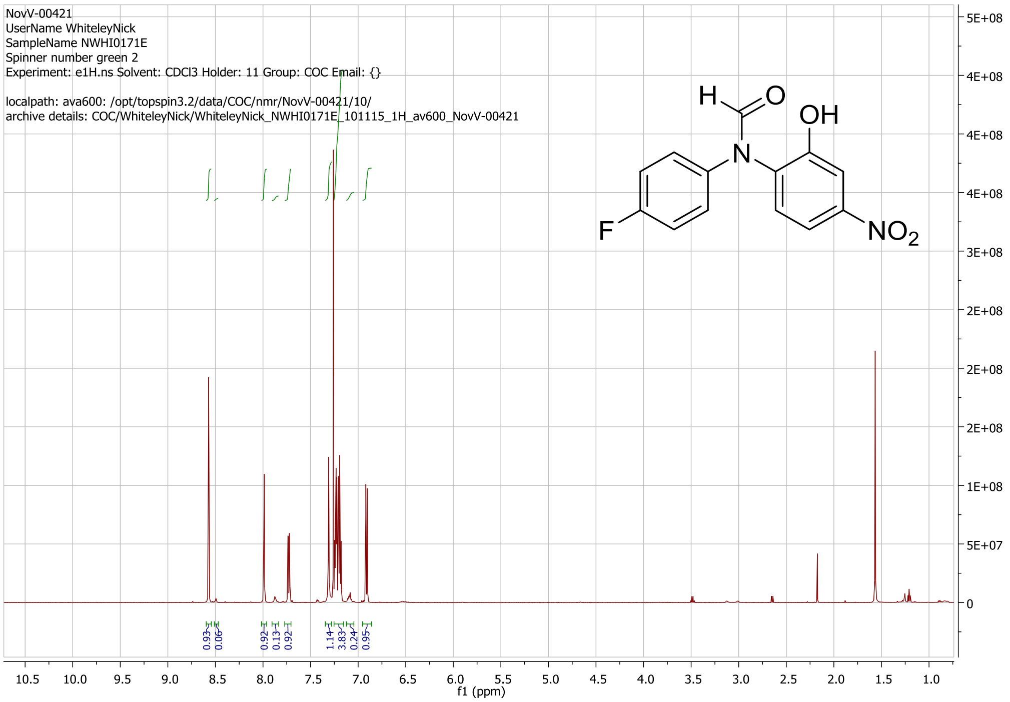

^{13}C NMR

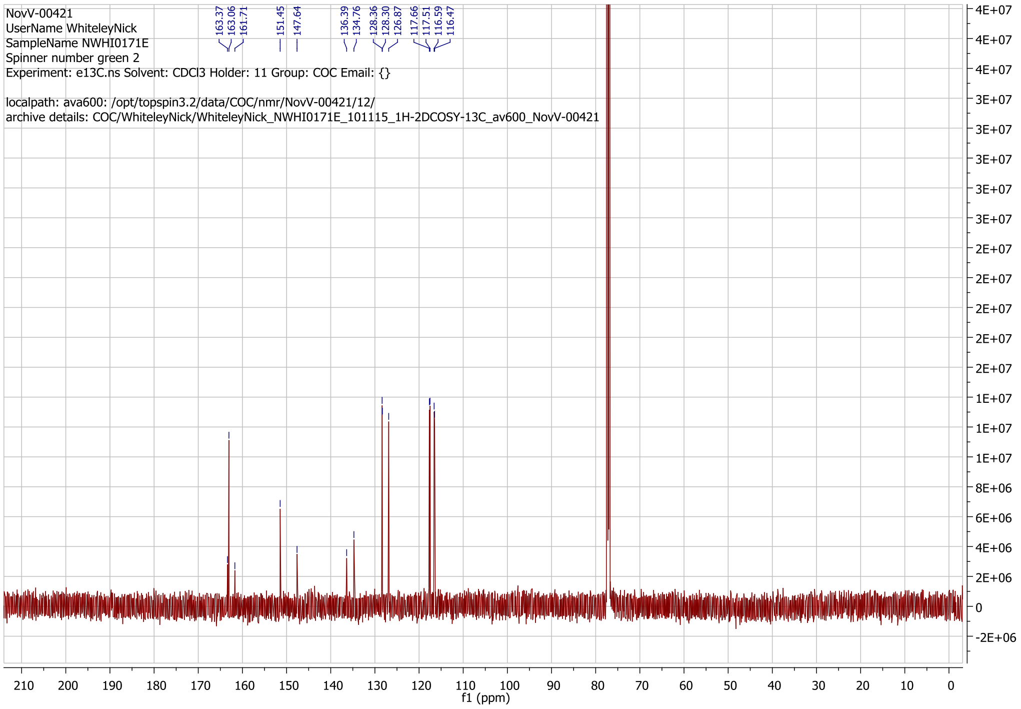

7 Molecular Balance Conformer Assignment

The assignment of molecular balance conformers was conducted using 2D NMR techniques. A key method of conformer assignment relies on *trans*-HMBC coupling between the formyl proton and an aromatic carbon of one ring.[2] Assignment of the aromatic carbons to a ring is facile *via* splitting patterns attributed to the *para*-fluoro group on the fluorinated ring. This allows accurate conformer assignment via HMBC NMR. In addition, other 2D methods (HSQC, COSY, NOESY) then being used to confirm the structure and assign signals as much as possible. It should be noted that neither NOESY or ROESY NMR show strong NOEs to the formyl proton, preventing this method from being used to accurately assign conformers.

2-D spectra are shown with relevant correlations attributed to the fluorinated ring (green) and phenol-substituted ring (blue).

1H *N*-(4-fluorophenyl)-*N*-(2-hydroxyphenyl)formamide

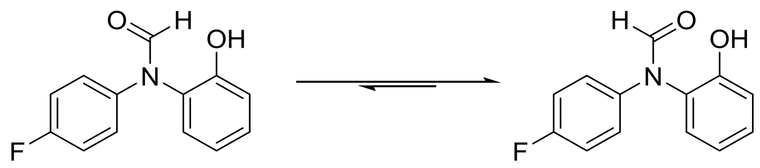

HMBC (600/151 MHz, $CDCl_3$)

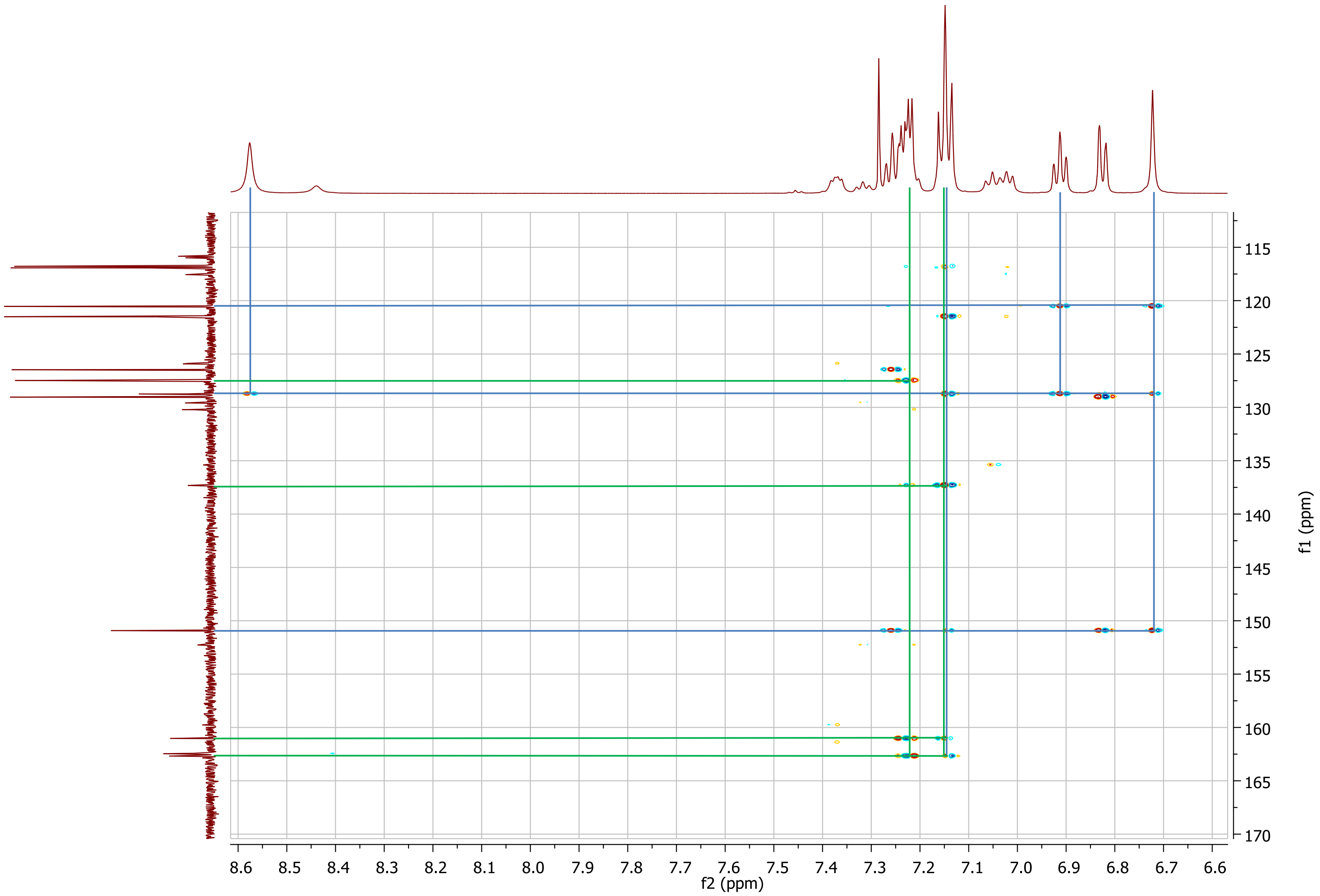

HMBC (500/126 MHz, $CDCl_3$)

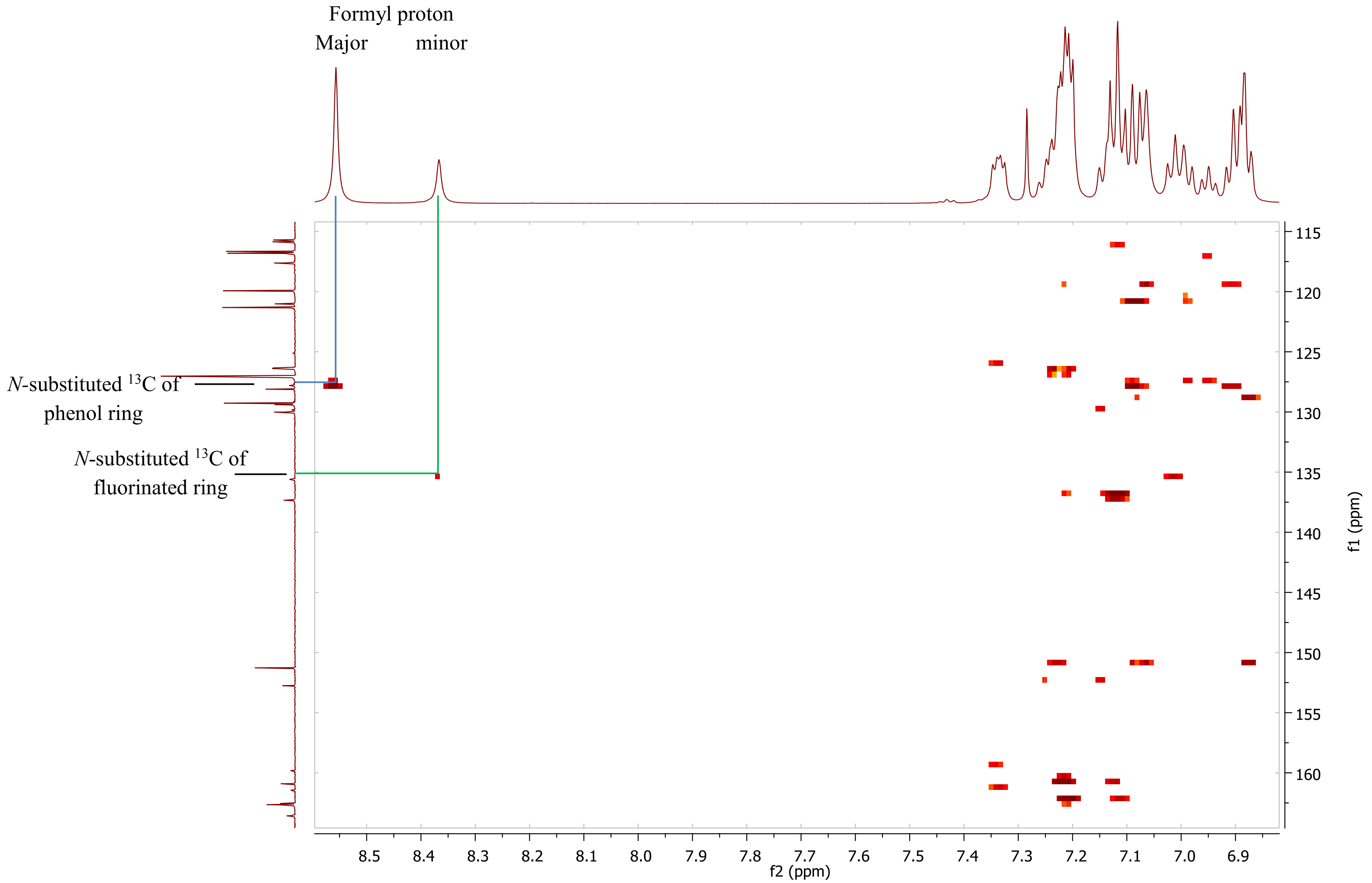

HSQC (500/150 MHz, $CDCl_3$)

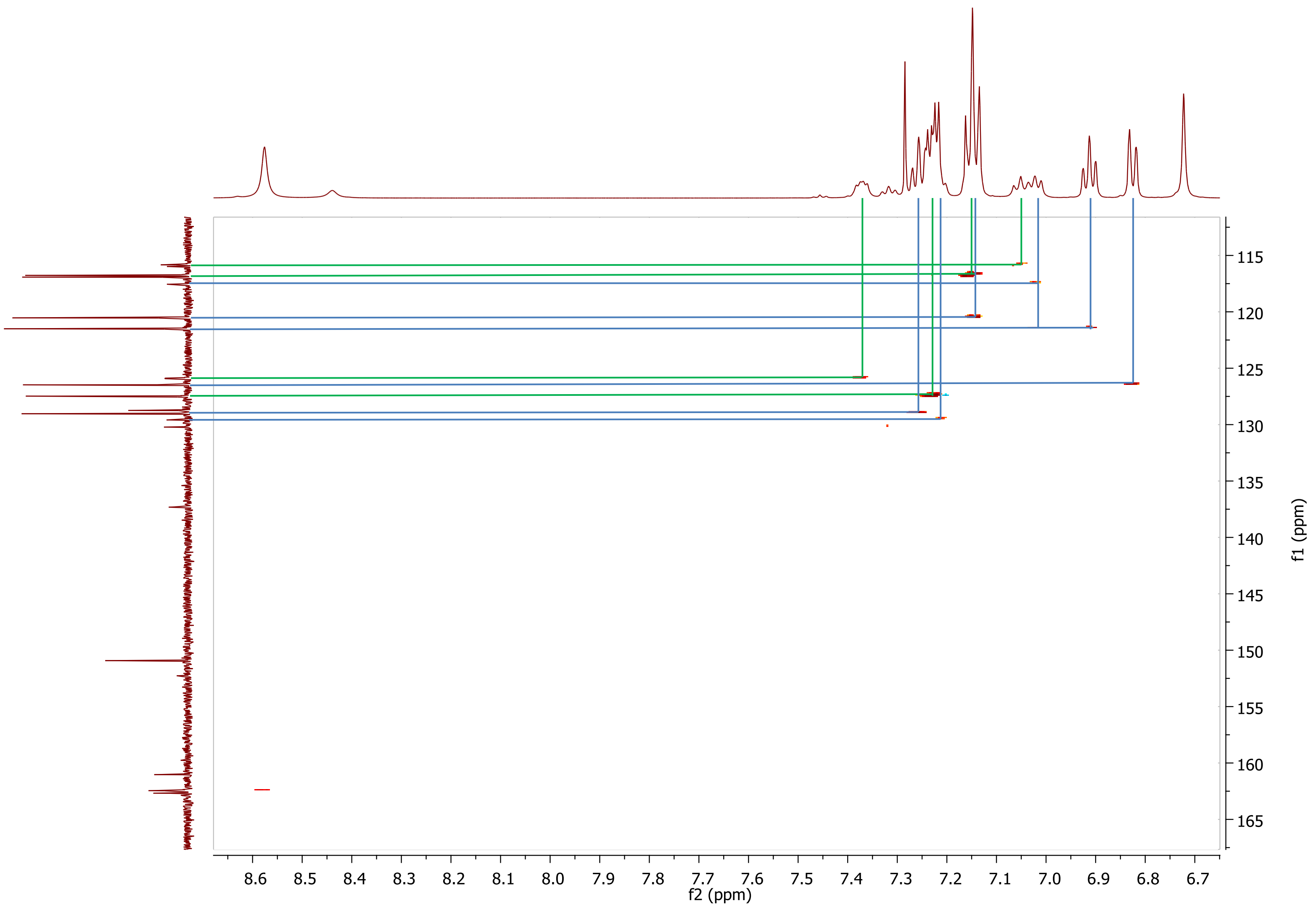

COSY (600 MHz, CDCl3)

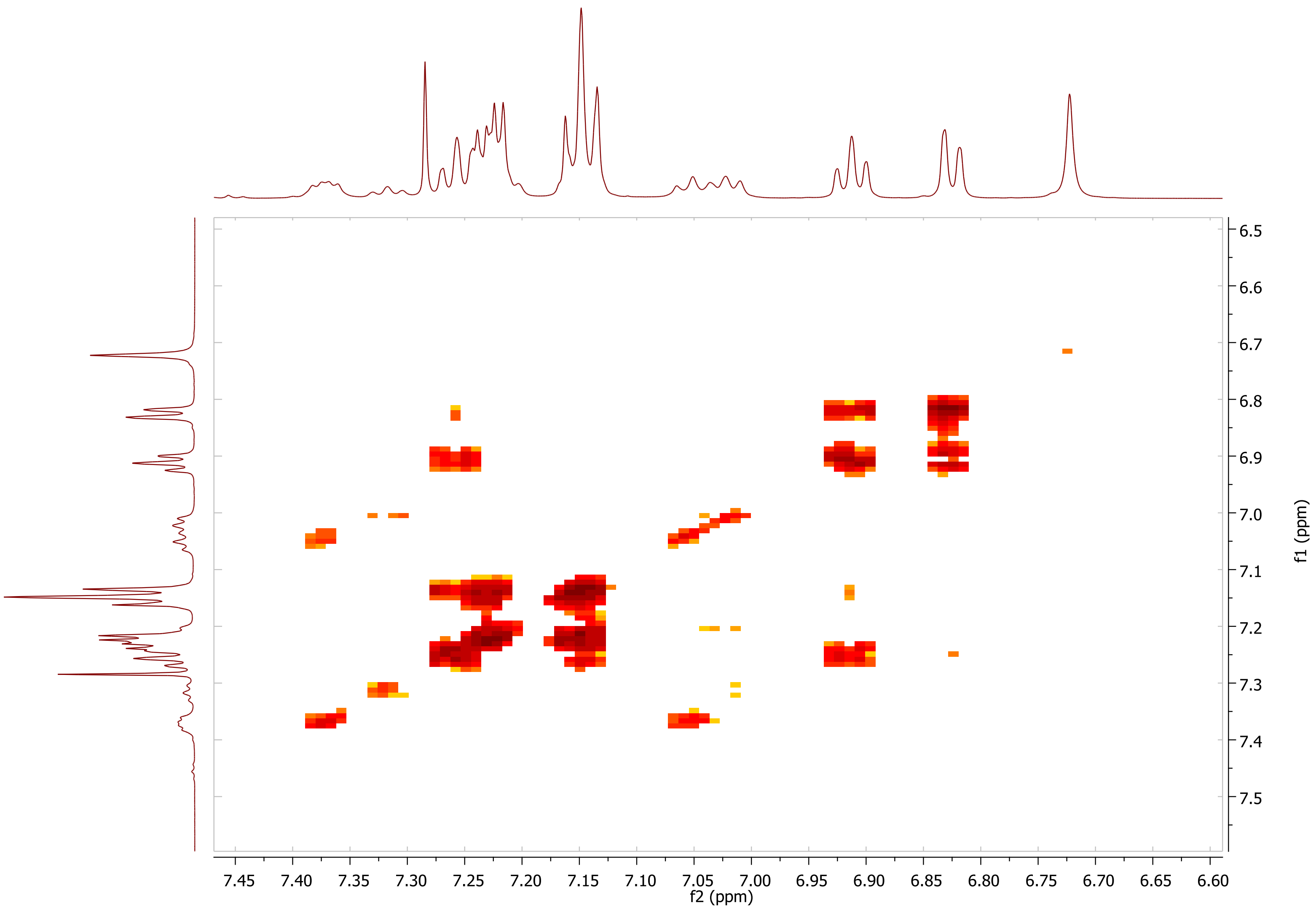

NOESY (600 MHz, $CDCl_3$)

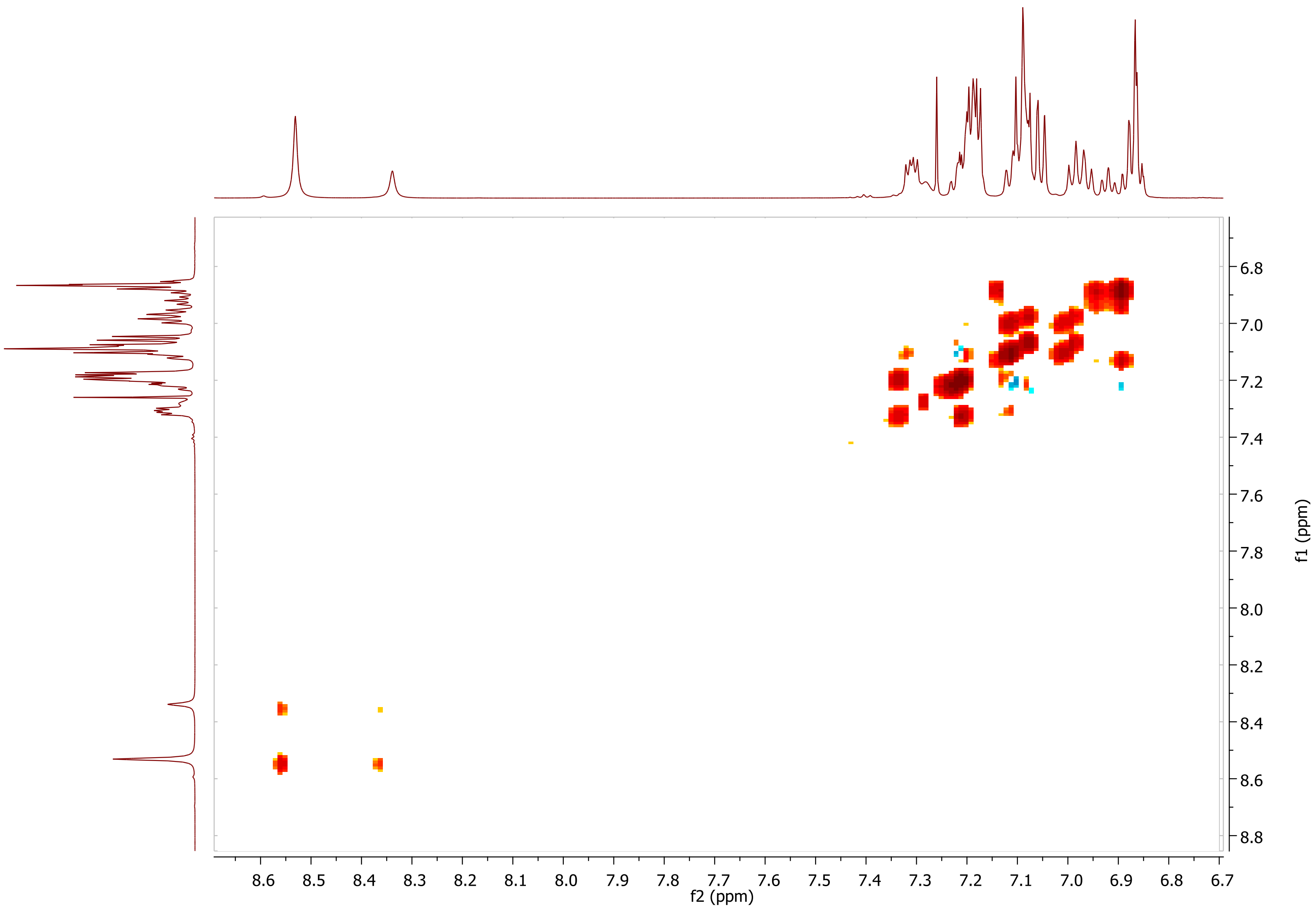

1D Spectra and Partial Assignment

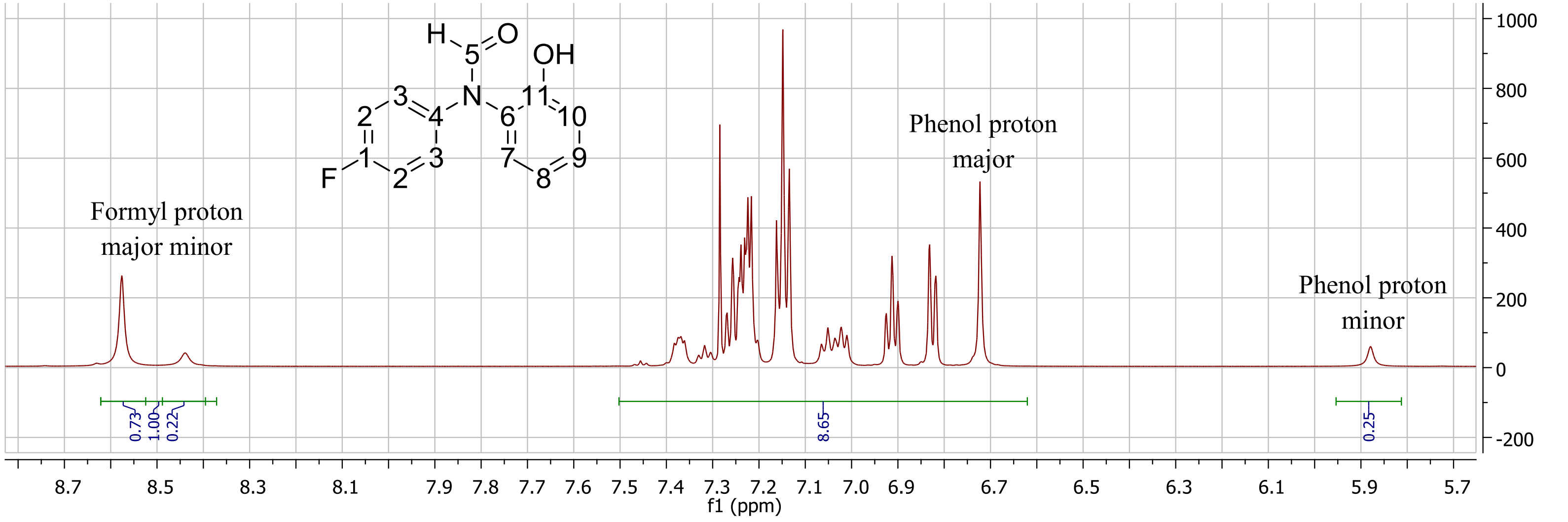

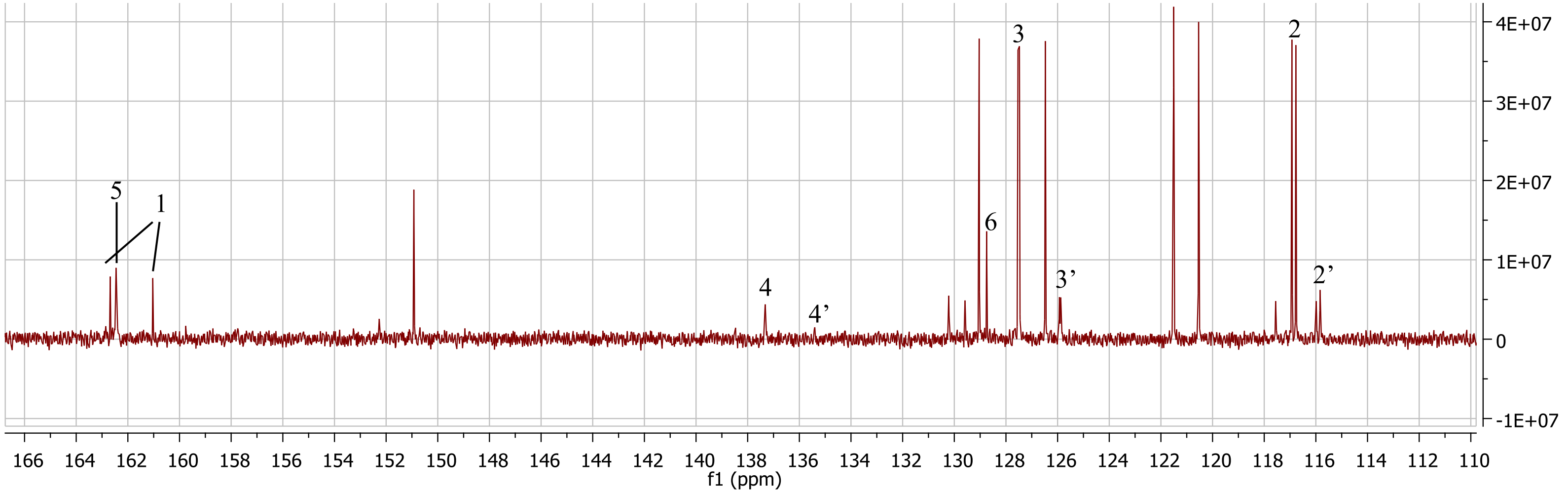

N-(2,3-dihydroxyphenyl)-N-(4-fluorophenyl)formamide

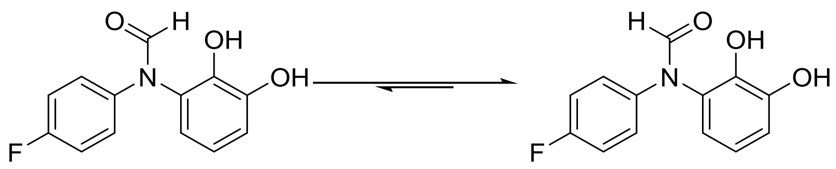

HMBC (500/126 MHz, $CDCl_3$):

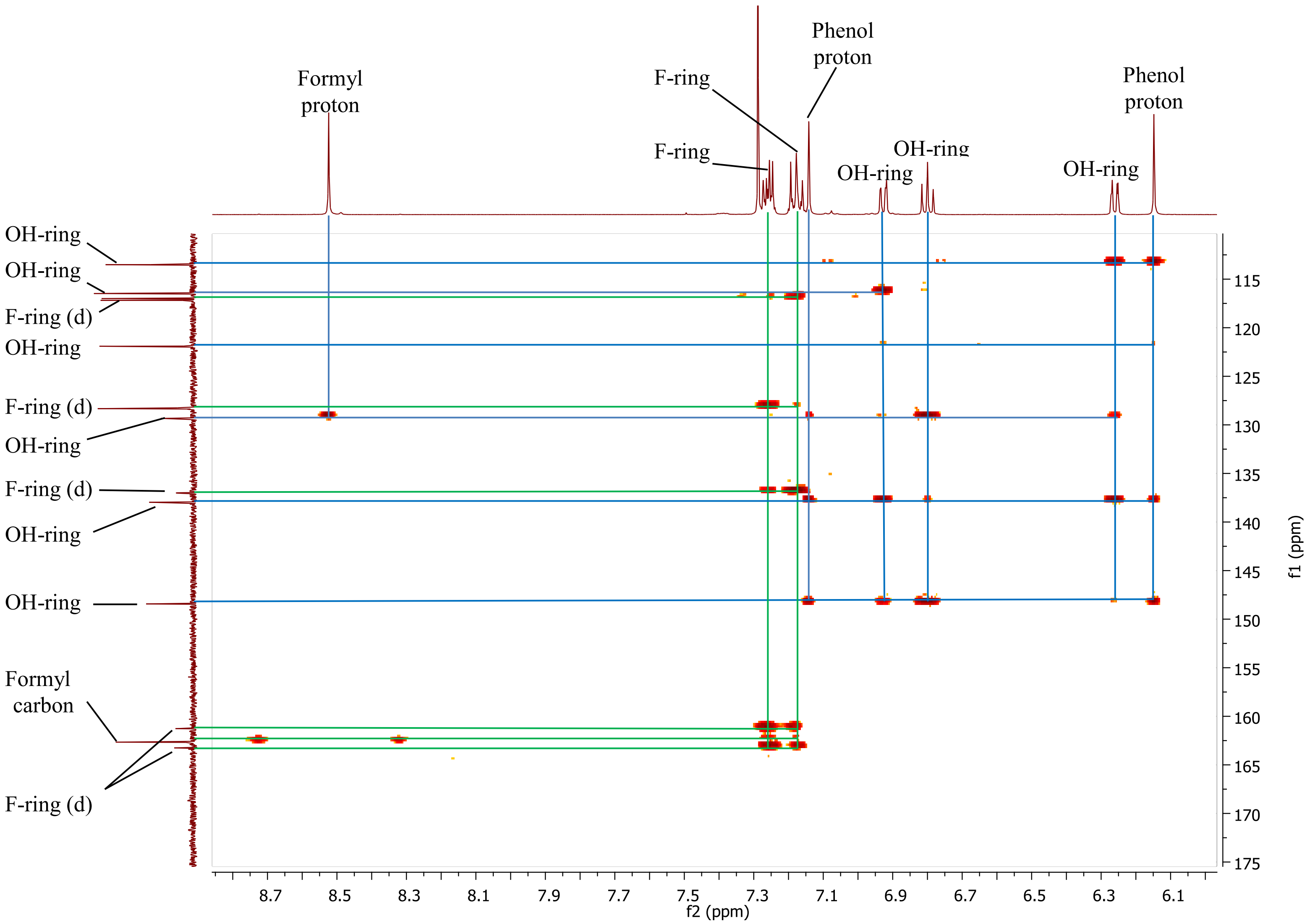

HSQC (600/150 MHz, $CDCl_3$)

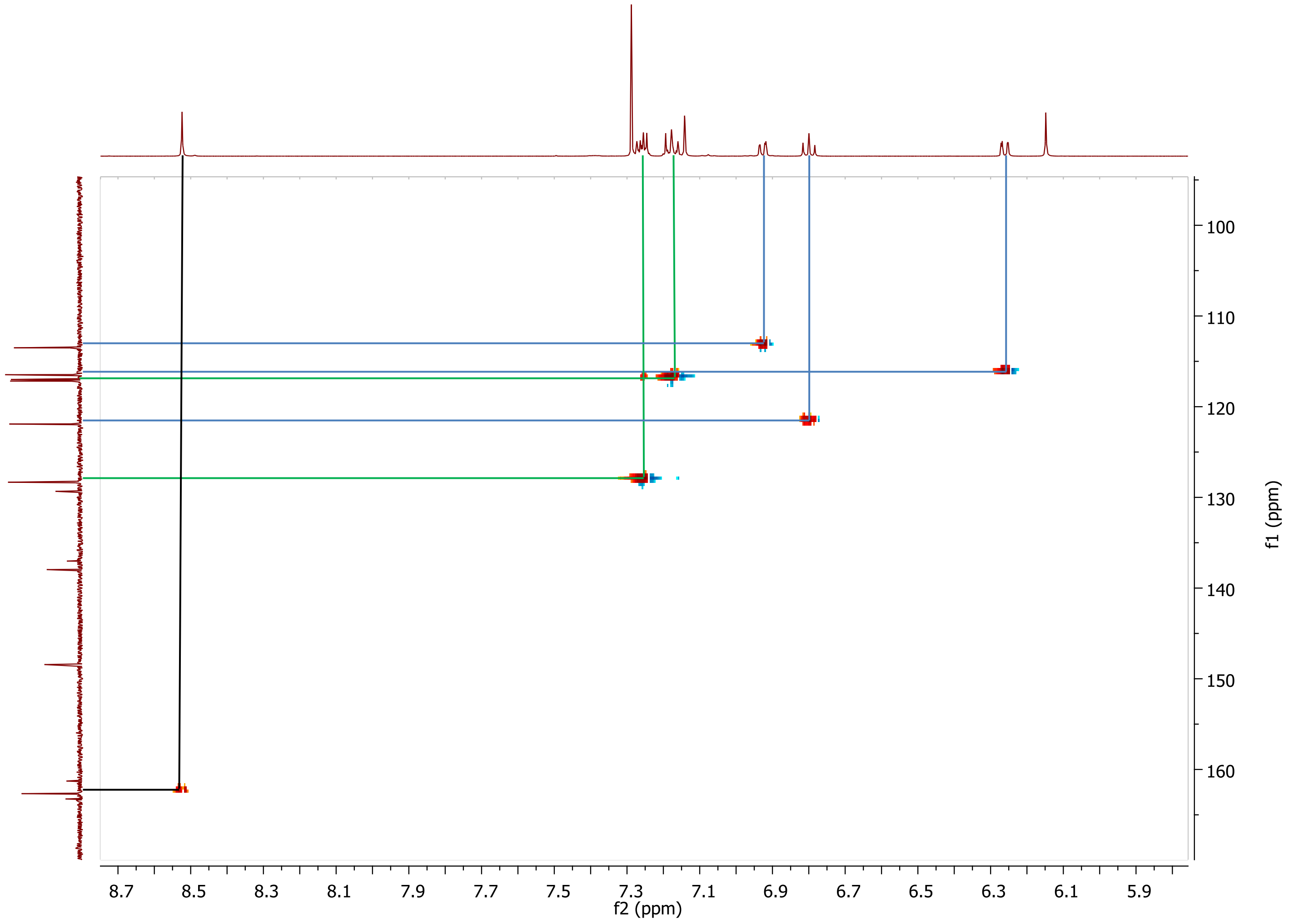

COSY (600 MHz, $CDCl_3$)

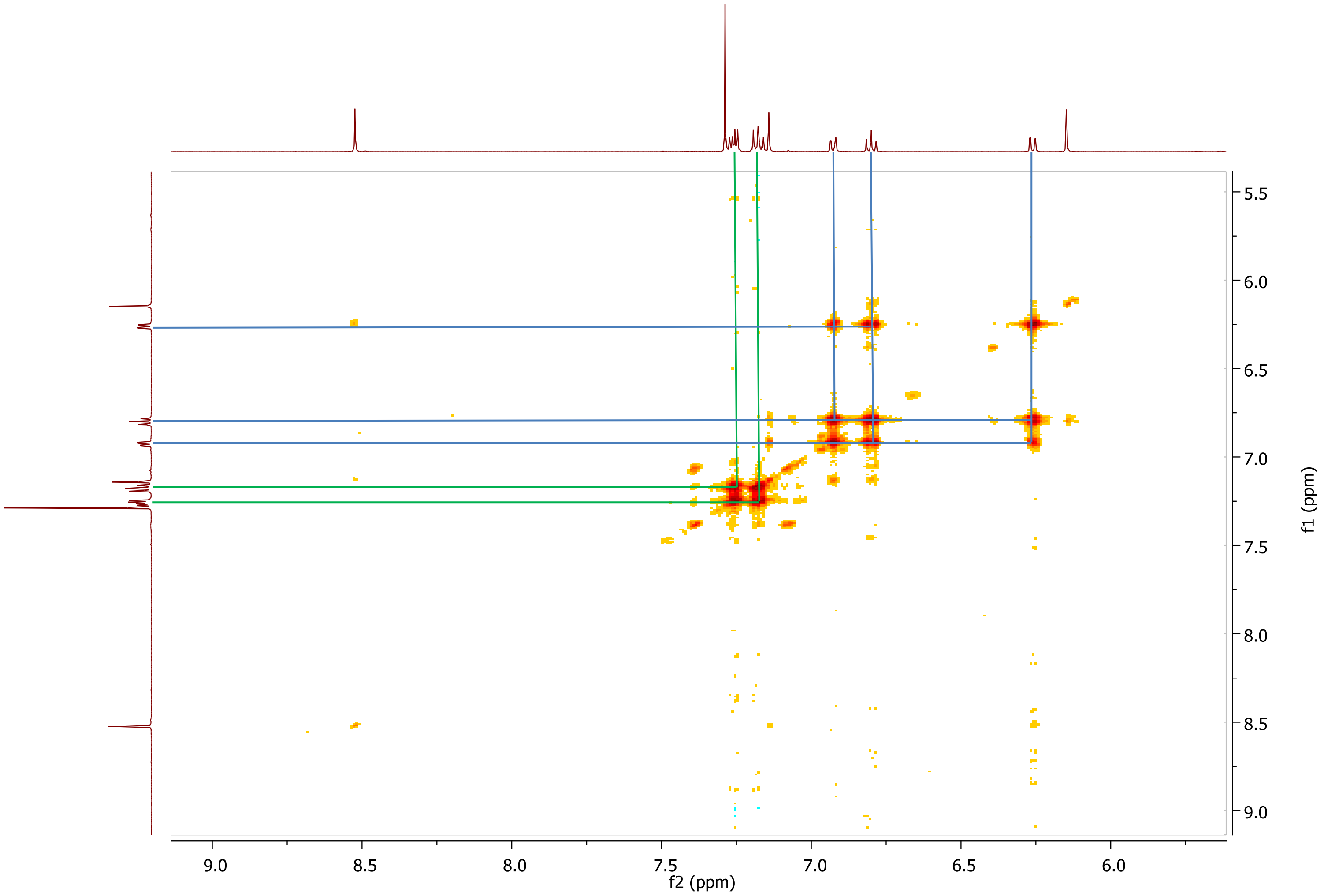

NOESY (600 MHz, $CDCl_3$)

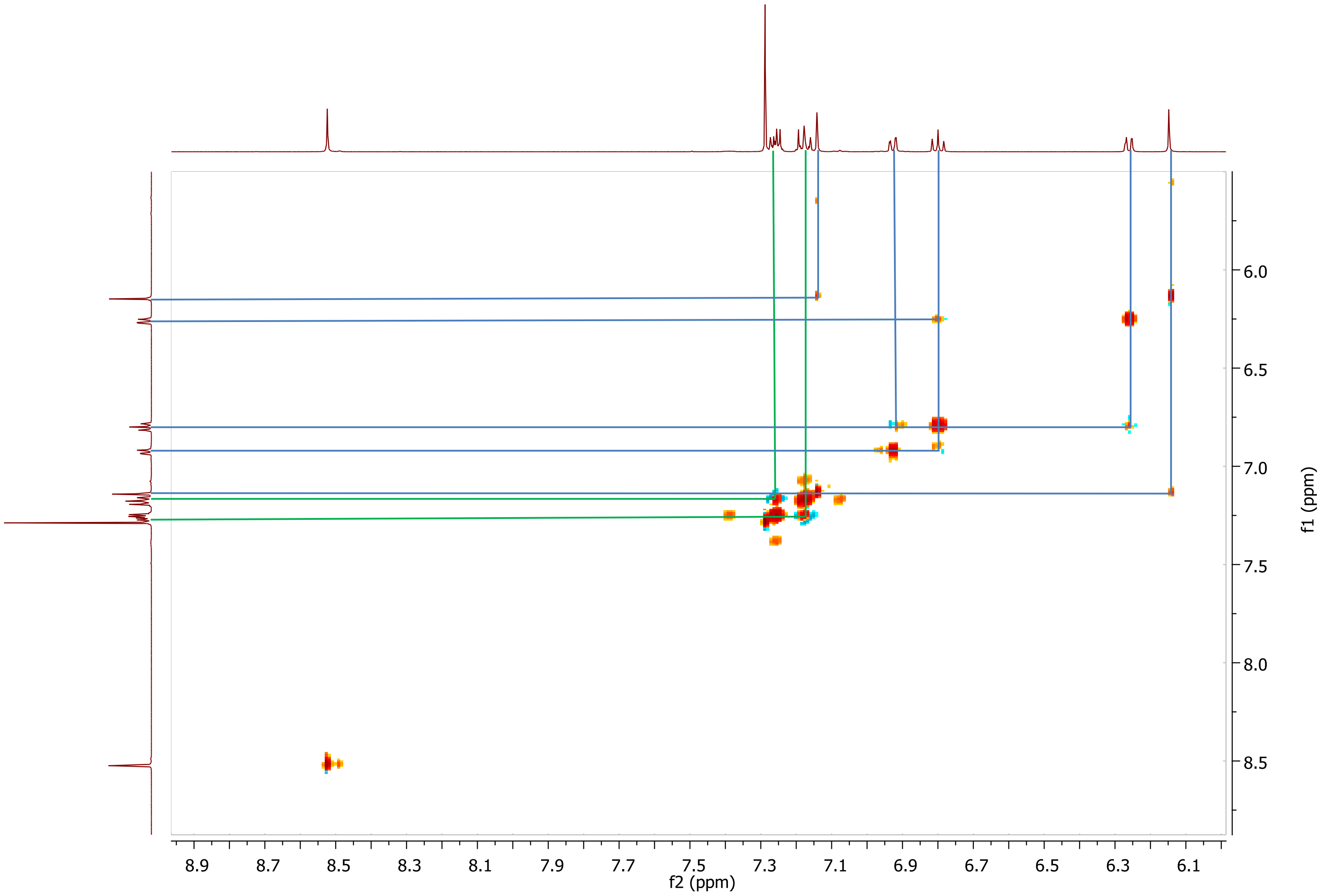

NMR Assignment

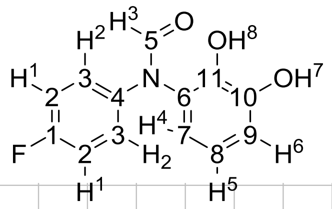

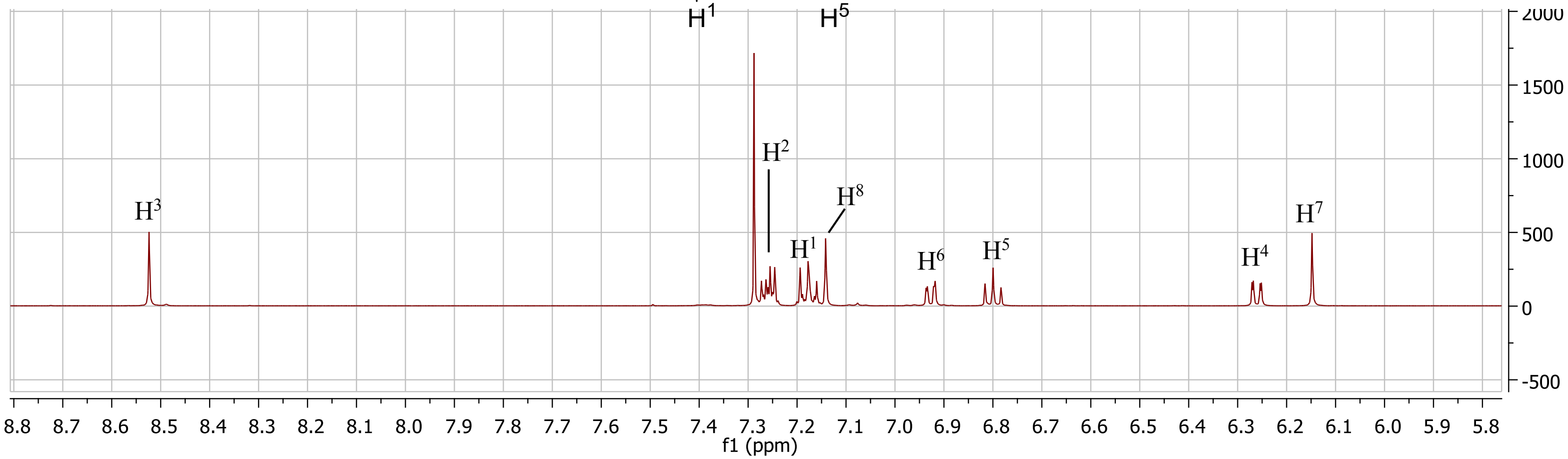

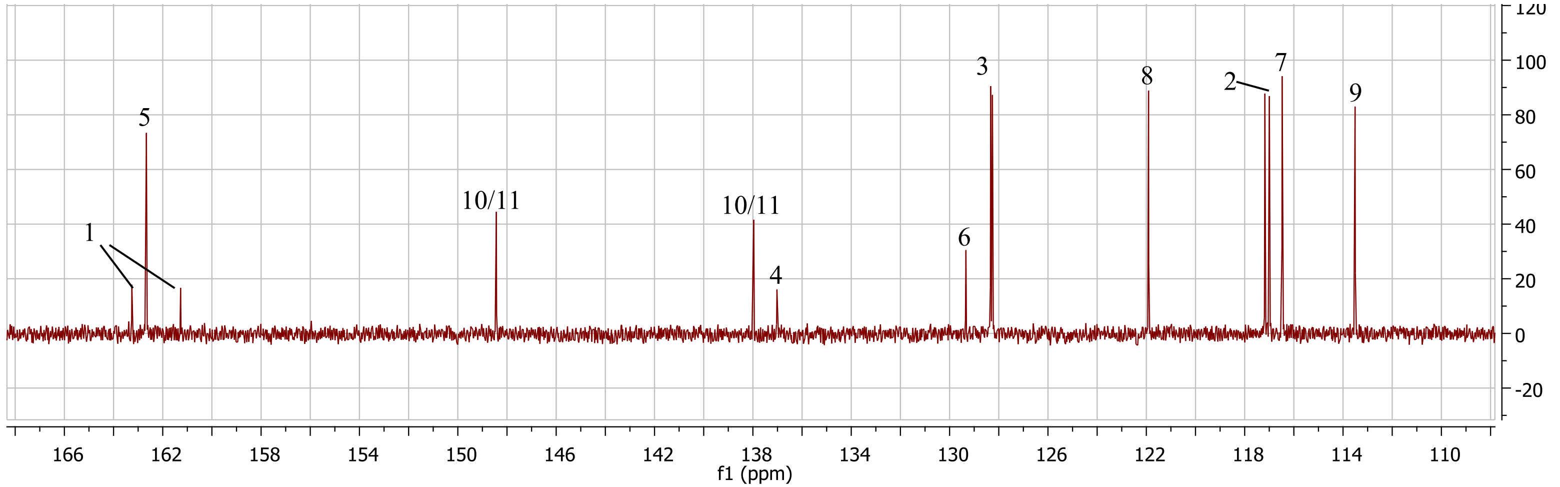

N-(4-fluorophenyl)-N-(2,3,4-trihydroxyphenyl)formamide

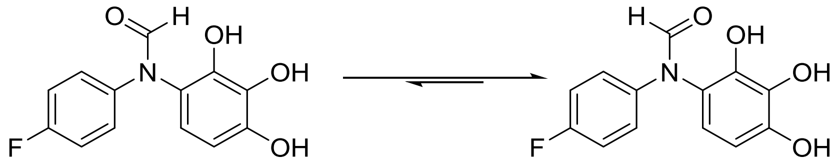

HMBC (500/126 MHz, $CDCl_3$)

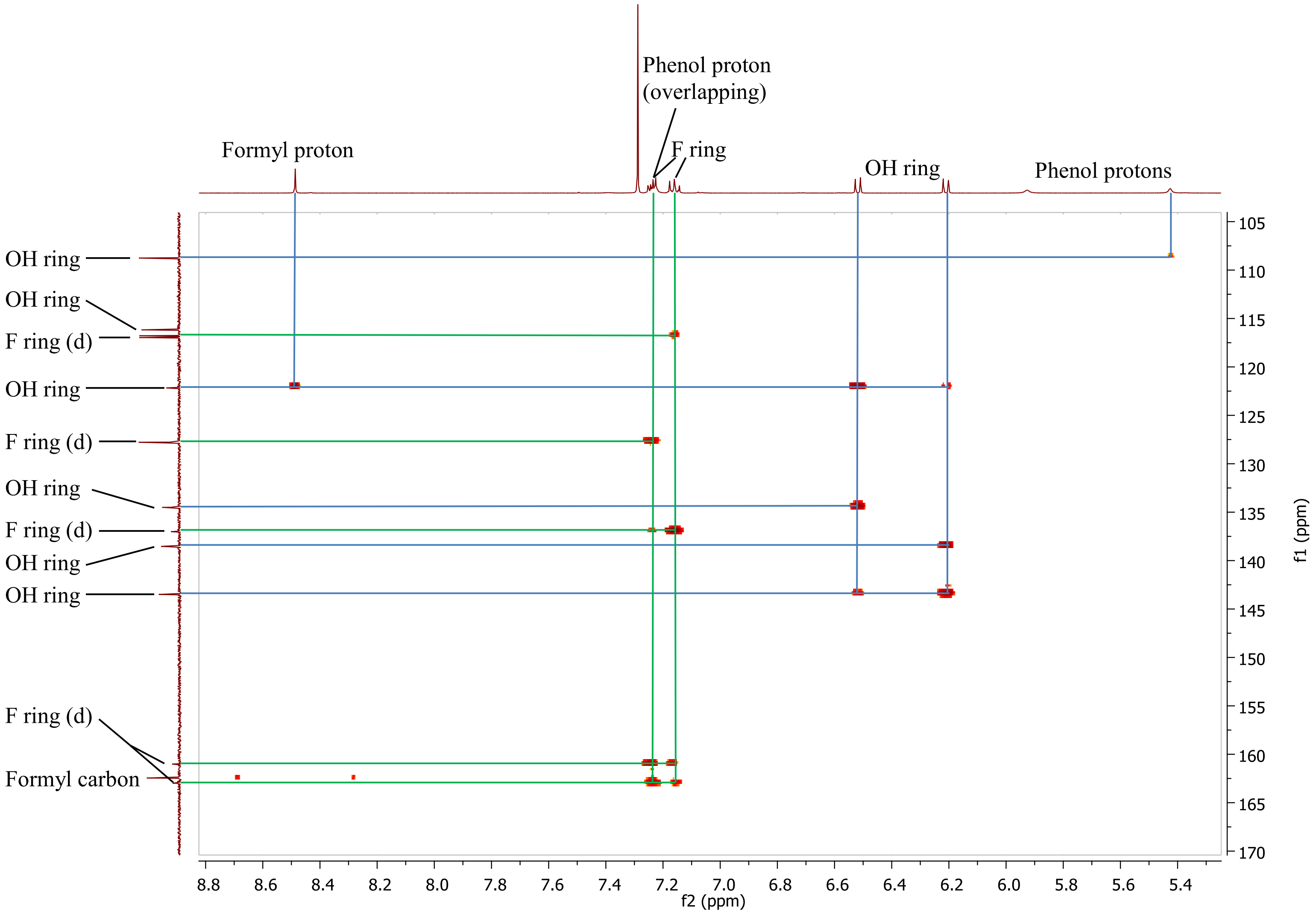

HSQC (500/126 MHz, $CDCl_3$)

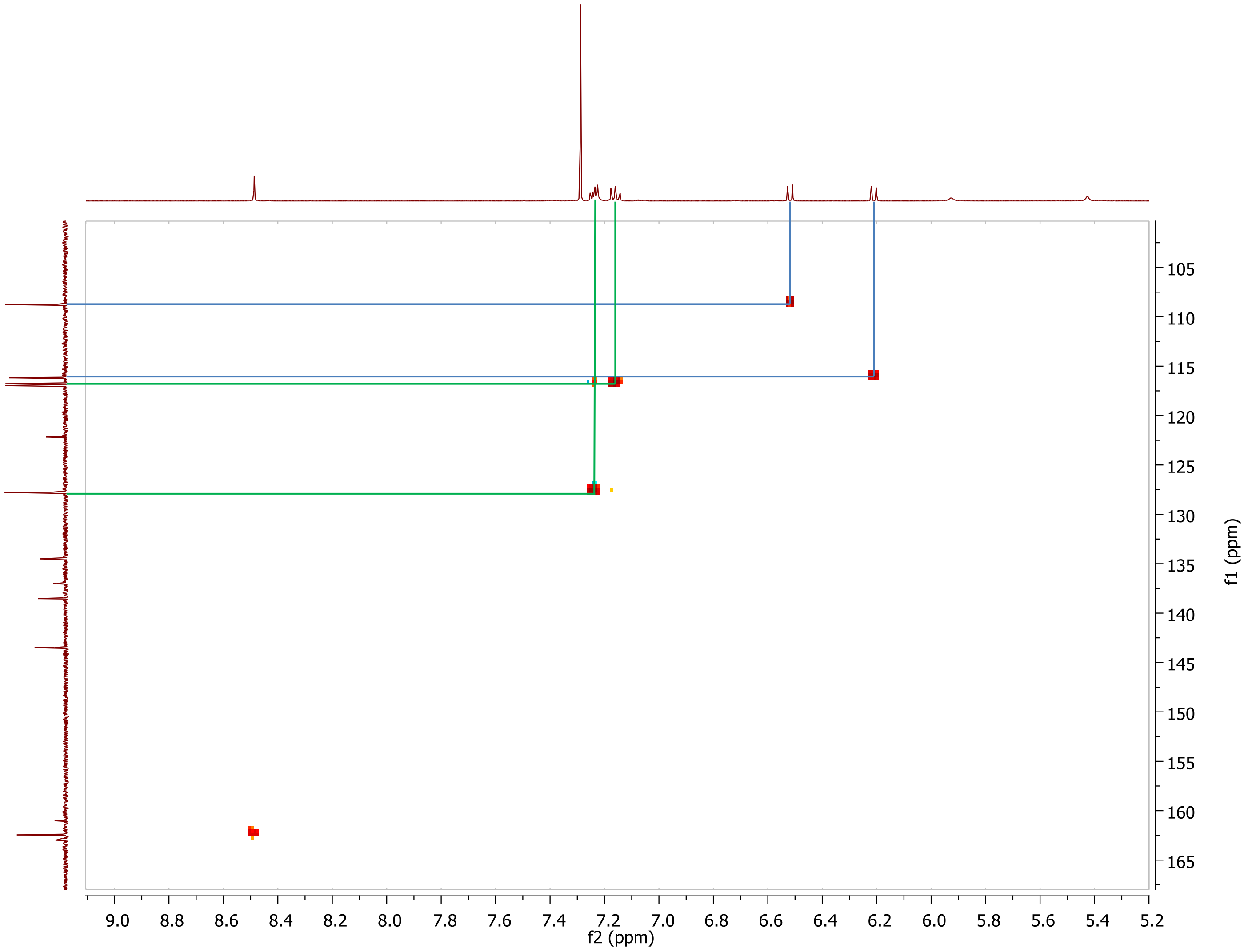

COSY (500 MHz, $CDCl_3$)

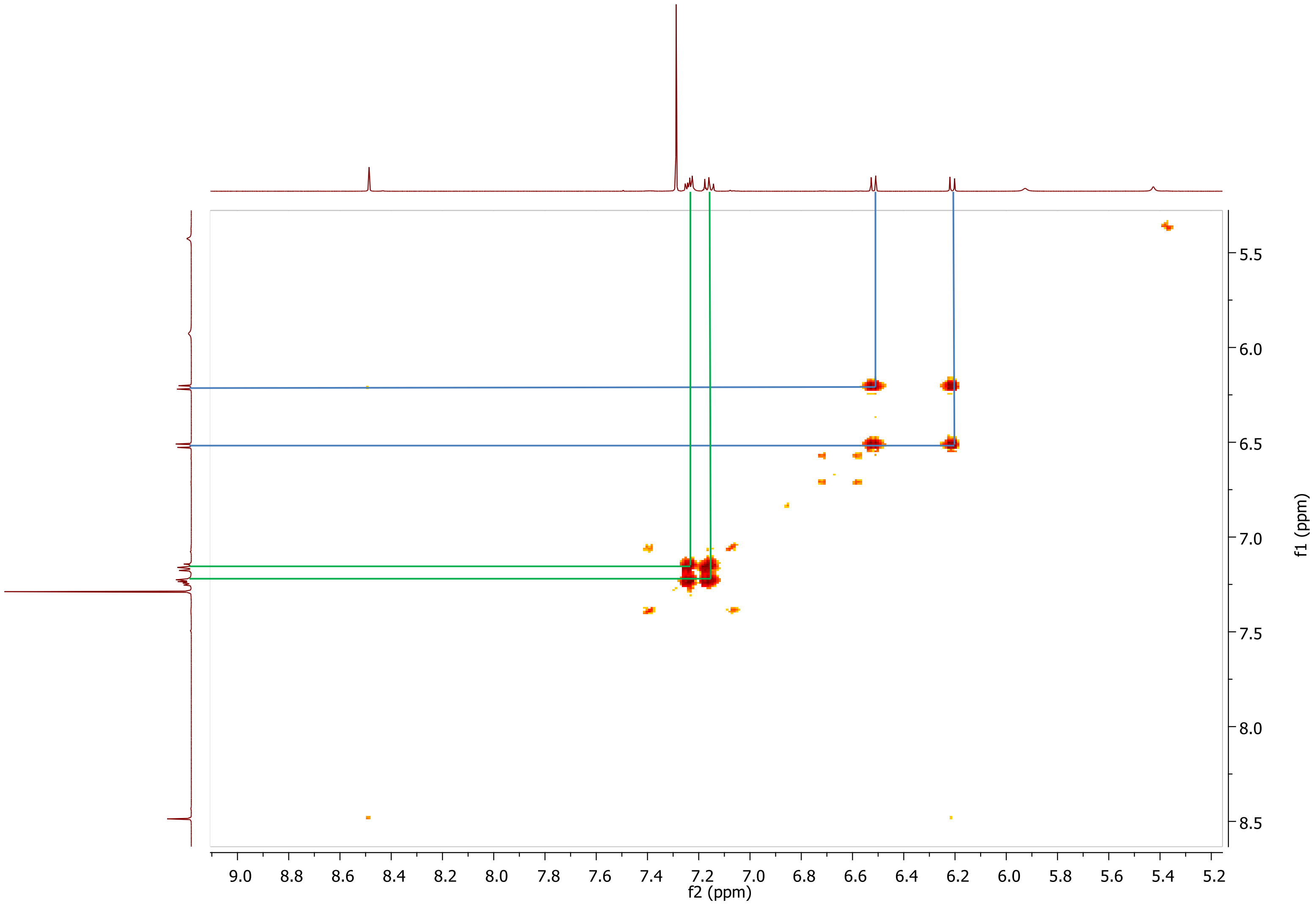

NOESY (500 MHz, $CDCl_3$)

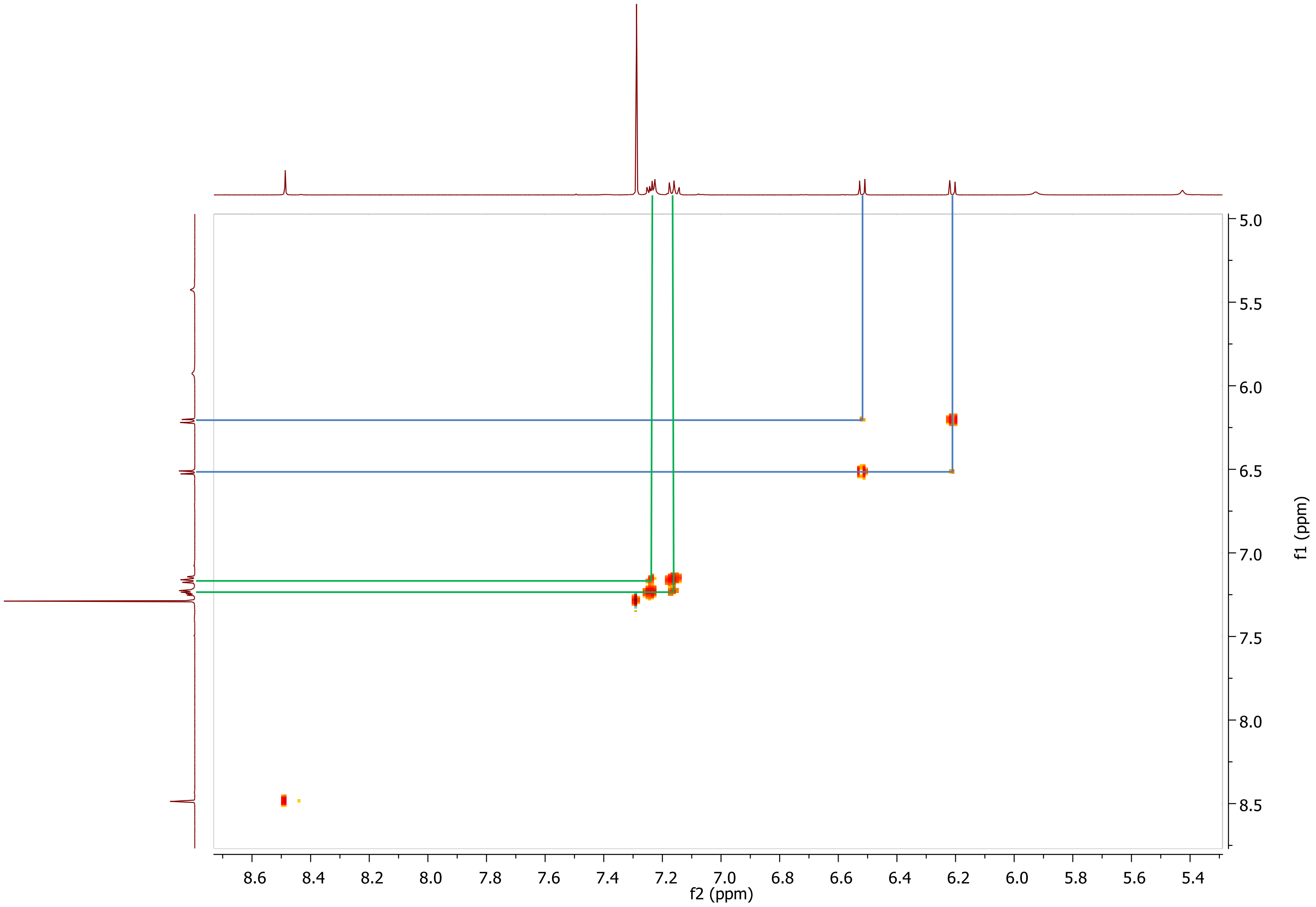

NMR Assignment

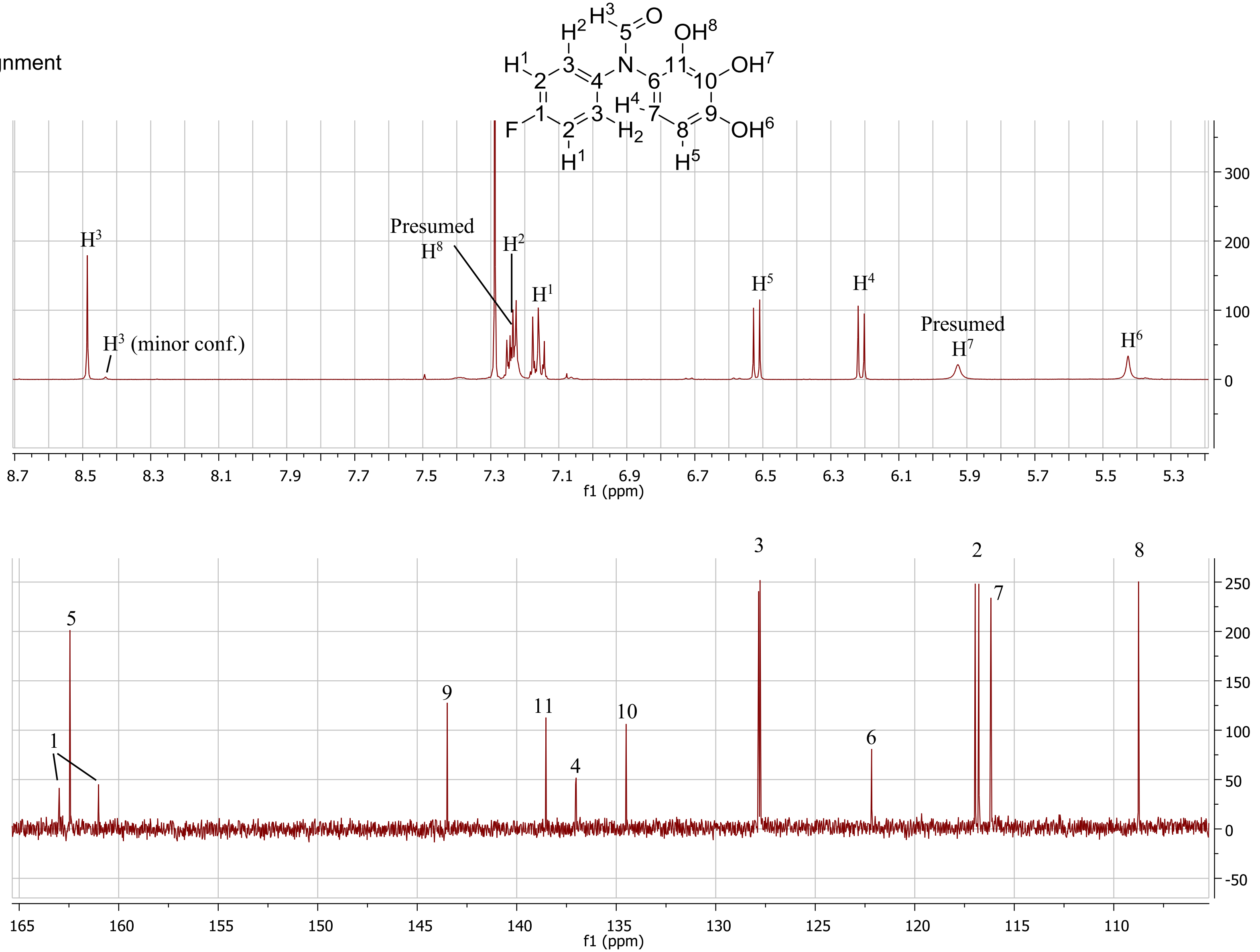

8. Supporting References

1. K. B. Muchowska, C. Adam, I. K. Mati and S. L. Cockroft *J. Am. Chem. Soc.*, 2013, **135**, 9976-9979.
2. I. K. Mati, C. Adam and S. L. Cockroft *Chem. Sci.* 2013, **4**, 3965-3972.
3. Gaussian 09, Revision A.02, M. J. Frisch, G. W. Trucks, H. B. Schlegel, G. E. Scuseria, M. A. Robb, J. R. Cheeseman, G. Scalmani, V. Barone, G. A. Petersson, H. Nakatsuji, X. Li, M. Caricato, A. Marenich, J. Bloino, B. G. Janesko, R. Gomperts, B. Mennucci, H. P. Hratchian, J. V. Ortiz, A. F. Izmaylov, J. L. Sonnenberg, D. Williams-Young, F. Ding, F. Lipparini, F. Egidi, J. Goings, B. Peng, A. Petrone, T. Henderson, D. Ranasinghe, V. G. Zakrzewski, J. Gao, N. Rega, G. Zheng, W. Liang, M. Hada, M. Ehara, K. Toyota, R. Fukuda, J. Hasegawa, M. Ishida, T. Nakajima, Y. Honda, O. Kitao, H. Nakai, T. Vreven, K. Throssell, J. A. Montgomery, Jr., J. E. Peralta, F. Ogliaro, M. Bearpark, J. J. Heyd, E. Brothers, K. N. Kudin, V. N. Staroverov, T. Keith, R. Kobayashi, J. Normand, K. Raghavachari, A. Rendell, J. C. Burant, S. S. Iyengar, J. Tomasi, M. Cossi, J. M. Millam, M. Klene, C. Adamo, R. Cammi, J. W. Ochterski, R. L. Martin, K. Morokuma, O. Farkas, J. B. Foresman, and D. J. Fox, Gaussian, Inc., Wallingford CT, 2016.
